# Harm from Residential Indoor Air Contaminants

**DOI:** 10.1021/acs.est.3c07374

**Published:** 2023-12-27

**Authors:** Giobertti Morantes, Benjamin Jones, Constanza Molina, Max H. Sherman

**Affiliations:** † Department of Architecture and Built Environment, University of Nottingham, Nottingham NG7 2RD, U.K.; ‡ Escuela de Construcción Civil, Pontificia Universidad Católica de Chile, Avenida Vicuña Mackenna 4860, Macul, Santiago 7820436, Chile

**Keywords:** DALY, dwelling, harm intensity, harm budget, ranking, acceptable indoor air quality

## Abstract

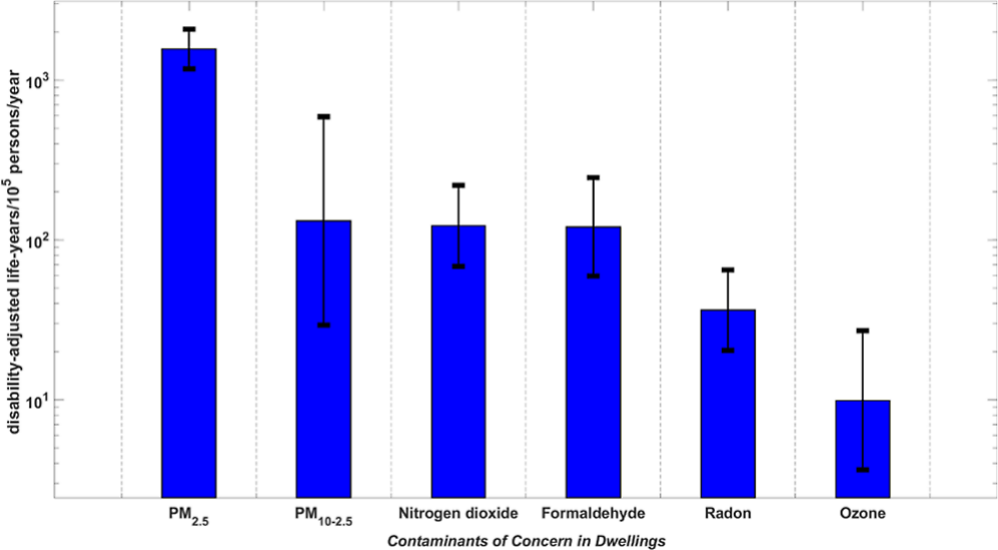

This study presents a health-centered approach to quantify and compare the chronic harm caused by indoor air contaminants using disability-adjusted life-year (DALY). The aim is to understand the chronic harm caused by airborne contaminants in dwellings and identify the most harmful. Epidemiological and toxicological evidence of population morbidity and mortality is used to determine harm intensities, a metric of chronic harm per unit of contaminant concentration. Uncertainty is evaluated in the concentrations of 45 indoor air contaminants commonly found in dwellings. Chronic harm is estimated from the harm intensities and the concentrations. The most harmful contaminants in dwellings are PM_2.5_, PM_10–2.5_, NO_2_, formaldehyde, radon, and O_3_, accounting for over 99% of total median harm of 2200 DALYs/10^5^ person/year. The chronic harm caused by all airborne contaminants in dwellings accounts for 7% of the total global burden from all diseases.

## Abstract 2

This study aims to quantify chronic harm from indoor air contaminants using a harm intensity metric and identify the most harmful contaminants in dwellings and propose a harm budget to regulate acceptable IAQ.

## 1 Introduction

There is strong evidence that exposure to harmful airborne contaminants commonly found in dwellings, such as volatile organic compounds (VOCs), particulate matter (PM), biological aerosols (mold), and radiological contaminants (radon), makes a significant contribution to the global burden of disease.^1,2^ People are particularly susceptible to these contaminants as they tend to spend up to 90% of their time indoors, mostly at home.^3−5^ To maximize the utility of harm reduction measures, indoor contaminants should be identified, ranked, and judged based on the harm they cause and the likelihood of their presence in indoor air. Health-centered metrics, such as the disability-adjusted life-year (DALY), can be used to estimate chronic harm caused by individual contaminants, and their individual magnitudes can be used to rank their impact of indoor air contaminants on population morbidity and mortality.^6−9^ Hereon, the chronic harm metric is the annual number of DALYs for a cohort of people.

Existing air pollution health risk assessment (AP-HRA) tools^10^ and life cycle impact assessment (LCIA) methodologies^11^ employ the DALY metric to quantify the chronic health impacts of airborne contaminants in indoor environments. AP-HRA tools estimate DALYs by relating observed changes in the incidence of disease in a population to estimate the burden of disease,^9,12−14^ whereas LCIAs apply effect factors (EFs), which are the number of DALYs per unit of mass intake of a contaminant.^15−17^ These assessments can use either toxicology or epidemiology research data or both. An epidemiology-based approach is derived from epidemiological data, such as risk estimates, the population attributable fraction, or disease incidence rates, whereas a toxicology-based approach is derived from toxicological data, such as the median effective dose (ED50). It is important to note that AP-HRAs are epidemiology-based, while LCIAs can use EFs derived from epidemiology or toxicology research depending on the data available for the contaminant of interest.

In 2012, a methodology was proposed by Logue et al.^18^ that combined epidemiology-based disease incidence and toxicology-based EFs to estimate the harm caused by air contaminants in dwellings. It defined an intake-incidence DALY (IND) method and an intake-DALY (ID) method to estimate the average population health costs associated with the chronic inhalation of common airborne contaminants in U.S. dwellings. The IND method is similar to the epidemiology-based approach in AP-HRA tools, while the ID method is similar to toxicology-based approaches used in LCIA methodologies. The Logue method significantly advanced the understanding of air quality in buildings,^19−24^ but it has some limitations: (i) it is primarily based on contaminant concentrations found only in U.S. dwellings; (ii) it reports large uncertainties in its estimation of harm; (iii) the epidemiological and toxicological data it uses are from the decade prior to 2010 and so they are due for revision; and (iv) the basis of some underlying assumptions that underpin their methods is not fully explained. Therefore, there is a need to update the method using epidemiological and toxicological research and indoor measurement data published in the past decade and to expand the scope of the analysis to include dwellings outside the United States.

Morantes et al. revisited the Logue models,^25^ populating them with more up-to-date data. They identified a need for improvements that include a unified harm metric that considers contaminant concentrations to replace the IND and ID approaches, a consolidation of data obtained from a global review of the global burden of disease and incidence into a single burden of disease database to replace damage factors (DFs) and incidence data, the linearization of the IND method, and the simplification and separation of the IND and ID methods. This work addresses these issues.

There is currently no recognized process for selecting priority contaminants to be controlled by indoor air quality (IAQ) standards and regulations.^26−31^ Accordingly, there is a need to identify contaminants that are both the most harmful and the most prevalent so that they are prioritized as *Contaminants of Concern* (CoCs) and targeted for removal. The CoC term aligns closely with *Priority Contaminants* or *Criteria Pollutants*, but is distinct from the term *Contaminants of Emerging Concern* (CECs) used in water-related research.^32^

Existing IAQ metrics rely on contaminant concentrations^1,33^ but do not directly consider associated health risks. To address this, we introduce the concept of harm intensity (HI) with units of DALY/concentration/person/year, which links chronic harm (DALY/person/year) to the concentrations of airborne contaminants to which people are exposed to. This is like the concept of the inhalation unit risk estimate used by the U.S. EPA to link lifetime cancer risk with exposure to an indoor air contaminant concentration.^34^ Tentative steps have been made before to relate DALYs to a concentration unit for ventilation and IAQ^28,30^ and for PM_2.5_ in LCIA,^35^ but the concept of HI has not been defined previously. The power of the metric is its simplicity and its application to any environment because harm is solely a function of the contaminant. Therefore, when contaminant concentrations that represent chronic exposures are known in an environment, it is possible to use the HI metric to calculate the total harm they cause and identify those that are the most harmful.

There are many risk factors in daily life that have an acceptable level of harm in a population, such as taking a mode of transport or consuming a particular food or drink. The harm they pose to a population can be quantified using the DALY metric^8^ and their value could represent the current acceptable magnitude of harm. Therefore, DALY can be used to set a harm budget for airborne contaminants and to quantify the quality of indoor air in buildings. The harm budget is an interpretation of the IAQ equivalence principle,^28,29^ where a harm limit is set, and then any combination of contaminant concentrations that keeps the contaminant harm below that limit is allowed. The ANSI/ASHRAE Standard 62.2^36^ proposes a definition for *acceptable* IAQ (AIAQ) in dwellings, but there is still a need for a quantitative definition of AIAQ. This is addressed by a harm budget.

The aim of this article is to identify the most harmful airborne contaminants in dwellings today so that they can be prioritized for removal and be used to define a harm budget. To do this, we (i) use existing epidemiological and toxicological data to determine the HIs of common contaminants, (ii) determine contaminant concentrations in dwellings, (iii) combine the HIs and concentrations for each contaminant to determine the harm caused from typical exposures to them, and (iv) rank the contaminants by the magnitude of the harm they cause. The results will inform the development of health policies, building codes and regulations, and the design and operation of buildings.

## 2 Methods

To meet the aim, it is necessary to develop an expression for the chronic harm (in DALY/person/year) caused by the inhalation of a specific airborne contaminant (indicated by the subscript *i*) as a function of HI, HI_*i*_, and concentration *C*_*i*_.
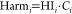
1Generally, indoor contaminant concentrations are reported in micrograms per cubic meter (μg/m^3^), but some contaminants have other units, such as Bq/m^3^ for radon and cfu/m^3^ for mold spores. Therefore, for most airborne contaminants, HI_*i*_ has units of DALY/μg/m^3^/person/year.

Indoor air comprises contaminant mixtures,^37^ and although synergies for some contaminant combinations exist,^38−41^ evidence of chronic synergies remains limited.^42,43^ When synergies are identified, they are found to be rare at the concentrations typically found in buildings.^44−47^ Knowledge of air contaminant synergies in LCA is also scarce.^11^ Most approaches for multiple contaminant exposures follow the *Concentration Addition* principle, and components are combined additively.^48−50^ Therefore, we also apply the additive approach to our model, aligning it with existing risk assessment methods.^1,51,52^ Furthermore, when evaluating the total harm (in DALYs) from exposure to a mixture of indoor air contaminants, it is common to use an additive approach to combine the health impacts from each contaminant.^19,53−56^

Therefore, the harm from any number of contaminants can be summed to obtain the *total* harm they cause, where
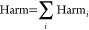
2

The individual contaminant harms can be compared against the total harm to determine those that contribute the most. This allows the most harmful pollutants to be identified and designated CoCs.

Equation 2 is the all-cause harm that aggregates the health impacts from all diseases that exposure to a contaminant might induce, which is the metric we are ultimately interested in. While some data sources may provide all-cause information, some are disaggregated by disease so that the all-cause harm becomes the sum of the harms for each disease, as
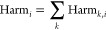
3where the subscript *k* denotes a specific disease. Then, Harm_*k*,*i*_ can be defined as a function of the HI for each disease, HI_*k*,*i*_, where
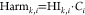
4Summing specific contaminant-related diseases is well-established in air pollution assessment methods for approximating the total harm (in DALYs).^8,18,35,53,54,57−59^ Logue et al.^18^ combined the carcinogenic and noncarcinogenic harm effects identified by toxicology research.^19,24^ In epidemiology, all-cause mortality (*k*) is often used to summarize the collective impact of major diseases resulting from long-term exposures.^1^ However, without morbidity, it gives a reasonable, but lower-bound, estimate of the all-cause HI.

We are generally following the characterization framework of life-cycle impact assessment (LCIA),^11^ which is rooted in toxicological and epidemiological research, and has been widely applied in studies of outdoor^35,53,54,57,58,60−64^ and indoor air pollution, particularly inside or near dwellings.^65−68^ LCIA considers many parameters, but the one that is most similar to the HI is the EF, which has the units of *DALY*/*kg*. The HI can be related to the EF using a breathing rate (BR), which can be assumed to be constant.
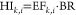
5BR is a standardized annual breathing rate of 5402 m^3^/person/year.^69−71^

Generating HIs for each disease and contaminant require the conversion of existing health data from the forms in which they are typically reported, which vary depending on the discipline they originate from. The data from toxicological and epidemiological studies are then examined in turn.

### 2.1 Toxicological Analysis

We reviewed the toxicological data for a wide range of relevant contaminants (Section 2.3) and calculated their median all-cause HIs and uncertainties.

Toxicological studies aim to determine the harmful effects of various contaminants on living organisms. Organisms are exposed to doses of contaminants to determine the quantal dose–response relationship that characterizes the distribution of responses to different doses in a population of individual organisms.^72^

A widely used statistical approach for estimating the response of a population to a toxic exposure is the *effective dose* (ED). Generally, the midpoint, or the 50% response level, is reported and is known as effective median dose, ED50.^72,73^ The current approach of the LCIA characterization framework is to use the ED50 to quantify the effect factor parameter.^15,74−79^ The ED is specific to a disease and contaminant and so is shown representing a cancerous or noncancerous effect for each contaminant of interest in LCIA. This approach is similar to the ID method of Logue et al.^18^

The toxicology-based characterization framework considers a DF (DALY/case), ED50 (kg), BR (m^3^/person/year), and a constant of 0.5 (cases) in a linear equation. This expression is used to determine HI (DALY/μg/m^3^/person/year) from toxicological data, where
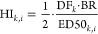
6

The data used to derive the toxicology-based HI parameters encompass the following: (i) the effective median dose-related data derived from USEtox 2.0;^74^ (ii) exposure factors for BRs;^70^ and (iii) the global burden of disease collaborative network for DFs.^8^ Details of the literature review, calculations, and other considered criteria are available in the Supporting Information.

### 2.2 Epidemiological Analysis

Epidemiology focuses on the patterns of disease and ill-health in a population.^80^ Epidemiological studies statistically link disease incidences to real-world exposures. They require substantive evidence and so provide less data on contaminants than toxicological studies.

The AP-HRA framework estimates the risks of exposure to air pollution. The risk of air pollution to health in a population is usually represented by a concentration–response function (CRF). The CRFs used in AP-HRA tools are typically based on the epidemiological evidence available for a specific health outcome and may be represented by linear or nonlinear forms.^10,81^ This approach is similar to the IND approach of Logue et al.^18^

The incidence rate is the prime estimate of risk in epidemiology,^80^ and so health risk assessments use health impact functions (HIFs) to estimate changes in outcome incidence. HIF methods require information that includes the size of the exposed population, baseline incidence rates for diseases associated with pollutants, baseline and exposure concentrations, and CRF estimates or relative risks for each contaminant-disease pair.^82^ Therefore, our epidemiological analyses account for a DF (DALY/case), a baseline incidence rate, γ_0_ (cases/person/year), and a risk factor, β (change/μg/m^3^) in a nonlinear equation that considers saturation at high exposures.
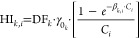
7The term in parentheses models the nonlinear, no lower threshold, saturation. When the equation is evaluated at the low concentrations normally expected in dwellings,^83,84^ it becomes an approximately linear expression that can be used to determine HIs (DALY/μg/m^3^/person/year) from epidemiological data, where
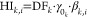
8

The epidemiological data are reviewed for the common contaminants described in Section 2.3, and the median all-cause HIs and their uncertainties are calculated. Further details of the literature review and calculations are given in Supporting Information B. Two sources are particularly important. To determine the beta parameter for risk derivation, we referred to the literature that compiled or reviewed risk estimates^1^ and then the disease-specific baseline incidence rates and DFs from the global burden of disease collaborative network.^8^

### 2.3 Representative Indoor Contaminant Concentrations (C_*i*_)

We define C_*i*_ as the median concentration of an airborne contaminant found in dwellings. Priority contaminants were selected from an existing list of 43 with the highest chronic health damage identified by Logue et al.^18^ Carbon monoxide was removed from the list because its effects are acute; ammonia, manganese, xylene (o), and xylene (m/p) were removed because of lack of toxicological evidence. PM_10_ and three other contaminants (1,3-butadiene, isoprene, and trichloroethylene) were added to reflect recent reviews of common airborne contaminants in dwellings.^85^ Additionally, we extended the analysis to include molds (spores) and radon. Measured Cladosporium spore concentrations were used as the mold indicator because it is a required input to the epi and tox models (for a discussion, see the Supporting Information). The list contains 44 contaminants, comprising semivolatile organic compounds, VOCs, metals, and the criteria pollutants, a group of six contaminants scrutinized by health assessments.^1,86^ To determine the uncertainty in the concentrations of these contaminants, we conducted a systematic review of peer-reviewed studies reporting indoor chronic exposure (periods >24 hours) in dwellings to the 44 air contaminants. A 45th contaminant is then added to the list by defining the *coarse fraction* of particulate matter (PM_10–2.5_) as the difference between respirable particle matter (PM_10_) and fine particle matter (PM_2.5_). PM_10–2.5_ is considered here because current guidelines tend to restrict PM_10_ concentrations to protect against the effects of exposure to PM_10–2.5_ and so considering them separately indicates the relative importance of different size fractions.^87^ More details on PM_10–2.5_ are provided in the Supporting Information. However, there is growing evidence that harm is inversely related to the size of particulate matter so smaller particles may be more hazardous.^87−89^

The primary data extracted from monitoring studies include concentration statistics and the country or world region in which they were measured. We include concentrations measured by fixed or portable samplers or monitors, followed by any analytical postprocessing method but excluded modeling studies. The review is designed to provide evidence of uncertainty in the median contaminant concentrations for typical dwellings from a nonspatially restrictive perspective. A more detailed analysis of the data by country or region, season, room, or temporal period is outside the scope of this study. A full description of the literature review is provided in the Supporting Information.

### 2.4 Total Harm

The harm attributable to chronic exposures is calculated by using eq 4 from the representative indoor concentrations and the HIs. The values of harm are used to rank the contaminants and identify CoC; see Section 3.3. These CoCs can then be used to regulate IAQ in dwellings. One way of doing this is to set a *harm budget*, the maximum harm that is expected in a reference scenario. An extensive analysis of different approaches to set a harm budget is beyond the scope of this paper, but an example is given in Appendix A and is used to provide a quantitative value for *acceptable* IAQ.

### 2.5 Parameter Distributions

All parameters described herein are always greater than zero, and many have broad distributions. Therefore, we assume that a log–normal function represents their distribution best using the median as their representative value and geometric standard deviation (GSD) as the uncertainty metric. This approach is consistent with established methodologies.^15,90,91^ A GSD quantifies uncertainty on a linear scale in log–normal distributions. It avoids scaling effects that can occur with variance-based measures.^92,93^

It is common to report central tendency estimates for concentration measurements in several different ways; for example, using means, medians, and geometric means. Other common second-moment statistics in studies are standard deviations and extreme values (such as the minimum and maximum). For consistency, the concentrations are converted to medians when they are reported differently. The GSD of the concentrations is estimated assuming that concentrations are log-normally distributed.^94−97^

**Figure 1:**
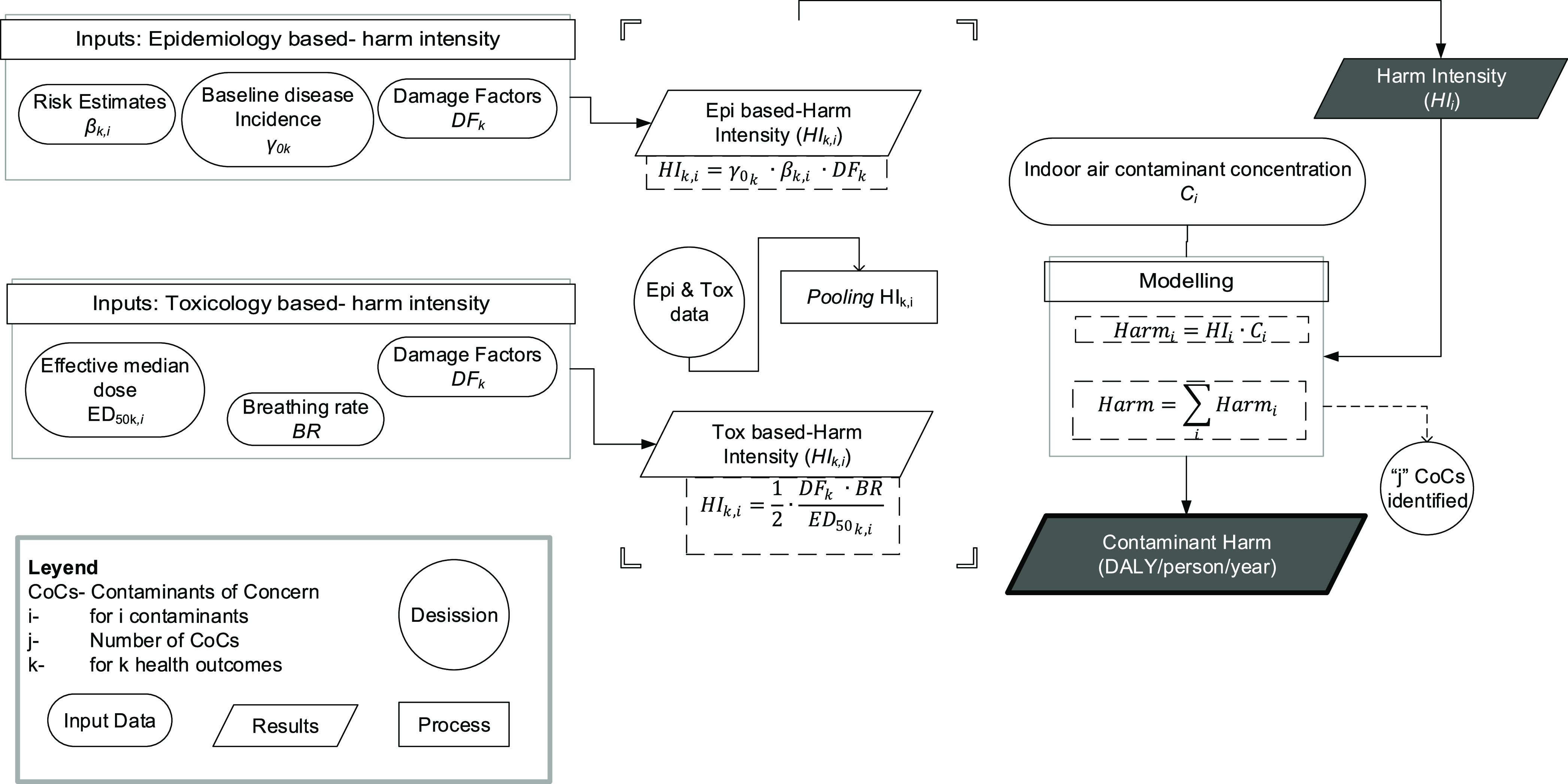
Harm approach flow diagram.

We use standard statistical approaches to pool log–normal distributions,^98−103^ and when data for a single contaminant is obtained from each approach, their respective values are combined to produce a single-point estimate and uncertainty. Contaminant concentrations from different references are combined similarly. A simplified flowchart illustrating the process from HI analysis to contaminant harm is provided in Figure 1. Further details about the model are given in the Supporting Information.

## 3 Results

### 3.1 Harm Intensity

The latest epidemiological and toxicological research is used to calculate the HI of 44 common indoor air contaminants found in dwellings, and the HI of the coarse fraction was calculated by subtracting the fine fraction from PM_10_. Five contaminants were found in both toxicological and epidemiological studies: acrolein (C_3_H_4_O), benzene (C_6_H_6_), formaldehyde (HCHO), ozone (O_3_), and radon (Rn). Therefore, their HIs are pooled by using both data sources. Table 1 shows the single-point estimates and uncertainties.

**Table 1:** Harm Intensities

contaminant	**median**^a^	GSD	approach
acetaldehyde	0.053	4.8	toxicology
acrolein	1.2	4.0	pooled
acrylonitrile	1.2	4.1	toxicology
benzene	0.067	1.4	pooled
benzyl chloride	0.062	11	toxicology
1,3-butadiene	0.27	3.9	toxicology
2-butoxyethanol	0.010	8.7	toxicology
cadmium Cd(II)	5.3	8.9	toxicology
carbon disulfide	0.29	1.1	toxicology
carbon tetrachloride	0.52	7.3	toxicology
chloromethane	0.00027	10	toxicology
chromium Cr(VI)	17	15	toxicology
crotonaldehyde(trans)	1.1	7.2	toxicology
1,2-dibromoethane	3.4	5.8	toxicology
1,4-dichlorobenzene	0.012	6.4	toxicology
1,2-dichloroethane	0.052	5.4	toxicology
1,1-dichloroethene	0.15	6.1	toxicology
ethanol	0.0005	5.8	toxicology
2-ethylhexanol	0.0029	8.4	toxicology
formaldehyde	4.3	2.0	pooled
hexachlorobutadiene	0.030	4.8	toxicology
hexane	0.0018	8.7	toxicology
isoprene	0.0092	7.0	toxicology
limonene (d-···)	0.0093	6.5	toxicology
2-methoxyethanol	0.0028	7.8	toxicology
methyl methacrylate	0.051	2.8	toxicology
methyl *tert*-butyl ether	0.026	4.6	toxicology
methylene chloride	0.010	5.6	toxicology
mold	0.026^b^	2.1	epidemiology
naphthalene	0.36	5.9	toxicology
nitrogen dioxide	5.7	1.7	epidemiology
ozone	1.3	1.9	pooled
PM_10_	30	1.3	epidemiology
PM_10__–__2.5_	3.8	4.3	
PM_2.5_	60	1.2	epidemiology
radon	0.44^c^	1.6	pooled
styrene	0.11	4.7	toxicology
sulfur dioxide	1.3	5.3	epidemiology
1,1,2,2-tetrachloroethane	0.13	6.2	toxicology
tetrachloroethene	0.052	6.2	toxicology
toluene	0.00087	5.4	toxicology
1,1,2-trichloroethane	0.15	5.7	toxicology
trichloroethylene	0.0035	5.1	toxicology
vinyl chloride	0.98	5.4	toxicology
xylenes	0.0034	6.1	toxicology

a DALY/μg/m^3^/10^5^ person/year.

b DALY/cfu/m^3^/10^5^ person/year; cfu, colony-forming units.

c DALY/Bq/m^3^/10^5^ person/year; Bq, Becquerels.

PM_2.5_ shows the greatest HI, but PM_10_ and chromium are also important because they have HIs that are several times higher than those of any other of the included contaminants. The elevated HI observed for PM results from the combined effects of baseline incidence, relative risk, and DFs, all of which relate to all-cause mortality associated with particle exposure. Chromium’s high magnitude of HI is a function of its toxicological characteristics, specifically the low-effective median dose, that induces an effect in the population.

The HIs derived from toxicology- and epidemiology-based approaches are not dependent on specific concentration values. In the toxicology-based approach, the ED50 (ED for 50% of the population) encompasses the dose, including the exposure itself. Likewise, in the epidemiology-based approach, the risk coefficient derived from exposure concentrations implicitly incorporates the exposure. This inherent feature of the harm metric enables its broad application across different environments.

HIs alone do not give a complete understanding of the potential harm a contaminant can cause in a space and neither do concentrations. Concentrations and HIs are required together. It is important to note that a low concentration of a contaminant with a high HI could pose a higher health risk than a high concentration of a contaminant with a low HI.

### 3.2 Representative Concentrations in Dwellings (C_*i*_)

A total of 145 unique references were analyzed, which comprised 827 data sets containing concentrations for the 44 airborne contaminants included in the review. The value of PM_10–2.5_ was derived from these data sets. The references cover a time period between 2000 and 2020. The data come from 31 different countries, as well as from regional reviews, such as Africa,^104^ Europe,^105^ and other grouped countries.^106^ The countries with the highest number of samples were the United States of America, China, Canada, and United Kingdom (Global North countries). Some countries did not appear in our review; further details can be found in the Supporting Information.

Table 2 presents the medians, uncertainty in the representative concentrations (after modeling their distributions), and the number of data sets reviewed for each contaminant. Contaminant concentrations are reported in micrograms per cubic meter, except for radon (Bq/m^3^) and mold spores (cfu/m^3^). The five most abundant contaminants by mass are ethanol, PM_10_, formaldehyde, PM_2.5_, and nitrogen dioxide (NO_2_). PM_10–2.5_ is within this group but not mentioned because it was inferred from the other PM fractions. Median representative concentrations for ethanol, PM_10_, and formaldehyde are 110 μg/m^3^ (7 data sets), 62 μg/m^3^ (48 data sets), and 28 μg/m^3^ (67 data sets), respectively. Twenty-eight contaminants have a median concentration of <2.0 μg/m^3^.

**Table 2:** Representative Concentrations

contaminant	**median**^a^	GSD	data sets
acetaldehyde	13	1.7	36
acrolein	0.60	1.5	20
acrylonitrile	0.71	1.2	4
benzene	2.2	1.3	65
benzyl chloride	0.22	3.4	2
1,3-butadiene	0.43	1.5	11
2-butoxyethanol	2.7	1.5	8
cadmium Cd(II)	0.011	2.2	5
carbon disulfide	0.31	1.6	2
carbon tetrachloride	0.50	1.3	18
chloromethane	1.6	1.1	2
chromium Cr(VI)	0.0031	3.2	2
crotonaldehyde(trans)	0.65	1.9	13
1,2-dibromoethane	0.018	6.0	3
1,4-dichlorobenzene	1.90	1.7	30
1,2-dichloroethane	0.52	1.3	21
1,1-dichloroethene	0.48	1.5	3
ethanol	110	1.6	7
2-ethylhexanol	1.7	1.7	6
formaldehyde	28	1.2	67
hexachlorobutadiene	1.3	2.2	2
hexane	1.4	1.7	19
isoprene	6.0	1.5	8
limonene (d-···)	12	1.9	39
2-methoxyethanol	0.021	12	4
methyl methacrylate	0.082	4.3	2
methyl *tert*-butyl ether	3.3	2.1	8
methylene chloride	0.67	2.1	6
mold	160^b^	1.3	9
naphthalene	1.1	2.2	19
nitrogen dioxide	22	1.3	48
ozone	7.3	2.2	10
PM_10_	62	1.3	35
PM_10__–__2.5_	35	1.4	
PM_2.5_	26	1.3	107
radon	78^c^	1.4	10
styrene	1.6	1.3	34
sulfur dioxide	0.41	4.0	8
1,1,2,2-tetrachloroethane	0.040	3.4	4
tetrachloroethene	0.83	1.1	21
toluene	13	1.1	67
1,1,2-trichloroethane	0.28	1.4	9
trichloroethylene	0.45	1.1	20
vinyl chloride	0.072	3.3	2
xylenes	6.8	1.3	13

a μg/m^3^.

b cfu/m^3^; cfu, colony-forming units.

c Bq/m^3^; Bq, Becquerels.

The contaminant concentration distributions in Table 2 reflect typical exposures caused by activities expected to occur in homes, which might include cooking, candle use, smoking, combustion of solid fuels (wood and coal), and incense burning; see the Supporting Information for a discussion.

### 3.3 Contaminants of Concern

Table 3 gives the estimated chronic harm (DALYs/10^5^ person/year) from exposure to the 45 contaminants in descending order. PM_2.5_, PM_10–2.5_, nitrogen dioxide, formaldehyde, radon, and ozone are ranked highest with an estimated median DALYs/10^5^ person/year of 1600 (GSD 1.3), 130 (GSD 4.5), 120 (GSD 1.8), 120 (GSD 2.0), 34 (GSD 1.8), and 10 (GSD 2.7), respectively, higher than all other contaminants by at least 1 order of magnitude.

**Table 3:** Contaminant Harm

contaminant	**median**^a^	GSD
PM_10_	1900	1.4
PM_2.5_	1600	1.3
PM_10__–__2.5_	130	4.5
nitrogen dioxide	120	1.8
formaldehyde	120	2.0
radon	34	1.8
ozone	10	2.7
mold	4.0	2.3
acrolein	0.73	4.1
acrylonitrile	0.73	4.3
acetaldehyde	0.68	5.1
crotonaldehyde(trans)	0.59	8.0
sulfur dioxide	0.56	8.1
naphthalene	0.33	6.4
styrene	0.21	4.8
carbon tetrachloride	0.19	6.5
benzene	0.15	1.6
methyl *tert*-butyl ether	0.11	5.6
limonene (d-···)	0.11	7.5
1,3-butadiene	0.10	4.0
1,1-dichloroethene	0.10	5.7
carbon disulfide	0.089	1.6
vinyl chloride	0.070	7.6
ethanol	0.068	6.2
1,2-dibromoethane	0.062	10
isoprene	0.061	7.1
cadmium Cd(II)	0.058	9.1
1,1,2-trichloroethane	0.056	5.9
hexachlorobutadiene	0.054	5.6
chromium Cr(VI)	0.045	11
tetrachloroethene	0.044	5.7
1,2-dichloroethane	0.030	5.2
1,4-dichlorobenzene	0.024	6.2
xylenes	0.018	6.2
toluene	0.013	5.2
2-butoxyethanol	0.0098	7.2
1,1,2,2-tetrachloroethane	0.0083	8.8
benzyl chloride	0.0075	11
methylene chloride	0.0061	6.2
2-ethylhexanol	0.0048	7.9
methyl methacrylate	0.0042	6.5
trichloroethylene	0.0018	5.1
hexane	0.0017	9.8
chloromethane	0.0010	9.2
2-methoxyethanol	0.000060	21

a DALY/10^5^ person/year.

**Figure 2:**
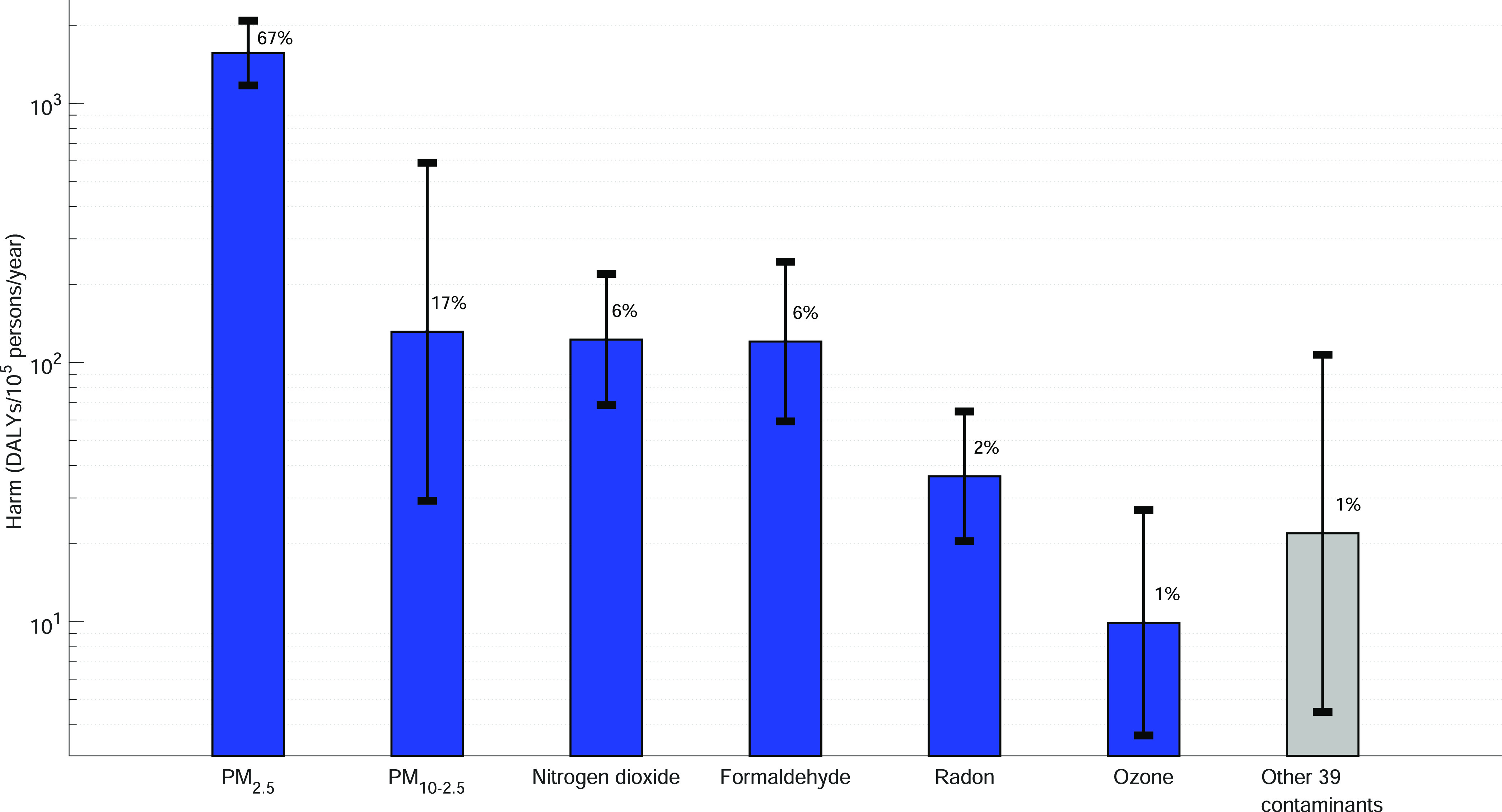
Harm caused by CoCs. Median (bar) and GSD (error bar). Percentage contribution for total harm.

Summing the harm for all contaminants at their representative concentrations gives a total median harm of 2200 DALYs/10^5^ person/year (GSD 1.6). PM_2.5_, PM_10–2.5_, formaldehyde, nitrogen dioxide, radon, and ozone account for 99.5% of total harm caused by typical indoor air contaminants. Therefore, they should be considered CoCs for dwellings (see Figure 2).

## 4 Discussion

### 4.1 Concentrations of Airborne Contaminants in Dwellings

**Figure 3:**
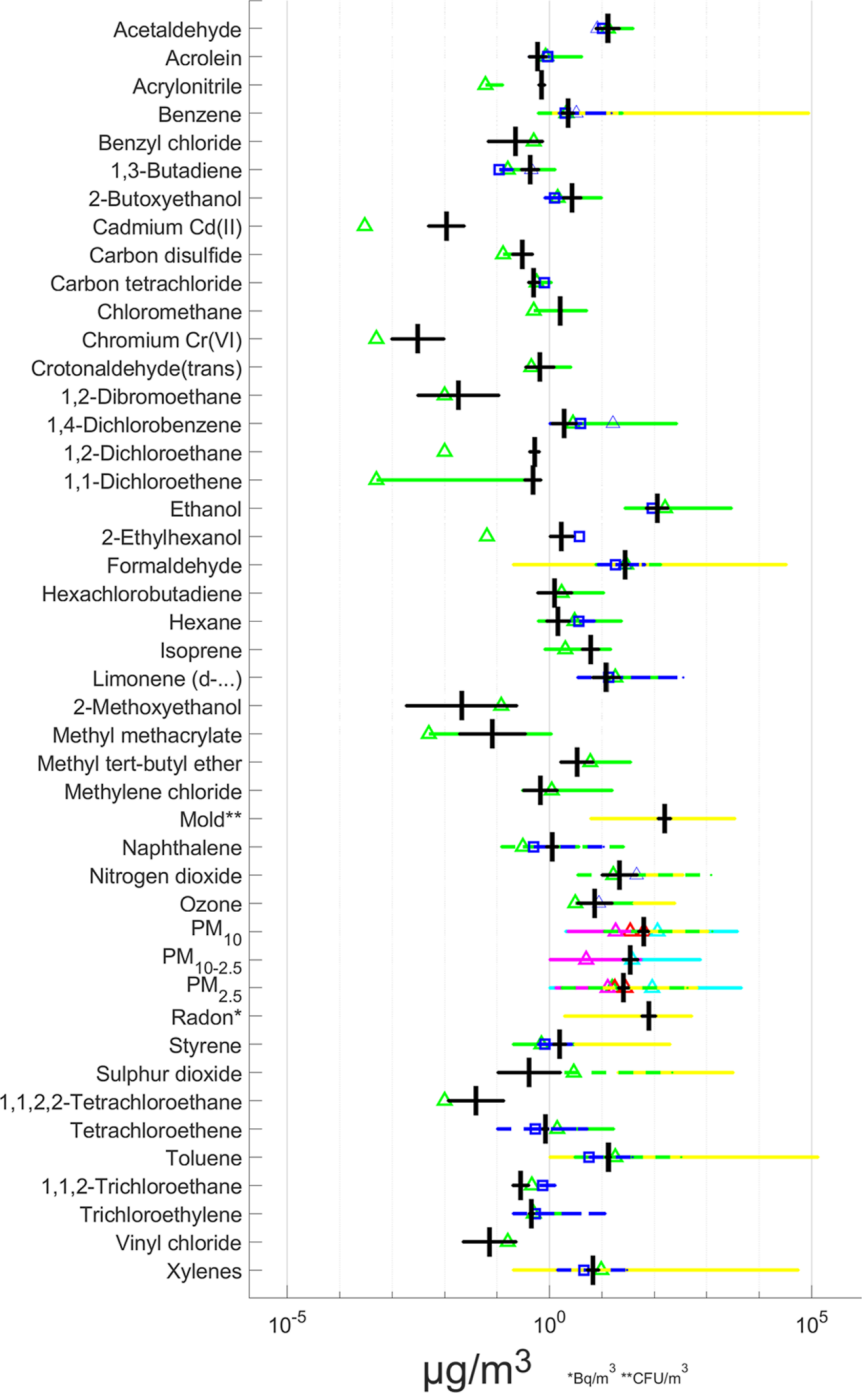
Representative airborne contaminant concentrations. Evaluation was performed against prior research. Median with GSD. Black, current work; green-triangle = Logue et al.,^18^ blue-triangle = Fazli et al.,^19^ red-triangle = Morawska et al.,^12,107^ cyan-triangle = Ilacqua et al.,^111^ magenta-triangle = Nishihama et al.,^109^ yellow-triangle = Ye et al.,^108^ green-square, dashes = Vardoulakis et al.,^84^ blue-square, and dashes = Halios et al.^110^

Our concentrations of the 45 contaminants are broadly similar to those reported in other literature reviews. Here, we compare our meta-analyses with estimates from nine earlier studies.^12,19,83,84,107−111^Figure 3 illustrates their trends over the past 3 decades. There are some noticeable differences in the medians and in the overlaps of the GSD, but generally, there is good agreement between our results (in black) and the other studies. Differences may be attributed to the inherent variations in the individual studies; for further details see the Supporting Information. The similarities in concentrations may be attributed to the fact that our review, and the previous studies, primarily rely on data from a limited number of countries, including the United States of America, China, Canada, and the United Kingdom, predominantly high-income industrialized nations that often refer to Global North countries; see Section 4.5 for the implications of these results.

Our estimation of PM_10–2.5_ concentrations, determined by subtracting PM_2.5_ from PM_10_, introduces some uncertainty in interpreting the coarse fraction. However, our central tendency metric aligns well with the findings of Ilacqua et al.,^111^ who compiled measurements of PM_10–2.5_ from various studies. We observe that the PM_10–2.5_ size fraction in dwellings is still under-reported in the literature, and it is common practice to derive this contaminant by subtracting PM_2.5_ from PM_10_.^87,112^ We estimate that the fraction of PM_10_ attributed to PM_10–2.5_ is 0.36, which is comparable to a value of 0.56 determined by *in situ* measurements in dwellings by Morawska et al.,^107^ 0.46 by Ilacqua et al.,^111^ 0.26 by Nishihama et al.,^109^ and 0.19 by Morawska et al.^12^

### 4.2 Chronic Harm in Dwellings

CoCs are identified in Section 3.3 that account for over 99% of total median harm caused by indoor air contaminants. Our results of harm caused by these chemicals is compared against those in existing publications: our estimate of harm from PM_2.5_ is 3 times higher than that of Logue et al.^18^ and 1 order of magnitude higher than that of Fazli and Stephens.^19^ This is because (i) our representative concentrations are higher, and (ii) we sourced a higher risk estimate that indicates PM_2.5_ is more harmful than what is previously thought. These differences may be attributable to the lower indoor PM_2.5_ concentrations and the time-weighted concentrations used in American homes by Logue et al.;^18^ see the Supporting Information for a discussion.

The estimated harm caused by PM_10–2.5_ is a novel topic, and to the best of our knowledge, no previous estimates have been made using DALYs. The estimated harm from nitrogen dioxide is higher than that estimated by Logue et al.^18^ and Fazli and Stephens^19^ because they use lower risk and damage estimates solely linked to hospital admissions, whereas we use the broader measure of all-cause mortality. The estimated harm from formaldehyde is higher than that estimated by Huijbregts et al.^15^ because we account for the effects of three health outcomes, whereas they only consider carcinogenic effects (leukemia). The estimated harm from radon is within the same order of magnitude as the global burden of disease attributable to radon in dwellings in 2019.^8^ Finally, the estimated harm for ozone is marginally higher that those by others^18,19^ found using similar risk estimates and concentrations.

The contaminants we identify that pose the highest harm, PM_10_, PM_2.5_, formaldehyde, and nitrogen dioxide, are extensively studied; see Table 2. Ethanol is the most abundant species in dwellings, but its contribution to harm is small. Unlike previous analyses that relied on visual mold presence,^113^ our assessment of mold burden incorporates the measured concentration of Cladosporium mold spores; see also the Supporting Information. To assess the validity of our estimates of harm and to contextualize them, we compare them with independent estimates of chronic health impacts (in DALYs) from the inhalation of airborne contaminants in dwellings. Three studies conducted in the United States of America^18,19,24^ applied the IND-DALY and/or ID-DALY methods, and three global/European studies^12,113,114^ followed a comparative risk assessment approach using the population attributable fraction. A more detailed comparison can be found in Supporting Figure A.

**Figure 4:**
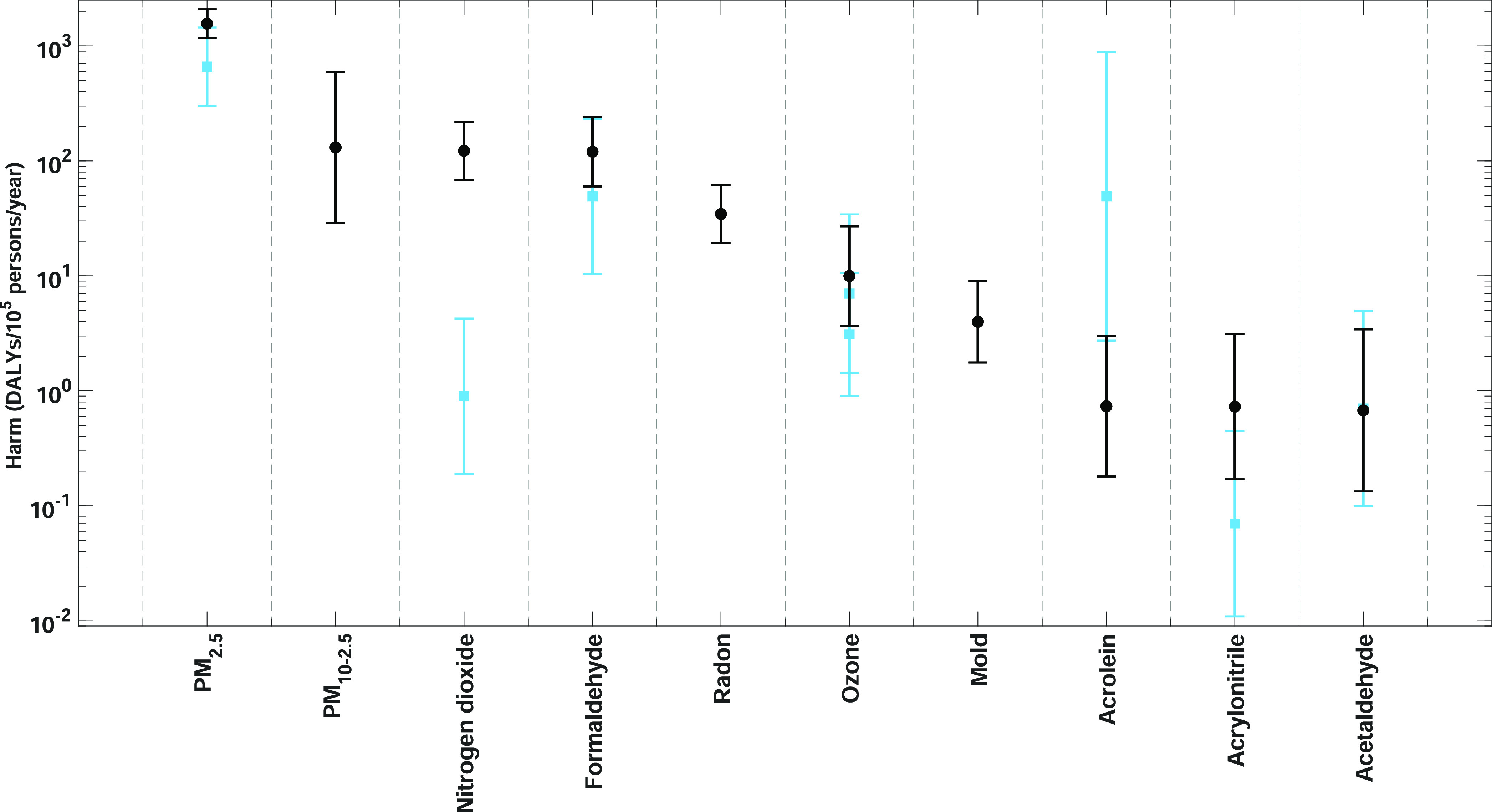
Comparing chronic harm for the 10 most harmful contaminants against Logue et al.^18^ Median with GSD. Black, current work; blue, Logue et al.

This paper seeks to advance and augment Logue’s IND and ID approaches, and so we compare our estimate against theirs in Figure 4. There are several differences. The first is that we analyze three additional contaminants (PM_10–2.5_, radon, and mold), and we have expanded the IND approach to include four contaminants (acrolein, benzene, formaldehyde, and radon) because of the growing number of epidemiology-based studies focusing on their health impacts in recent years. The similarities in Figure 4 suggest that the two models are converging toward a similar conclusion, and this is perhaps reassuring given the assumption of a linear concentration–response relationship at low concentrations. However, it is also evident that while the harm estimates for some contaminants remain relatively consistent, there are noticeable changes in the harm estimate for others, such as acrolein. Our harm estimates have reduced uncertainty by using the most up to date health data, including current GBD DFs,^8^ dedicated uncertainty studies;^115^ see the Supporting Information for further details.

There are a number of factors that contribute to the overall variability observed by the different references, including the choice of CRF, the use of different health outcomes, reporting various central tendency metrics, spatial and population resolution variations, the geographic scope covered, differences in concentration estimates, and variations in the methodological frameworks they followed.

We find that some contaminants pose a higher or lower level of harm than those previously estimated. This change is not solely attributed to our methodology because it is similar to those followed in previous studies of harm in dwellings.^8,12,18,19,24,114^ Formaldehyde, radon, and ozone have data from both epidemiology and toxicology studies. However, PM_2.5_, PM_10–2.5_, and nitrogen dioxide are characterized only by epidemiological data. This highlights a key need for additional toxicological research into these pollutants to improve our understanding of their health effects and provide a more comprehensive and robust estimate of the harm they cause.

Our analysis of harm caused by the coarse fraction suggests that chronic exposure to it has a considerable impact on health.^87,112^ Nevertheless, the analysis also shows that PM_2.5_ contributes more to the health burden.

The total harm for all 44 independent indoor airborne contaminants has a median value of 2200 DALYs/10^5^ person/year (GSD 1.6). This is roughly 5 times higher than the global burden of disease from secondhand smoke in dwellings^12^ and double the global burden of disease from PM_2.5_ household air pollution.^14^ In comparison, the 2019 GBD study estimated that the all-cause morbidity and mortality burden was 33,000 DALYs/10^5^ person/year (GSD 1.1). The 2200 DALYs/10^5^ person/year is around 7% of this total GBD. There are no studies that can be used to directly check the plausibility of this value. Therefore, we use the GBD study that estimates that the household air pollution from PM_2.5_ (from solid fuels) is 1200 DALYs/10^5^ person/year. Our study suggests that PM_2.5_ accounts for 65% of the total burden. By applying this fraction to the GBD data and extrapolating it, it is possible to estimate that the household air pollution from all contaminants might be around 1850 DALYs/10^5^ person/year and 5.5% of the total GBD. This is approximately similar to our value of 7% and provides some reassurance of its plausibility.

The HI metric is derived from toxicology and epidemiology health research for chronic impacts at a population scale and is normalized by a concentration. Therefore, this metric can be used to assess the harm from the inhalation of airborne contaminants in any scenario where the assumption of a linear CRF holds (see the Supporting Information for further discussion), which makes it appropriate for most building types. Further improvements in the quality of the health data may enhance our estimates of the HI, but it is not expected to vary with activity, region, or building type. This makes it a universal metric that is unaffected by interventions. Conversely, contaminant concentrations are affected by these factors and so are affected by interventions.

### 4.3 Contaminant Ranking and Prioritization

The DALY metric allows contaminants to be ranked by the harm they cause and then prioritized. Other studies that ranked and prioritized airborne contaminants in dwellings used different qualitative or quantitative methods. For example, Halios et al.^110^ identified a subset of high-priority VOCs based on their adverse-effect end points and the number of studies reporting their concentrations. The VOCs they prioritized were the following: trichloroethylene, tetrachloroethylene, 2-methylbutane, tetrachlorocarbon, benzene, ethylbenzene, m + *p*-xylene, o-xylene, styrene, toluene, trimethylbenzene, acetone, acetaldehyde, formaldehyde, naphthalene, α – pinene, and limonene. Sarigiannis et al.^105^ used a combination of quantitative risk characterization metrics to prioritize 10 major organic compounds and highlight benzene as the indoor contaminant of major concern, followed by formaldehyde, toluene, and xylenes. Azuma et al.^116^ ranked acrolein, nitrogen dioxide, and benzene as the highest risk pollutants (from a list of 49 indoor contaminants) because they resulted in a margin of exposure of less than 1 (a lower magnitude of this metric indicates higher health risks). These studies prioritized contaminants by interpreting risk using predefined thresholds or chosen rules, whereas we applied the DALY metric. Furthermore, they all follow a different prioritization method, whereas DALY provides a quantitative number that allows a direct comparison between contaminants.

We agree with the three studies^105,110,116^ that formaldehyde and nitrogen dioxide are CoCs. One study highlights that acrolein is important, but our analysis uses more up-to-date toxicology and epidemiology data and finds that it is less important than what is previously thought. The three references agree that benzene is a priority contaminant because it is highly carcinogenic in humans. Our HI for benzene considers this too, and when carcinogenic health effects are considered as DALYs, their contribution to the total harm is small when compared to the other contaminants (ranked the 17th most harmful contaminant in Table 3). This indicates that the estimated health burden from benzene is minor at the concentrations identified in Section 3.2, and so, at a societal level, the presence of benzene is not a substantial component of the total harm from exposure to indoor air. It may be necessary to regulate the sources of carcinogens (35 of the 45 contaminants are carcinogens) or their concentrations in air via IAQ standards if they are expected to be high.

The CoCs in dwellings, PM_2.5_, PM_10_, nitrogen dioxide, formaldehyde, ozone, and radon, each contribute 67, 17, 6, 6, 2, and 1% to the median total harm, respectively. This shows that it is possible to ensure acceptable air quality in a dwelling by regulating only a few contaminants. This finding is important for building professionals and regulatory bodies.

### 4.4 Acceptable Indoor Air Quality

The CoCs identified in Section 3.3 can be used with a reference scenario to regulate IAQ in dwellings by determining a *harm budget*. A reference scenario is a specific set of buildings that all comply with a recognized IAQ standard, and so the IAQ in those dwellings might be logically assumed to be *acceptable*. The median total harm is then used to set the budget and determine acceptable IAQ (AIAQ) quantitatively. A framework and example is given in Appendix A.

### 4.5 Limitations and Future Developments

We derive estimates of harm as the product of HI and representative concentrations of airborne contaminants found in dwellings. This simple and straightforward procedure assesses the chronic health impacts associated with indoor exposure to airborne contaminants. However, our study has limitations.

Variations in demographics, regions, and indoor behaviors influence exposure concentrations. The aim of this study is to quantify overall population impacts; therefore, we did not model these directly. Instead they are captured implicitly in the epidemiology data’s confidence intervals. Long-term ambient air cohorts encompass a wide range of exposure concentrations, and the pooling of these studies increases that variation further. Such exposure differences affect the confidence intervals of epidemiological metrics such as risk estimates. Furthermore, other studies have attempted to address this by adjusting parameters or considering lower exposure percentages based on the time spent in different locations, but there is currently no consensus in the literature on a standard approach. Our pooled total harm values align with the findings from studies conducting subpopulation analyses. Therefore, we do not expect significant changes in the CoCs or the ranking of contaminants based on demographic aspects. Ventilation and IAQ standards do not publish recommendations for specific population characteristics such as being hypersensitive to certain contaminants, suggesting their limited relevance to our analysis. A more detailed stratified analysis falls outside the scope of this study but would provide further insight and should be the subject of future research.

Our results inform the understanding of acceptable IAQ and its regulation by ventilation and IAQ standards, such as ASHRAE 62.2. The harm budget has been developed using ASHRAE 62.2, and so it is important to acknowledge that the work is generally focused on the ability of ventilation to mitigate against exposure to airborne contaminants.

We reviewed 145 references with 827 data sets on indoor airborne contaminant measurements, and although this is substantial, some countries are under-represented. Consequently, the data may not accurately reflect the concentrations of contaminants in smaller countries or regions due to a scarcity of studies, potentially leading to lifestyle-based discrepancies. Thus, caution is advised when comparing these generalized results to those of contaminants in a specific location. Additionally, there is still significant uncertainty in the concentrations of some contaminants; therefore, further field work is required to reduce this. For example, Table 2 shows that there are 12 contaminants with <5 data sources.

It is important to conduct a more detailed analysis that accounts for factors that may affect population exposure to indoor air contaminants by country or region, climate, and building characteristics. Our approach is general, but when evaluating local populations, the use of local data gives a more accurate understanding of the harm to that population. Furthermore, considering local populations might highlight the presence of other CoCs in those scenarios.

Future work should include a hazard assessment that compares the harm identified in Section 4.2 to the harm associated with complying with the contaminant concentrations given in the existing IAQ standards and guidelines. This would show the relative protectiveness that these standards provide to the occupants of the buildings they regulate. This analysis would provide valuable insights into whether the harm caused by the inhalation of airborne contaminants in dwellings aligns with the acceptable levels set by regulators and the wider public. A comparison will contribute to a better understanding of the potential health risks and the importance of adhering to standards and guidelines.

In our study, we focused on a limited list of 45 contaminants commonly found in dwellings, selected based on their known harmful effects and availability of data. However, an important limitation is the omission of emerging contaminants like PM_1_ and ultrafine particles PM_0.1_, fungicides and pesticides, flame retardants, and endocrine disruptors such as phthalates, which have gained increasing attention in research.^13,117−119^ These substances have the potential to significantly contribute to harm in indoor environments. Therefore, future studies should expand the scope to include these emerging contaminants, enabling a more comprehensive assessment of harm. Our list contains several semi-VOCs (1,4-dichlorobenzene, hexachlorobutadiene, and naphthalene). It may not be so important to consider semi-VOCs in future work for IAQ standards—not because they are unimportant—but because they are not always removed by ventilation. Increasing ventilation has only a small impact on their airborne concentration because their net emission generally increases as their airborne concentration decreases.^120,121^ This makes estimating exposure to them complicated,^122^ and so the mitigation solution is source control rather than ventilation.

The analysis assumes PM equitoxicity, where all particles are equally toxic per unit mass inhaled. Emerging evidence suggests that health effects can vary by PM composition,^87^ but more studies on the health impacts of PM from different sources are needed before it is possible to determine exposureresponse relationships.^123^ Differentiating between indoor and outdoor PM HI requires separate chronic risk estimates for each location, which are unavailable from current exposure surrogates or from indoor concentrations alone.^124^ For perspective, the indoor PM_2.5_ HI would need to be around 13 times lower to be equal to that of nitrogen dioxide and would need to be 2200 times lower to be equal to that of acrolein, when it would cease being a contaminant of concern. Overall, PM size remains the most robust predictor of long-term exposure incidence.

There may be synergistic effects for some combinations of contaminants. These are, however, complex, and there is also limited evidence for them. They also do not occur at the concentrations found in dwellings. Multipollutant effects are rarely used in epidemiological exposure assessments. When evaluating a population of buildings, independent concentration distributions are used for each contaminant, and so it is not possible to identify interactions. It may be possible to do this for specific buildings, but the analysis would be uncertain. Therefore, we assume that all harm is additive and acknowledge that there is a possibility that harm may be under or overestimated in some circumstances and that there is currently no limit value where the additive total harm principle becomes invalid, but it might be identified in the future.^48^

Both the epidemiological and toxicological data that underpin the HIs and the concentrations are linked to chronic effects and exposures, and so, it is not possible to consider acute health effects with them. For some of the contaminants, such as the reactive oxidizing species, including ozone and nitrogen dioxide, acute impacts from elevated short-term exposures may be more important than the chronic harm we calculate.

The HIs are appropriate for low to moderate concentrations, where a CRF is expected to be approximately linear, and extrapolations to higher concentrations yield unreasonably high estimates of harm. We expand on this limitation and on the expected shapes of C–R curves in the Supporting Information and Supporting Figure B.

A harm budget can be used to determine AIAQ in buildings (see Appendix A), but before it can be implemented in a standard, several key factors should be considered. First, it may be better to limit the CoCs to two or three of the most harmful to make source control, remediation, diagnostics, and enforcement simpler. Second, it would be useful to consider the harm budget in relative terms using a dimensionless magnitude instead of using absolute terms.

Finally, the study focuses on dwellings, but the concept of the HI extends to any other environment, where a linear C–R is expected. Future work will explore this in a range of settings.

Despite these limitations, this study provides a comprehensive estimate of the total harm from indoor air contaminants using representative indoor concentration data of Global North countries and globally derived epidemiological and toxicological data lacking geographic specificity. The results presented herein can be used to inform appropriate remediation by showing where the greatest reduction in harm can be achieved. Cost-benefit analyses could be used to show the interventions that give the greatest harm reductions for the least capital outlay. Furthermore, the HIs can be used to assess the harm from airborne contaminants measured in field surveys or predicted by models.

In summary, we present the HI metric derived from epidemiology and toxicology research that expresses the harm per unit concentration of contaminants. Representative concentrations of 45 airborne contaminants commonly found in dwellings and their HIs are used to estimate contaminant harm as DALY/person/year. We find that the CoCs in dwellings are PM_2.5_, PM_10–2.5_, nitrogen dioxide, formaldehyde, radon, and ozone, which account for 99.5% of the total median harm. Finally, we conceive a *harm budget* as a way to quantitatively determine *acceptable* IAQ based on exposure to airborne contaminants in buildings.

## Appendix A

### Framework for a Harm Budget

The harm attributable to chronic exposures is calculated using eq 4 from the representative indoor concentrations and the HIs. The values of harm are used to rank the contaminants and identify CoC; see Section 3.3. These CoCs can then be used to regulate IAQ in dwellings. One way of doing this is to set a *harm budget*, the distribution of harm that is expected in an acceptable reference scenario. An extensive analysis of different approaches to set a harm budget is beyond the scope of this paper, so here we use one method as a demonstration of the process.

A *reference scenario* is a specific set of dwellings that all comply with a recognized IAQ standard^125−128^ and so the IAQ in those dwellings might be logically assumed to be *acceptable*. A case study is required where the CoCs identified in Section 3.3 are measured. Therefore, we use a cohort of dwellings that already conform to an existing ventilation and IAQ standard.^36^ The cohort of Singer et al.^125^ comprises 70 Californian homes that comply with the mechanical ventilation requirements of California’s building energy efficiency standards (CalEnergy Code).^129^ This sample may not be as large as is desirable, but a cohort with high statistical power where all dwellings comply with an IAQ standard does not exist. This is the best available. The contaminant concentrations in these homes reflect AIAQ as determined by the current CalEnergy Code. It is used as a reference for the median concentrations of the CoCs, and the harm budget is calculated by multiplying each of them by their individual HIs. This is described generally by combining eqs 1 and 2.

9

Here, *N*_CoC_ is the number of CoCs and *C̅* is the median concentration for a scenario. The median concentration aligns with ANSI/ASHRAE 62.2, defining requirements based on typical buildings without predetermining allowable exceedances. It shifts distributions toward lower harm for more homes versus loosening protection with an upper anchor. The median offers balanced health protection, reflecting current performance.

#### A.1 Results

To quantitatively define AIAQ, reference concentrations are required for the CoCs. A study of 70 Californian homes is used as a reference for median PM_2.5_, formaldehyde, and nitrogen dioxide concentrations at 5, 23, and 9 μg/m^3^, respectively.^125^ Ozone and radon were not measured in these dwellings, and so guideline values of 40 μg/m^3^ and 100 Bq/m^3^ are used as reference concentrations, respectively.^130^ Furthermore, they are likely to contribute only a small proportion of the total harm. PM_10–2.5_ is not considered here because a guideline value does not exist. A PM_10–2.5_ threshold could be inferred, but we want to illustrate the flexibility of the harm budget approach instead. We acknowledge that this is an imperfect compromise.

All of the homes comply with the mechanical ventilation requirements of California’s building energy efficiency standards (CalEnergy Code).^129^ Therefore, the contaminant concentrations in these homes can be thought to reflect the harm caused by air quality considered *acceptable* by the current CalEnergy Code.

Homes complying with ASHRAE 62.2 in California exhibit a harm distribution with a median of 600 DALYs/10^5^ person/year (GSD 1.2), rounded to one significant figure. In this context, using one significant figure makes sense because the reference concentrations are expected to vary. Consequently, it will cause the output to fluctuate, ultimately leading to convergence of around the same order of magnitude. Since these dwellings meet the existing ventilation standard, their central tendency harm logically represents an acceptable IAQ benchmark. The median harm in 62.2-compliant homes therefore anchors the proposed budget, aligning new standards with current regulatory frameworks. Contaminant harm from the typical median concentrations for dwellings given in Table 2 exceeds this budget by just under 4 times.

Our use of the harm budget approach in this study is a demonstration, and the analysis is not exhaustive. We refrain from concluding whether this value aligns with a universally accepted harm budget as that determination awaits further research. Concentrations and associated harm levels in other ASHRAE 62.2-compliant dwellings may vary. Generalizing our findings to all such dwellings is not possible, but they serve as a starting point for harm budget evaluation rather than a definitive solution. Changes in the budget’s magnitude will result from comparisons with other non-IAQ hazards and evaluations of additional houses.

## Supporting Information Available

Harm; evaluation against prior research; concentration–harm curves; forest plots for meta-analysis of PM_2.5_, subgroup by country or region; and forest plots for meta-analysis of PM_10_, subgroup by country or region (PDF)

## References

[ref1] WHO. WHO Global Air Quality Guidelines: Particulate Matter (PM2. 5 and PM10), Ozone, Nitrogen Dioxide, Sulfur Dioxide and Carbon Monoxide; World Health Organization, 2021. 34662007

[ref2] HEI, IHME. State of Global Air 2020: A Global Report Card on Air Pollution Exposures and Their Impacts on Human Health; Institute for Health Metrics and Evaluation, and Health Effects Institute, 2020. available at: https://www.stateofglobalair.org (accessed 10 10, 2022).

[ref3] Klepeis N. E.; Nelson W. C.; Ott W. R.; Robinson J. P.; Tsang A. M.; Switzer P.; Behar J. V.; Hern S. C.; Engelmann W. H. The national human activity pattern survey (nhaps): a resource for assessing exposure to environmental pollutants. J. Exposure Sci. Environ. Epidemiol. 2001, 11 (3), 231–252. 10.1038/sj.jea.7500165. 11477521

[ref4] E.-E. Commission. Indoor Air Pollution: New Eu Research Reveals Higher Risks than Previously thought; Joint Research Center, 2003;.

[ref5] U. E. P. Agency. Report to Congress on Indoor Air Quality, Volume ii: Assessment and Control of Indoor Air Pollution, Technical Report EPA/400/1–89/001C, 1989;.

[ref6] Murray C. J. Quantifying the burden of disease: the technical basis for disability-adjusted life years. Bull. World Health Organ. 1994, 72 (3), 429. 8062401 PMC2486718

[ref7] Wang N.; Phelan P. E.; Gonzalez J.; Harris C.; Henze G. P.; Hutchinson R.; Langevin J.; Lazarus M. A.; Nelson B.; Pyke C.; Roth K.; Rouse D.; Sawyer K.; Selkowitz S. Ten questions concerning future buildings beyond zero energy and carbon neutrality. Build. Environ. 2017, 119, 169–182. 10.1016/j.buildenv.2017.04.006.

[ref8] IHME Institute for health metrics and evaluation tool, 2022. www.vizhub.healthdata.org/gbd-results/ (accessed 02 31, 2023).

[ref9] Lee K. K.; Bing R.; Kiang J.; Bashir S.; Spath N.; Stelzle D.; Mortimer K.; Bularga A.; Doudesis D.; Joshi S. S.; Strachan F.; Gumy S.; Adair-Rohani H.; Attia E. F.; Chung M. H.; Miller M. R.; Newby D. E.; Mills N. L.; McAllister D. A.; Shah A. S. V. Adverse health effects associated with household air pollution: a systematic review, meta-analysis, and burden estimation study. Lancet Global Health 2020, 8 (11), e1427–e1434. 10.1016/S2214-109X(20)30343-0. 33069303 PMC7564377

[ref10] Hassan Bhat T.; Jiawen G.; Farzaneh H. Air pollution health risk assessment (ap-hra), principles and applications. Int. J. Environ. Res. Publ. Health 2021, 18 (4), 1935 10.3390/ijerph18041935. PMC7922529 33671274

[ref11] Hauschild M. Z.; Huijbregts M. A. Introducing life cycle impact assessment. Life Cycle Impact Assessment; Springer, 2015; pp 1–16.

[ref12] Morawska L.; Afshari A.; Bae G. N.; Buonanno G.; Chao C. Y. H.; Hänninen O.; Hofmann W.; Isaxon C.; Jayaratne E. R.; Pasanen P.; Salthammer T.; Waring M.; Wierzbicka A. Indoor aerosols: from personal exposure to risk assessment. Indoor air 2013, 23 (6), 462–487. 10.1111/ina.12044. 23574389

[ref13] Liu W.; Sun Y.; Liu N.; Hou J.; Huo X.; Zhao Y.; Zhang Y.; Deng F.; Kan H.; Zhao Z.; Huang C.; Zhao B.; Zeng X.; Qian H.; Zheng X.; Liu W.; Mo J.; Sun C.; Su C.; Zou Z.; Li H.; Guo J.; Bu Z. Indoor exposure to phthalates and its burden of disease in china. Indoor air 2022, 32 (4), e13030 10.1111/ina.13030. 35481931

[ref14] Murray C. J. L.; Aravkin A. Y.; Zheng P.; Abbafati C.; Abbas K. M.; Abbasi-Kangevari M.; Abd-Allah F.; Abdelalim A.; Abdollahi M.; Abdollahpour I.; Abegaz K. H.; Abolhassani H.; Aboyans V.; Abreu L. G.; Abrigo M. R. M.; Abualhasan A.; Abu-Raddad L. J.; Abushouk A. I.; Adabi M.; Adekanmbi V.; Adeoye A. M.; Adetokunboh O. O.; Adham D.; Advani S. M.; Agarwal G.; Aghamir S. M. K.; Agrawal A.; Ahmad T.; Ahmadi K.; Ahmadi M.; Ahmadieh H.; Ahmed M. B.; Akalu T. Y.; Akinyemi R. O.; Akinyemiju T.; Akombi B.; Akunna C. J.; Alahdab F.; Al-Aly Z.; Alam K.; Alam S.; Alam T.; Alanezi F. M.; Alanzi T. M.; Alemu B. w.; Alhabib K. F.; Ali M.; Ali S.; Alicandro G.; Alinia C.; Alipour V.; Alizade H.; Aljunid S. M.; Alla F.; Allebeck P.; Almasi-Hashiani A.; Al-Mekhlafi H. M.; Alonso J.; Altirkawi K. A.; Amini-Rarani M.; Amiri F.; Amugsi D. A.; Ancuceanu R.; Anderlini D.; Anderson J. A.; Andrei C. L.; Andrei T.; Angus C.; Anjomshoa M.; Ansari F.; Ansari-Moghaddam A.; Antonazzo I. C.; Antonio C. A. T.; Antony C. M.; Antriyandarti E.; Anvari D.; Anwer R.; Appiah S. C. Y.; Arabloo J.; Arab-Zozani M.; Ariani F.; Armoon B.; Ärnlöv J.; Arzani A.; Asadi-Aliabadi M.; Asadi-Pooya A. A.; Ashbaugh C.; Assmus M.; Atafar Z.; Atnafu D. D.; Atout M. M. W.; Ausloos F.; Ausloos M.; Ayala Quintanilla B. P.; Ayano G.; Ayanore M. A.; Azari S.; Azarian G.; Azene Z. N.; Badawi A.; Badiye A. D.; Bahrami M. A.; Bakhshaei M. H.; Bakhtiari A.; Bakkannavar S. M.; Baldasseroni A.; Ball K.; Ballew S. H.; Balzi D.; Banach M.; Banerjee S. K.; Bante A. B.; Baraki A. G.; Barker-Collo S. L.; Bärnighausen T. W.; Barrero L. H.; Barthelemy C. M.; Barua L.; Basu S.; Baune B. T.; Bayati M.; Becker J. S.; Bedi N.; Beghi E.; Béjot Y.; Bell M. L.; Bennitt F. B.; Bensenor I. M.; Berhe K.; Berman A. E.; Bhagavathula A. S.; Bhageerathy R.; Bhala N.; Bhandari D.; Bhattacharyya K.; Bhutta Z. A.; Bijani A.; Bikbov B.; Bin Sayeed M. S.; Biondi A.; Birihane B. M.; Bisignano C.; Biswas R. K.; Bitew H.; Bohlouli S.; Bohluli M.; Boon-Dooley A. S.; Borges G.; Borzì A. M.; Borzouei S.; Bosetti C.; Boufous S.; Braithwaite D.; Breitborde N. J. K.; Breitner S.; Brenner H.; Briant P. S.; Briko A. N.; Briko N. I.; Britton G. B.; Bryazka D.; Bumgarner B. R.; Burkart K.; Burnett R. T.; Burugina Nagaraja S.; Butt Z. A.; Caetano dos Santos F. L.; Cahill L. E.; Cámera L. L. A.; Campos-Nonato I. R.; Cárdenas R.; Carreras G.; Carrero J. J.; Carvalho F.; Castaldelli-Maia J. M.; Castañeda-Orjuela C. A.; Castelpietra G.; Castro F.; Causey K.; Cederroth C. R.; Cercy K. M.; Cerin E.; Chandan J. S.; Chang K. L.; Charlson F. J.; Chattu V. K.; Chaturvedi S.; Cherbuin N.; Chimed-Ochir O.; Cho D. Y.; Choi J. Y. J.; Christensen H.; Chu D. T.; Chung M. T.; Chung S. C.; Cicuttini F. M.; Ciobanu L. G.; Cirillo M.; Classen T. K. D.; Cohen A. J.; Compton K.; Cooper O. R.; Costa V. M.; Cousin E.; Cowden R. G.; Cross D. H.; Cruz J. A.; Dahlawi S. M. A.; Damasceno A. A. M.; Damiani G.; Dandona L.; Dandona R.; Dangel W. J.; Danielsson A. K.; Dargan P. I.; Darwesh A. M.; Daryani A.; Das J. K.; Das Gupta R.; das Neves J.; Dávila-Cervantes C. A.; Davitoiu D. V.; De Leo D.; Degenhardt L.; DeLang M.; Dellavalle R. P.; Demeke F. M.; Demoz G. T.; Demsie D. G.; Denova-Gutiérrez E.; Dervenis N.; Dhungana G. P.; Dianatinasab M.; Dias da Silva D.; Diaz D.; Dibaji Forooshani Z. S.; Djalalinia S.; Do H. T.; Dokova K.; Dorostkar F.; Doshmangir L.; Driscoll T. R.; Duncan B. B.; Duraes A. R.; Eagan A. W.; Edvardsson D.; El Nahas N.; El Sayed I.; El Tantawi M.; Elbarazi I.; Elgendy I. Y.; El-Jaafary S. I.; Elyazar I. R.; Emmons-Bell S.; Erskine H. E.; Eskandarieh S.; Esmaeilnejad S.; Esteghamati A.; Estep K.; Etemadi A.; Etisso A. E.; Fanzo J.; Farahmand M.; Fareed M.; Faridnia R.; Farioli A.; Faro A.; Faruque M.; Farzadfar F.; Fattahi N.; Fazlzadeh M.; Feigin V. L.; Feldman R.; Fereshtehnejad S. M.; Fernandes E.; Ferrara G.; Ferrari A. J.; Ferreira M. L.; Filip I.; Fischer F.; Fisher J. L.; Flor L. S.; Foigt N. A.; Folayan M. O.; Fomenkov A. A.; Force L. M.; Foroutan M.; Franklin R. C.; Freitas M.; Fu W.; Fukumoto T.; Furtado J. M.; Gad M. M.; Gakidou E.; Gallus S.; Garcia-Basteiro A. L.; Gardner W. M.; Geberemariyam B. S.; Gebreslassie A. A. A. A.; Geremew A.; Gershberg Hayoon A.; Gething P. W.; Ghadimi M.; Ghadiri K.; Ghaffarifar F.; Ghafourifard M.; Ghamari F.; Ghashghaee A.; Ghiasvand H.; Ghith N.; Gholamian A.; Ghosh R.; Gill P. S.; Ginindza T. G. G.; Giussani G.; Gnedovskaya E. V.; Goharinezhad S.; Gopalani S. V.; Gorini G.; Goudarzi H.; Goulart A. C.; Greaves F.; Grivna M.; Grosso G.; Gubari M. I. M.; Gugnani H. C.; Guimarães R. A.; Guled R. A.; Guo G.; Guo Y.; Gupta R.; Gupta T.; Haddock B.; Hafezi-Nejad N.; Hafiz A.; Haj-Mirzaian A.; Haj-Mirzaian A.; Hall B. J.; Halvaei I.; Hamadeh R. R.; Hamidi S.; Hammer M. S.; Hankey G. J.; Haririan H.; Haro J. M.; Hasaballah A. I.; Hasan M. M.; Hasanpoor E.; Hashi A.; Hassanipour S.; Hassankhani H.; Havmoeller R. J.; Hay S. I.; Hayat K.; Heidari G.; Heidari-Soureshjani R.; Henrikson H. J.; Herbert M. E.; Herteliu C.; Heydarpour F.; Hird T. R.; Hoek H. W.; Holla R.; Hoogar P.; Hosgood H. D.; Hossain N.; Hosseini M.; Hosseinzadeh M.; Hostiuc M.; Hostiuc S.; Househ M.; Hsairi M.; Hsieh V. C. r.; Hu G.; Hu K.; et al. Global burden of 87 risk factors in 204 countries and territories, 1990–2019: a systematic analysis for the Global Burden of Disease Study 2019. Lancet 2020, 396 (10258), 1223–1249. 10.1016/s0140-6736(20)30752-2. 33069327 PMC7566194

[ref15] Huijbregts M. A.; Rombouts L. J.; Ragas A. M.; van de Meent D. Human-toxicological effect and damage factors of carcinogenic and noncarcinogenic chemicals for life cycle impact assessment. Integrated Environmental Assessment and Management: An International Journal 2005, 1 (3), 181–244. 10.1897/2004-007R.1. 16639884

[ref16] Rosenbaum R. K.; Meijer A.; Demou E.; Hellweg S.; Jolliet O.; Lam N. L.; Margni M.; McKone T. E. Indoor air pollutant exposure for life cycle assessment: regional health impact factors for households. Environ. Sci. Technol. 2015, 49 (21), 12823–12831. 10.1021/acs.est.5b00890. 26444519

[ref17] Wu S. R.; Apul D. Framework for integrating indoor air quality impacts into life cycle assessments of buildings and building related products. Journal of Green Building 2015, 10 (1), 127–149. 10.3992/jgb.10.1.127.

[ref18] Logue J. M.; Price P. N.; Sherman M. H.; Singer B. C. A method to estimate the chronic health impact of air pollutants in us residences. Environ. Health Perspect. 2012, 120 (2), 216–222. 10.1289/ehp.1104035. 22094717 PMC3279453

[ref19] Fazli T.; Stephens B. Development of a nationally representative set of combined building energy and indoor air quality models for us residences. Build. Environ. 2018, 136, 198–212. 10.1016/j.buildenv.2018.03.047.

[ref20] Turner W. J.; Logue J. M.; Wray C. P. A combined energy and iaq assessment of the potential value of commissioning residential mechanical ventilation systems. Build. Environ. 2013, 60, 194–201. 10.1016/j.buildenv.2012.10.016.

[ref21] Diaz Lozano Patino E.; Siegel J. A. Indoor environmental quality in social housing: A literature review. Build. Environ. 2018, 131, 231–241. 10.1016/j.buildenv.2018.01.013.

[ref22] Ben-David T.; Waring M. S. Impact of natural versus mechanical ventilation on simulated indoor air quality and energy consumption in offices in fourteen us cities. Build. Environ. 2016, 104, 320–336. 10.1016/j.buildenv.2016.05.007.

[ref23] Zaatari M.; Novoselac A.; Siegel J. Impact of ventilation and filtration strategies on energy consumption and exposures in retail stores. Build. Environ. 2016, 100, 186–196. 10.1016/j.buildenv.2016.01.026.

[ref24] Aldred J. R.; Darling E.; Morrison G.; Siegel J.; Corsi R. Benefit-cost analysis of commercially available activated carbon filters for indoor ozone removal in single-family homes. Indoor Air 2016, 26 (3), 501–512. 10.1111/ina.12220. 25952610

[ref25] Morantes G.; Jones B.; Sherman M. H.; Molina C. A preliminary assessment of the health impacts of indoor air contaminants determined using the daly metric. Int. J. Vent. 2023, 22 (4), 307–316. 10.1080/14733315.2023.2198800.

[ref26] Stanley W. B. M.; Bayer C. W. Determining contaminants of concern when implementing ashrae standard 62.1 indoor air quality procedure. Proceedings of Healthy Buildings, 2009; p 4.

[ref27] Parthasarathy S. Ventilation relevant contaminants of concern in commercial buildings screening process and results. Tech. Rep.; USDOE Office of Science (SC), 2011;.

[ref28] Sherman M. H.; Walker I. S.; Logue J. M. Equivalence in ventilation and indoor air quality. HVAC R Res. 2012, 18 (4), 760–773. 10.1080/10789669.2012.667038.

[ref29] Walker I.; Guyot G.; Sherman M.; Jordan C. Residential smart ventilation: A review. Tech. Rep.; Lawrence Berkeley National Lab.(LBNL): Berkeley, CA (United States), 2022;.

[ref30] Guyot G.; Walker I. S.; Sherman M. H. Performance based approaches in standards and regulations for smart ventilation in residential buildings: a summary review. Int. J. Vent. 2019, 18 (2), 96–112. 10.1080/14733315.2018.1435025.

[ref31] Sherman M.; Fairey P.; Crawford R. Impacts of unvented space heaters. ASHRAE J. 2022, 64 (5), 32–49.

[ref32] Puri M.; Gandhi K.; Kumar M. S. Emerging environmental contaminants: A global perspective on policies and regulations. J. Environ. Manage. 2023, 332, 117344 10.1016/j.jenvman.2023.117344. 36736081

[ref33] Hess-Kosa K. Indoor Air Quality: The Latest Sampling and Analytical Methods; CRC Press, 2018.

[ref34] U. E. P. Agency. Technical Support Document: Epa’s 2011 National-Scale Air Toxics Assessment, 2015;.

[ref35] Oberschelp C.; Pfister S.; Hellweg S. Globally regionalized monthly life cycle impact assessment of particulate matter. Environ. Sci. Technol. 2020, 54 (24), 16028–16038. 10.1021/acs.est.0c05691. 33226786

[ref36] ASHRAE. Standard 62.2–2019: Ventilation and Acceptable Indoor Air Quality in Residential Buildings; American Society of Heating, Refrigerating, and Air Conditioning Engineers, Inc: Atlanta, GA, 2019;.

[ref37] Spengler J. D.; McCarthy J. F.; Samet J. M. Indoor Air Quality Handbook; McGraw Hill Professional, 2000.

[ref38] Liu C.; Chen R.; Sera F.; Vicedo-Cabrera A. M.; Guo Y.; Tong S.; Lavigne E.; Correa P. M.; Ortega N. V.; Achilleos S.; Roye D.; Jaakkola J. J.; Ryti N.; Pascal M.; Schneider A.; Breitner S.; Entezari A.; Mayvaneh F.; Raz R.; Honda Y.; Hashizume M.; Ng C. F. S.; Gaio V.; Madureira J.; Holobaca I.-H.; Tobias A.; Íñiguez C.; Guo Y. L.; Pan S.-C.; Masselot P.; Bell M. L.; Zanobetti A.; Schwartz J.; Gasparrini A.; Kan H. Interactive effects of ambient fine particulate matter and ozone on daily mortality in 372 cities: two stage time series analysis. bmj 2023, 383, e075203 10.1136/bmj-2023-075203. 37793695 PMC10548261

[ref39] Siddika N.; Rantala A. K.; Antikainen H.; Balogun H.; Amegah A. K.; Ryti N. R.; Kukkonen J.; Sofiev M.; Jaakkola M. S.; Jaakkola J. J. Synergistic effects of prenatal exposure to fine particulate matter (pm2. 5) and ozone (o3) on the risk of preterm birth: A population-based cohort study. Environ. Res. 2019, 176, 108549 10.1016/j.envres.2019.108549. 31252204

[ref40] Huang Y.-C. T.; Rappold A. G.; Graff D. W.; Ghio A. J.; Devlin R. B. Synergistic effects of exposure to concentrated ambient fine pollution particles and nitrogen dioxide in humans. Inhal. Toxicol. 2012, 24 (12), 790–797. 10.3109/08958378.2012.718809. 23033993

[ref41] Ku T.; Chen M.; Li B.; Yun Y.; Li G.; Sang N. Synergistic effects of particulate matter (pm2. 5) and sulfur dioxide (so2) on neurodegeneration via the microrna-mediated regulation of tau phosphorylation. Toxicol. Res. 2017, 6 (1), 7–16. 10.1039/C6TX00314A. PMC6060696 30090473

[ref42] Billionnet C.; Sherrill D.; Annesi-Maesano I. Estimating the health effects of exposure to multi-pollutant mixture. Ann. Epidemiol. 2012, 22 (2), 126–141. 10.1016/j.annepidem.2011.11.004. 22226033

[ref43] Yu L.; Liu W.; Wang X.; Ye Z.; Tan Q.; Qiu W.; Nie X.; Li M.; Wang B.; Chen W. A review of practical statistical methods used in epidemiological studies to estimate the health effects of multi-pollutant mixture. Environ. Pollut. 2022, 306, 119356 10.1016/j.envpol.2022.119356. 35487468

[ref44] Socianu S.; Bopp S. K.; Govarts E.; Gilles L.; Buekers J.; Kolossa-Gehring M.; Backhaus T.; Franco A. Chemical mixtures in the eu population: composition and potential risks. Int. J. Environ. Res. Publ. Health 2022, 19 (10), 6121 10.3390/ijerph19106121. PMC9141134 35627658

[ref45] More S. J.; Bampidis V.; Benford D.; Bennekou S. H.; Bragard C.; Halldorsson T. I.; Hernández-Jerez A. F.; Koutsoumanis K.; Naegeli H.; Schlatter J. R.; Silano V.; Nielsen S. S.; Schrenk D.; Turck D.; Younes M.; Benfenati E.; Castle L.; Cedergreen N.; Hardy A.; Laskowski R.; Leblanc J. C.; Kortenkamp A.; Ragas A.; Posthuma L.; Svendsen C.; Solecki R.; Testai E.; Dujardin B.; Kass G. E.; Manini P.; Jeddi M. Z.; Dorne J.-L. C.; Hogstrand C.; Guidance on harmonised methodologies for human health, animal health and ecological risk assessment of combined exposure to multiple chemicals. EFSA J. 2019, 17 (3), e05634 10.2903/j.efsa.2019.5634. 32626259 PMC7009070

[ref46] Kortenkamp A.; Backhaus T.; Faust M. State of the art review of mixture toxicity. Report to the Commission of the European Union; Directorate General for the Environment, 2009; p 22.

[ref47] Rudén C.; Backhaus T.; Bergman D.; Faust M.; Molander L.; Slunge D. Future Chemical Risk Management: Accounting for Combination Effects and Assessing Chemicals in Groups, Swedish Government Inquiries-Swedish Government Official Reports. Sou 45, 2019;.

[ref48] Martin O. V. Synergistic effects of chemical mixtures: how frequent is rare?. Curr. Opin. Toxicol. 2023, 36, 100424 10.1016/j.cotox.2023.100424.

[ref49] Backhaus T. The mixture assessment or allocation factor: conceptual background, estimation algorithms and a case study example. Environ. Sci. Eur. 2023, 35 (1), 55 10.1186/s12302-023-00757-w.

[ref50] Martin O.; Scholze M.; Ermler S.; McPhie J.; Bopp S. K.; Kienzler A.; Parissis N.; Kortenkamp A. Ten years of research on synergisms and antagonisms in chemical mixtures: A systematic review and quantitative reappraisal of mixture studies. Environ. Int. 2021, 146, 106206 10.1016/j.envint.2020.106206. 33120228

[ref51] Mauderly J. L.; Samet J. M. Is there evidence for synergy among air pollutants in causing health effects?. Environ. Health Perspect. 2009, 117 (1), 1–6. 10.1289/ehp.11654. 19165380 PMC2627851

[ref52] Li Z.-H.; Wang X.-M.; Xiang J.-X.; Nan Y.; Chen Y.-J.; Zhang P.-D.; Liu D.; Shen D.; Zhang X.-R.; Zhong W.-F.; Chen P.-L.; Huang Q.-M.; Song W.-Q.; Qiu C.-S.; Liang F.; Li C.; Mao C. Associations of long-term joint exposure to various ambient air pollutants with all-cause and cause-specific mortality: evidence from a large population-based cohort study. Environ. Sci. Pollut. Res. 2023, 30, 84357–84367. 10.1007/s11356-023-28224-2. 37365359

[ref53] van Zelm R.; Preiss P.; van Goethem T.; Van Dingenen R.; Huijbregts M. Regionalized life cycle impact assessment of air pollution on the global scale: Damage to human health and vegetation. Atmos. Environ. 2016, 134, 129–137. 10.1016/j.atmosenv.2016.03.044.

[ref54] Van Zelm R.; Huijbregts M. A.; den Hollander H. A.; Van Jaarsveld H. A.; Sauter F. J.; Struijs J.; Van Wijnen H. J.; Van de Meent D. European characterization factors for human health damage of pm10 and ozone in life cycle impact assessment. Atmos. Environ. 2008, 42 (3), 441–453. 10.1016/j.atmosenv.2007.09.072.

[ref55] Rojas-Rueda D.; Vrijheid M.; Robinson O.; Gunn Marit A.; Gražulevičienė R.; Slama R.; Nieuwenhuijsen M. Environmental burden of childhood disease in europe. Int. J. Environ. Res. Publ. Health 2019, 16 (6), 1084 10.3390/ijerph16061084. PMC6466397 30917598

[ref56] Guan Y.; Xiao Y.; Wang Y.; Zhang N.; Chu C. Assessing the health impacts attributable to pm2.5 and ozone pollution in 338 chinese cities from 2015 to 2020. Environ. Pollut. 2021, 287, 117623 10.1016/j.envpol.2021.117623. 34171728

[ref57] Gronlund C. J.; Humbert S.; Shaked S.; O’Neill M. S.; Jolliet O. Characterizing the burden of disease of particulate matter for life cycle impact assessment. Atmosphere & Health 2015, 8 (1), 29–46. 10.1007/s11869-014-0283-6. PMC4426268 25972992

[ref58] Fantke P.; McKone T. E.; Tainio M.; Jolliet O.; Apte J. S.; Stylianou K. S.; Illner N.; Marshall J. D.; Choma E. F.; Evans J. S. Global effect factors for exposure to fine particulate matter. Environ. Sci. Technol. 2019, 53 (12), 6855–6868. 10.1021/acs.est.9b01800. 31132267 PMC6613786

[ref59] Huijbregts M.; Steinmann Z.; Elshout P.; Stam G.; Verones F.; Vieira M.; Zijp M.; Hollander A.; van Zelm R. Recipe2016: a harmonised life cycle impact assessment method at midpoint and endpoint level. Int. J. Life Cycle Assess. 2017, 22 (2), 138–147. 10.1007/s11367-016-1246-y.

[ref60] Goel A.; Ola D.; Veetil A. V. Burden of disease for workers attributable to exposure through inhalation of ppahs in rspm from cooking fumes. Environ. Sci. Pollut. Res. 2019, 26 (9), 8885–8894. 10.1007/s11356-019-04242-x. 30719671

[ref61] Kvasnicka J.; Stylianou K. S.; Nguyen V. K.; Huang L.; Chiu W. A.; Burton G. A. Jr; Semrau J.; Jolliet O. Human health benefits from fish consumption vs. risks from inhalation exposures associated with contaminated sediment remediation: dredging of the hudson river. Environ. Health Perspect. 2019, 127 (12), 127004 10.1289/ehp5034. 31834828 PMC6957280

[ref62] Petrov O.; Bi X.; Lau A. Impact assessment of biomass-based district heating systems in densely populated communities. part ii: Would the replacement of fossil fuels improve ambient air quality and human health?. Atmos. Environ. 2017, 161, 191–199. 10.1016/j.atmosenv.2017.05.001.

[ref63] Tang L.; Nagashima T.; Hasegawa K.; Ohara T.; Sudo K.; Itsubo N. Development of human health damage factors for pm2. 5 based on a global chemical transport model. Int. J. Life Cycle Assess. 2018, 23 (12), 2300–2310. 10.1007/s11367-014-0837-8.

[ref64] Bulle C.; Margni M.; Patouillard L.; Boulay A.-M.; Bourgault G.; De Bruille V.; Cao V.; Hauschild M.; Henderson A.; Humbert S.; Kashef-Haghighi S.; Kounina A.; Laurent A.; Levasseur A.; Liard G.; Rosenbaum R. K.; Roy P.-O.; Shaked S.; Fantke P.; Jolliet O. Impact world+: a globally regionalized life cycle impact assessment method. Int. J. Life Cycle Assess. 2019, 24 (9), 1653–1674. 10.1007/s11367-019-01583-0.

[ref65] Maury-Micolier A.; Huang L.; Taillandier F.; Sonnemann G.; Jolliet O. A life cycle approach to indoor air quality in designing sustainable buildings: Human health impacts of three inner and outer insulations. Build. Environ. 2023, 230, 109994 10.1016/j.buildenv.2023.109994.

[ref66] Meijer A.; Huijbregts M.; Reijnders L. Human health damages due to indoor sources of organic compounds and radioactivity in life cycle impact assessment of dwellings-part 1: Characterisation factors (8 pp). Int. J. Life Cycle Assess. 2005, 10 (5), 309–316. 10.1065/lca2004.12.194.1.

[ref67] Meijer A.; Huijbregts M.; Reijnders L. Human health damages due to indoor sources of organic compounds and radioactivity in life cycle impact assessment of dwellings-part 2: Damage scores (10 pp). Int. J. Life Cycle Assess. 2005, 10 (6), 383–392. 10.1065/lca2004.12.194.2.

[ref68] Hellweg S.; Demou E.; Bruzzi R.; Meijer A.; Rosenbaum R. K.; Huijbregts M. A.; McKone T. E. Integrating human indoor air pollutant exposure within life cycle impact assessment. Environ. Sci. Technol. 2009, 43 (6), 1670–1679. 10.1021/es8018176. 19368156 PMC2659811

[ref69] Fantke P.; Jolliet O.; Apte J. S.; Hodas N.; Evans J.; Weschler C. J.; Stylianou K. S.; Jantunen M.; McKone T. E. Characterizing aggregated exposure to primary particulate matter: recommended intake fractions for indoor and outdoor sources. Environ. Sci. Technol. 2017, 51 (16), 9089–9100. 10.1021/acs.est.7b02589. 28682605

[ref70] Phillips L.; Moya J. The evolution of epa’s exposure factors handbook and its future as an exposure assessment resource. J. Expo. Sci. Environ. Epidemiol. 2013, 23 (1), 13–21. 10.1038/jes.2012.77. 22805985

[ref71] Inc I. E. Health and welfare benefits analyses to support the second section 812 benefit-cost analysis of the clean air act. Tech. Rep.; Industrial Economics, Inc.: Cambridge, MA, 2011;.

[ref72] Eaton D. L.; Gilbert S. G. Principles of toxicology, Casarett & Doull’s Toxicology. The Basic Science of Poisons; Klaassen C. D., Ed., 2008; pp 11–34.

[ref73] Gupta P. Problem Solving Questions in Toxicology; Springer, 2020;.

[ref74] Fantke P.; Bijster M.; Guignard C.; Hauschild M.; Huijbregts M.; Jolliet O.; Kounina A.; Magaud V.; Margni M.; Mckone T.; Posthuma L.; Rosenbaum R.; van de Meent D.; van Zelm R.; Fantke P. USEtox 2.0: Documentation (Version 1); USEtox International Center, 2017, [Departement_IRSTEA]Ecotechnologies [TR1_IRSTEA]INSPIRE. URL https://hal.inrae.fr/hal-02607348.

[ref75] Rosenbaum R. K.; Bachmann T. M.; Gold L. S.; Huijbregts M. A.; Jolliet O.; Juraske R.; Koehler A.; Larsen H. F.; MacLeod M.; Margni M.; McKone T. E.; Payet J.; Schuhmacher M.; van de Meent D.; Hauschild M. Z. Usetox—the unep-setac toxicity model: recommended characterisation factors for human toxicity and freshwater ecotoxicity in life cycle impact assessment. Int. J. Life Cycle Assess. 2008, 13 (7), 532–546. 10.1007/s11367-008-0038-4.

[ref76] Jolliet O.; Rosenbaum R.; McKone T. E.; Scheringer M.; Straalen N. v.; Wania F. Establishing a Framework for Life Cycle Toxicity Assessment. Findings of the Lausanne Review Workshop (4 pp). Int. J. Life Cycle Assess. 2006, 11 (3), 209–212. 10.1065/lca2006.03.002.

[ref77] Pennington D.; Margni M.; Payet J.; Jolliet O. Risk and regulatory hazard-based toxicological effect indicators in life-cycle assessment (lca). Hum. Ecol. Risk Assess. 2006, 12 (3), 450–475. 10.1080/10807030600561667.

[ref78] McKone T. E.; Kyle A.; Jolliet O.; Irving Olsen S.; Hauschild M. Dose-response modeling for life cycle impact assessment: Findings of the portland review workshop. Int. J. Life Cycle Assess. 2006, 11 (2), 137–140. 10.1065/lca2006.02.005.

[ref79] Crettaz P.; Pennington D.; Rhomberg L.; Brand K.; Jolliet O. Assessing Human Health Response in Life Cycle Assessment Using ED_10_s and DALYs: Part 1—Cancer Effects. Int. J. 2002, 22 (5), 931–946. 10.1111/1539-6924.00262. 12442990

[ref80] Bhopal R. S. Concepts of Epidemiology: Integrating the Ideas, Theories, Principles, and Methods of Epidemiology; Oxford University Press, 2016;.

[ref81] Health Risk Assessment of Air Pollution: General Principles, World Health Organization (WHO). Regional Office for Europe, 2016.

[ref82] Martenies S. E.; Wilkins D.; Batterman S. A. Health impact metrics for air pollution management strategies. Environ. Int. 2015, 85, 84–95. 10.1016/j.envint.2015.08.013. 26372694 PMC4648637

[ref83] Logue J.; McKone T.; Sherman M.; Singer B. Hazard assessment of chemical air contaminants measured in residences. Indoor air 2011, 21 (2), 92–109. 10.1111/j.1600-0668.2010.00683.x. 21392118

[ref84] Vardoulakis S.; Giagloglou E.; Steinle S.; Davis A.; Sleeuwenhoek A.; Galea K. S.; Dixon K.; Crawford J. O. Indoor exposure to selected air pollutants in the home environment: A systematic review. Int. J. Environ. Res. Publ. Health 2020, 17 (23), 8972 10.3390/ijerph17238972. PMC7729884 33276576

[ref85] Gonzalez-Martin J.; Kraakman N. J. R.; Perez C.; Lebrero R.; Munoz R. A state–of–the-art review on indoor air pollution and strategies for indoor air pollution control. Chemosphere 2021, 262, 128376 10.1016/j.chemosphere.2020.128376. 33182138

[ref86] Richmond-Bryant J. In Defense of the Weight-of-Evidence Approach to Literature Review in the Integrated Science Assessment. Epidemiology (Cambridge, Mass.) 2020, 31 (6), 755–757. 10.1097/EDE.0000000000001254. 32897910 PMC7541567

[ref87] USEPA. Integrated Science Assessment for Particulate Matter; US Environmental Protection Agency: Washington DC, USA, 2019; p 1967.

[ref88] Schraufnagel D. E. The health effects of ultrafine particles. Exp. Mol. Med. 2020, 52 (3), 311–317. 10.1038/s12276-020-0403-3. 32203102 PMC7156741

[ref89] Ohlwein S.; Kappeler R.; Kutlar Joss M.; Künzli N.; Hoffmann B. Health effects of ultrafine particles: a systematic literature review update of epidemiological evidence. Int. J. Publ. Health 2019, 64, 547–559. 10.1007/s00038-019-01202-7. 30790006

[ref90] Shaked S.; Crettaz P.; Saade-Sbeih M.; Jolliet O.; Jolliet A. Environmental Life Cycle Assessment; CRC Press, 2015.

[ref91] Imbeault Tétreault H.; Jolliet O.; Deschênes L.; Rosenbaum R. K. Analytical propagation of uncertainty in life cycle assessment using matrix formulation. J. Ind. Ecol. 2013, 17 (4), 485–492. 10.1111/jiec.12001.

[ref92] Ciroth A.; Muller S.; Weidema B.; Lesage P. Empirically based uncertainty factors for the pedigree matrix in ecoinvent. Int. J. Life Cycle Assess. 2016, 21, 1338–1348. 10.1007/s11367-013-0670-5.

[ref93] Slob W. Uncertainty analysis in multiplicative models. Risk Anal. 1994, 14 (4), 571–576. 10.1111/j.1539-6924.1994.tb00271.x.

[ref94] Ott W. R. A physical explanation of the lognormality of pollutant concentrations. J. Air Waste Manage. Assoc. 1990, 40 (10), 1378–1383. 10.1080/10473289.1990.10466789. 2257125

[ref95] Crow E. L.; Shimizu K. Lognormal Distributions; Marcel Dekker: New York, 1987.

[ref96] Blackwood L. G. The lognormal distribution, environmental data, and radiological monitoring. Environ. Monit. Assess. 1992, 21 (3), 193–210. 10.1007/BF00399687. 24234486

[ref97] Jia C.; D’Souza J.; Batterman S. Distributions of personal voc exposures: a population-based analysis. Environ. Int. 2008, 34 (7), 922–931. 10.1016/j.envint.2008.02.002. 18378311

[ref98] Schmid C. H.; Stijnen T.; White I. Handbook of Meta-Analysis; CRC Press, 2020.

[ref99] DerSimonian R.; Laird N. Meta-analysis in clinical trials. Contr. Clin. Trials 1986, 7 (3), 177–188. 10.1016/0197-2456(86)90046-2. 3802833

[ref100] L. StataCorp. Stata Meta-Analysis Reference Manual: Release 16; Stata Press: TX, 2019;.

[ref101] Daly C.; Soobiah C. Software to conduct a meta-analysis and network meta-analysis. Meta-Research 2022, 2345, 223–244. 10.1007/978-1-0716-1566-9_14. 34550594

[ref102] Fisher D. J. Two-stage individual participant data meta-analysis and generalized forest plots. STATA J. 2015, 15 (2), 369–396. 10.1177/1536867X1501500203.

[ref103] Harris R. J.; Deeks J. J.; Altman D. G.; Bradburn M. J.; Harbord R. M.; Sterne J. A. Metan: fixed-and random-effects meta-analysis. STATA J. 2008, 8 (1), 3–28. 10.1177/1536867X0800800102.

[ref104] Vanker A.; Barnett W.; Nduru P. M.; Gie R. P.; Sly P. D.; Zar H. J. Home environment and indoor air pollution exposure in an african birth cohort study. Sci. Total Environ. 2015, 536, 362–367. 10.1016/j.scitotenv.2015.06.136. 26231768

[ref105] Sarigiannis D. A.; Karakitsios S. P.; Gotti A.; Liakos I. L.; Katsoyiannis A. Exposure to major volatile organic compounds and carbonyls in european indoor environments and associated health risk. Environ. Int. 2011, 37 (4), 743–765. 10.1016/j.envint.2011.01.005. 21354626

[ref106] Arku R. E.; Birch A.; Shupler M.; Yusuf S.; Hystad P.; Brauer M. Characterizing exposure to household air pollution within the prospective urban rural epidemiology (pure) study. Environ. Int. 2018, 114, 307–317. 10.1016/j.envint.2018.02.033. 29567495 PMC5899952

[ref107] Morawska L.; Ayoko G.; Bae G.; Buonanno G.; Chao C.; Clifford S.; Fu S.; Hänninen O.; He C.; Isaxon C.; Mazaheri M.; Salthammer T.; Waring M.; Wierzbicka A. Airborne particles in indoor environment of homes, schools, offices and aged care facilities: The main routes of exposure. Environ. Int. 2017, 108, 75–83. 10.1016/j.envint.2017.07.025. 28802170

[ref108] Ye W.; Zhang X.; Gao J.; Cao G.; Zhou X.; Su X. Indoor air pollutants, ventilation rate determinants and potential control strategies in chinese dwellings: a literature review. Sci. Total Environ. 2017, 586, 696–729. 10.1016/j.scitotenv.2017.02.047. 28215812

[ref109] Nishihama Y.; Jung C.-R.; Nakayama S. F.; Tamura K.; Isobe T.; Michikawa T.; Iwai-Shimada M.; Kobayashi Y.; Sekiyama M.; Taniguchi Y.; Yamazaki S. Indoor air quality of 5,000 households and its determinants. part a: Particulate matter (pm2. 5 and pm10–2.5) concentrations in the japan environment and children’s study. Environ. Res. 2021, 198, 111196 10.1016/j.envres.2021.111196. 33939980

[ref110] Halios C. H.; Landeg-Cox C.; Lowther S. D.; Middleton A.; Marczylo T.; Dimitroulopoulou S. Chemicals in european residences–part i: A review of emissions, concentrations and health effects of volatile organic compounds (vocs). Sci. Total Env. 2022, 839, 156201 10.1016/j.scitotenv.2022.156201. 35623519

[ref111] Ilacqua V.; Scharko N.; Zambrana J.; Malashock D. Survey of residential indoor particulate matter measurements 1990–2019. Indoor air 2022, 32 (7), e13057 10.1111/ina.13057. 35904386 PMC10499005

[ref112] Sacks J.; Buckley B.; Deflorio-Barker S.; Jenkins S.; Kirrane E.; Krajewski A.; Luben T.; McDow S.; Stewart M.; Dubois J.-J.; Park K.; Rice R. B. Supplement to the 2019 integrated science assessment for particulate matter. Tech. Rep.; US Environmental Protection Agency, 2022;. 36630543

[ref113] Braubach M.; Jacobs D. E.; Ormandy D. Environmental Burden of Disease Associated with Inadequate Housing: A Method Guide to the Quantification of Health Effects of Selected Housing Risks in the WHO European Region; World Health Organization. Regional Office for Europe, 2011;.

[ref114] Shan X.; Tian X.; Wang B.; He L.; Zhang L.; Xue B.; Liu C.; Zheng L.; Yu Y.; Luo B. A global burden assessment of lung cancer attributed to residential radon exposure during 1990–2019. Indoor air 2022, 32 (10), e13120 10.1111/ina.13120. 36305076

[ref115] Martin O.; Martin S.; Kortenkamp A. Dispelling urban myths about default uncertainty factors in chemical risk assessment–sufficient protection against mixture effects?. Environmental Health 2013, 12 (1), 53 10.1186/1476-069x-12-53. 23816180 PMC3708776

[ref116] Azuma K.; Uchiyama I.; Uchiyama S.; Kunugita N. Assessment of inhalation exposure to indoor air pollutants: Screening for health risks of multiple pollutants in japanese dwellings. Environ. Res. 2016, 145, 39–49. 10.1016/j.envres.2015.11.015. 26618504

[ref117] Page J.; Whaley P.; Bellingham M.; Birnbaum L. S.; Cavoski A.; Fetherston Dilke D.; Garside R.; Harrad S.; Kelly F.; Kortenkamp A.; Martin O.; Stec A.; Woolley T. A new consensus on reconciling fire safety with environmental & health impacts of chemical flame retardants. Environ. Int. 2023, 173, 107782 10.1016/j.envint.2023.107782. 36858883

[ref118] Hu Y.; Wu M.; Li Y.; Liu X. Influence of pm 1 exposure on total and cause-specific respiratory diseases: a systematic review and meta-analysis. Environ. Sci. Pollut. Res. 2022, 29 (10), 15117–15126. 10.1007/s11356-021-16536-0. PMC8810454 34628607

[ref119] Marval J.; Tronville P. Ultrafine particles: A review about their health effects, presence, generation, and measurement in indoor environments. Build. Environ. 2022, 216, 108992 10.1016/j.buildenv.2022.108992.

[ref120] Borsboom W.; De Gids W.; Logue J.; Sherman M.; Wargocki P. Tn 68: Residential Ventilation and Health, Air Infiltration and Ventilation Centre: Brussels, Belgium, 2016;.

[ref121] Parthasarathy S. Modeling indoor exposures to vocs and svocs as ventilation rates vary. Tech. Rep.; Lawrence Berkeley National Lab.(LBNL): Berkeley, CA (United States), 2012;.

[ref122] Liu C.; Zhang Y.; Benning J.; Little J. The effect of ventilation on indoor exposure to semivolatile organic compounds. Indoor air 2015, 25 (3), 285–296. 10.1111/ina.12139. 24939666

[ref123] Xu J.; Wang P.; Li T.; Shi G.; Wang M.; Huang L.; Kong S.; Gong J.; Yang W.; Wang X.; Geng C.; Han B.; Bai Z. Exposure to source-specific particulate matter and health effects: a review of epidemiological studies. Curr. Pollut. Rep. 2022, 8 (4), 569–593. 10.1007/s40726-022-00235-6.

[ref124] Sandoval Diez N. Methods to assess pm2. 5 exposure from indoor sources in epidemiological studies: a review. Tech. Rep.; Utrecht University, 2022;.

[ref125] Singer B. C.; Chan W. R.; Kim Y.-S.; Offermann F. J.; Walker I. S. Indoor air quality in california homes with code-required mechanical ventilation. Indoor air 2020, 30 (5), 885–899. 10.1111/ina.12676. 32304607

[ref126] Chan W. R.; Kim Y.-S.; Less B. D.; Singer B. C.; Walker I. S. Ventilation and indoor air quality in new california homes with gas appliances and mechanical ventilation. Tech. Rep.; Lawrence Berkeley National Lab.(LBNL): Berkeley, CA (United States), 2019;.

[ref127] Martin E.; Khan T.; Chasar D.; Sonne J.; Rosenberg S.; Antonopoulos C.; Metzger C.; Chan W. R.; Singer B.; Lubliner M. Characterization of mechanical ventilation systems in new us homes: What types of systems are out there and are they functioning as intended. 2020 Summer Study on Energy Efficiency in Buildings, 2020;.

[ref128] Zhao H.; Chan W. R.; Cohn S.; Delp W. W.; Walker I. S.; Singer B. C. Indoor air quality in new and renovated low-income apartments with mechanical ventilation and natural gas cooking in california. Indoor air 2021, 31 (3), 717–729. 10.1111/ina.12764. 33070378

[ref129] Commission C. E. 2019 Residential Compliance Manual: For the 2019 Building Energy Efficiency Standards. Title 24, Part 6, and Associated Administrative Regulations in Part 1; California Energy Commission, 2018;.

[ref130] Niculita-Hirzel H. Latest trends in pollutant accumulations at threatening levels in energy-efficient residential buildings with and without mechanical ventilation: A review. Int. J. Environ. Res. Publ. Health 2022, 19 (6), 3538 10.3390/ijerph19063538. PMC8951331 35329223

