# Supplementary material for: Harm from Residential Indoor Air Contaminants
Source: Environ Sci Technol. 2023 Dec 27;58(1):242–57. doi: 10.1021/acs.est.3c07374 (PMC10785761; doi:10.1021/acs.est.3c07374)
Supplement: Supplementary file 1 — es3c07374_si_001.pdf [file es3c07374_si_001.pdf]

Harm from residential indoor air contaminants

Giobertti Morantes[a], Benjamin Jones[a*], Constanza Molina[b], Max Sherman[a]

[a] Department of Architecture and Built Environment, University of Nottingham, Nottingham, NG7 2RD, United Kingdom

[b] Escuela de Construcción Civil, Pontificia Universidad Católica de Chile, Avenida Vicuña Mackenna 4860, Macul, Santiago, 7820436, Chile

* Corresponding author: Benjamin.Jones@nottingham.ac.uk

B. Supporting Information (Supplement B)

Summary: 37 pages, 5 figures, 8 tables, 4 matrices.

Outlook.

Extended descriptions (Section B-1 to B-6)

Supplemental Tables (1 to 8)

Matrix S1 to S4

Supplemental Figures (A to D)

37 Pages (S1 – S37)

B-1. The harm intensity. *This section provides further information about Section 2. Approach, on the main manuscript.*

We selected priority airborne contaminants commonly found in dwellings to estimate harm intensity from an existing list based on the chronic health damage they were identified to cause by Logue et al. [1]. In addition to these, we included PM_{10}, mold (spores) and radon in our analysis, as they are relevant to the global burden of disease. We also incorporated other airborne contaminants in dwellings that recent literature has considered relevant, such as 1,3-butadiene, isoprene, and trichloroethylene [2].

B-1.1 Toxicological analysis.

B-1.1.1 Approach

Huijbregts et al. [3] proposed individual carcinogenic or non-carcinogenic effect factors (EF, DALY/kg)[1] for a comprehensive list of air contaminants. Logue et al. [1] further advance Huijbregts proposal by suggesting that the addition of individual effects can derive an all-cause effect for each contaminant (i) as

$$\mathrm{EF_i} = \left(\mathrm{EF_{cancer,i}} \cdot \mathrm{ADAF}\right) + \mathrm{EF_{non-cancer,i}} \tag{1}$$

The Age Adjustment Dependent Factor (ADAF) is used to adjust the carcinogenicity effect. The effect factors are developed from Dose-Response Factors (DRF, case/kg) and Damage Factors (DF, DALY/case) as

$$\mathrm{EF_{k,i}} = \mathrm{DRF_{cancer\,|\,non-cancer,i}} \cdot \mathrm{DF_k} \tag{2}$$

Where k indicates the disease (i.e. the health effect) related to the contaminant i. The dose-response factor indicates the change in morbidity and/or mortality per unit mass intake of the contaminant i ($\mathrm{DRF_{cancer\,|\,non-cancer,i}}$) and the damage factor explains the severity of disability ($\mathrm{DF_k}$) [4, 5].

The dose-response factor is the quotient of a constant and the median effective dose that explains a carcinogenic or non-carcinogenic effect, for each contaminant i ($\mathrm{ED_{50}}_{\mathrm{cancer\,|\,non-cancer,i}}$),

$$\mathrm{DRF_{cancer\,|\,non-cancer,i}} = \frac{0.5}{\mathrm{ED_{50}}_{\mathrm{cancer\,|\,non-cancer,i}}} \tag{3}$$

The $\mathrm{ED_{50}}$ is a measure of the human-equivalent daily dose (kg) received by a person over their lifetime that produces a specific effect in 50% of a population [6-8]. They are derived from toxicological studies in animals or humans. The constant of 0.5 is a default multiplier for human carcinogenic effects

[1] Effect factors are expressed with units of DALY per mass intake of the contaminant. We use kilograms (kg) to express the units of mass, however this can be changed to denote the appropriate mass unit according to each contaminant, e.g. Radon is expressed with units of becquerels (Bq).

(expressed as cases) and assumes a linear effect with a 50% additional probability of getting cancer while inhaling an ED_{50} dose [3, 7, 9-13].

Considering a chronic intake expressed as an annual breathing rate (BR, m^3/person/year), we can derive the form of the toxicology-based harm intensities (HI, DALY/µg/m^3/person/year) as

$$\mathrm{HI}_{k,i} = \frac{1}{2} \cdot \frac{\mathrm{DF}_k \cdot \mathrm{BR}}{\mathrm{ED}_{50_{\mathrm{cancer}\,|\,\mathrm{non-cancer},i}}} \tag{4}$$

Here, equation (4) is the same presented in the main manuscript. The diseases (subscript k) that are representative of the carcinogenic or non-carcinogenic effects of each contaminant were identified following Huijbregts et al. [3] and other references on indoor airborne contaminants and their health effects [14, 15]. Finally, the sum over specific causes derives the all-cause effect as

$$\mathrm{HI}_i = \left[\left(\frac{0.5}{\mathrm{ED}_{50_{\mathrm{cancer},i}}} \cdot \mathrm{DF}_k \cdot \mathrm{ADAF}\right) + \left(\frac{0.5}{\mathrm{ED}_{50_{\mathrm{non-cancer},i}}} \cdot \mathrm{DF}_k\right)\right] \cdot \mathrm{BR} \cdot (\mathrm{N}) \tag{5}$$

With N describing the population, e.g. per 100,000 person.

B-1.1.2 Sources of data

Relevant life cycle impact assessment (LCIA) literature was reviewed to identify commonly used sources for parameter data [4, 5]. Search terms and criteria from these studies were applied to retrieve up-to-date information. Data sources were selected based on their appropriateness for each required parameter. Key references for data selection included:

- Damage factors (DF_k) were derived from the extensive databases on burden of disease and health statistics provided by the 2019 Global Burden of Disease study [16, 17].
- Dose-response factors ($\mathrm{DRF}_{\mathrm{cancer}\,|\,\mathrm{non-cacner},i}$) were derived from the UNEP-SETAC consensus model for the evaluation of comparative toxicity, known as USEtox-2019 or USEtox® 2.0 [9]. Information for Radon from [18, 19].
- Breathing rate (BR). We used 14.80 m^3 per person per day for adults 16-81+ years for the breathing rate [20, 21].
- Age-dependent adjustment factor (ADAF). The ADAF is reported in the literature for the estimation of cancer risks as i) a 10-fold ADAF exposures before 2 years of age, ii) a 3-fold ADAF for exposures between 2 and <16 years of and iii) ADAF equals 1 for exposures after turning 16 years of age [22, 23].

Data sources were carefully selected to align with current knowledge on each parameter within the health impact framework. Literature was prioritized based on frequency of citation and recognition as authoritative in the field. This aimed to enhance the reliability and validity of the analysis.

B-1.1.3 Intermediate steps

To enhance the clarity surrounding the calculations of median HI, we include intermediary steps to provide a more comprehensive understanding of how these values are derived, using one recognizible contaminant as example.

Matrix S1. Intermediary Steps for Tox-HI

Formaldehyde (HCHO)	Parameters’ central tendency and uncertainty descriptors from sources of data					
	DRF_{cancer} (GSD^2)	$DRF_{non-cancer}$ (GSD^2)	$DF_{Leukemia}$ (95%CI)	DF_{asthma} (95%CI)	ADAF (95%CI)	BR (95%CI)
	1.06 (4)	0.01 (18)	35 (29 – 42)	0.59 (0.44 – 0.77)	1 (1.6 - 10)	5402 (4928 - 5913)
Units	case/kg		DALY/case		none	m^3/person/year

Monte Carlo approach gives:

Matrix S2. Example applying intermediary steps for Tox-HI

HCHO	Parameters’ Median (output of MC approach)						
	DRF_{cancer}	$DRF_{non-cancer}$	$DF_{leukemia}$	DF_{asthma}	ADAF	BR	Factor kg - µg
	0.598	0.00111	35.1	0.59	0.62	5389	10^9

Given the properties of the parameter, using the inputs’ medians gives the output median:

$$\mathrm{HI}_{HCHO} = [(0.598 \cdot 35.1 \cdot 0.62) + (0.00111 \cdot 0.59)] \cdot 5389 \cdot \frac{10^5}{10^9} = 7.1$$

The Monte Carlo approach yields a GSD for the harm intensity. However, comprehensive details and discussions regarding this methodology are beyond the scope of this work. We anticipate that a complete paper dedicated to explaining the tox-harm approach will provide a more in-depth exploration.

B-1.1.4 Toxicology-based outcomes

Harm intensities based on the revised toxicology research were calculated for 39 contaminants commonly found in dwellings. Median harm intensities per 10^5 population, uncertainty estimates expressed through the Geometric Standard Deviation (GSD) and the best estimate for all-cause effect are shown in

Supplemental Table *1*.

Supplemental Table 1. Toxicology-based all-cause Harm Intensities (HI_i).

Contaminant[2]	Median (10^5)	GSD	Best estimate of all-cause
Acetaldehyde	0.053	4.8	Lung Cancer & Non-Cancer Average
Acrolein	1.3	8.5	Asthma
Acrylonitrile	1.2	4.1	Lung Cancer & Non-Cancer Average
Benzene	0.18	4.4	Leukaemia & Non-Cancer Average
Benzyl chloride	0.062	11	Stomach Cancer
1,3-Butadiene	0.27	3.9	Cancer Average & Non-Cancer Average
2-Butoxyethanol	0.01	8.7	Liver Cancer & Non-Cancer Average
Cadmium Cd(II)	5.3	8.9	Lung Cancer & Non-Cancer Average
Carbon disulfide	0.29	1.1	Non-Cancer Average
Carbon tetrachloride	0.52	7.3	Liver Cancer & Non-Cancer Average
Chloromethane	0.00027	10	Non-Cancer Average
Chromium Cr(VI)	17	15	Lung Cancer & Non-Cancer Average
Crotonaldehyde(trans)	1.1	7.2	Liver Cancer
1,2-Dibromoethane	3.4	5.8	Liver Cancer & Non-Cancer Average
1,4-Dichlorobenzene	0.012	6.4	Cancer Average & Non-Cancer Average
1,2-Dichloroethane	0.052	5.4	Stomach Cancer
1,1-Dichloroethene	0.15	6.1	Lung Cancer & Non-Cancer Average
Ethanol	0.0005	5.8	Liver Cancer
2-Ethylhexanol	0.0029	8.4	Liver Cancer
Formaldehyde	7.1	5.4	Leukaemia & Asthma
Hexachlorobutadiene	0.03	4.8	Cancer Average &
Hexane	0.0018	8.7	Cancer Average & Non-Cancer Average
Isoprene	0.0092	7.0	Cancer Average
Limonene (d-...)	0.0093	6.5	Cancer Average
2-Methoxyethanol	0.0028	7.8	Non-Cancer Average
Methyl methacrylate	0.051	2.8	Non-Cancer Average
Methyl tert-butyl ether	0.026	4.6	Leukaemia & Non-Cancer Average
Methylene chloride	0.01	5.6	Breast Cancer & Non-Cancer Average
Naphthalene	0.36	5.9	Lung Cancer & Non-Cancer Average
Ozone	2.6	6.2	Lung Cancer
Radon[3]	0.37	3.7	Lung Cancer
Styrene	0.11	4.7	Breast Cancer & Non-Cancer Average
1,1,2,2-Tetrachloroethane	0.13	6.2	Liver Cancer
Tetrachloroethene	0.052	6.2	Leukaemia & Non-Cancer Average
Toluene	0.00087	5.4	Non-Cancer Average
1,1,2-Trichloroethane	0.15	5.7	Liver Cancer & Non-Cancer Average
Trichloroethylene	0.0035	5.1	Cancer Average
Vinyl chloride	0.98	5.4	Liver Cancer & Non-Cancer Average
Xylenes	0.0034	6.1	Mouth And Oropharynx Cancer & Non-Cancer Average

[2] DALY/µg/m^3/person/year

[3] DALY/Bq/m^3/person/year. Bq, Becquerels

B-1.2 Epidemiological analysis.

B-1.2.1 Approach

The attributable harm caused by a disease (k) from exposure to a contaminant (i) ($\mathrm{Harm}_{k,i}$, DALYs/person/year) is a function of the baseline disease incidence (γ_{0_k}, cases/person/year), the damage factor (DF_k, DALY/case), a risk related empirical parameter beta ($\beta_{k,i}$, change/μg/m^3) and the contaminant exposure concentration[4] (C_i, μg/m^3), associated through a saturation expression using a non-linear relationship as

$$\mathrm{Harm}_{k,i} = \gamma_{0_k} \cdot \left(1 - e^{-\beta_{k,i} \cdot C_i}\right) \cdot \mathrm{DF}_k \qquad (6)$$

Important outlines from this expression are:

- ✓ In general, this analysis follows the Comparative Risk Assessment (CRA) conceptual framework [24].
- ✓ This expression is equivalent to that used in the Global Burden of Disease (GBD) studies to quantify the environmental burden of disease attributable to wide variate risk factors, as well as another similar research [1, 5, 16, 25].
- ✓ Damage factors express the relationship between the cases of mortality or morbidity attributed to a contaminant and the corresponding harm (as DALYs/person/year). These factors are typically expressed in terms of DALYs/case, where the "case" in the denominator refers to the baseline disease incidence (γ_{0_k}, case/person/year) [26-29].
- ✓ The term in parenthesis is known as the population attributable fraction in LCIA [28, 29].
- ✓ The environmental burden of disease for health outcomes presented in the GBD (as DALYs/person/year) is the product of the damage factor and the baseline disease incidence.
- ✓ This log-linear model is the most widely function used for health impact assessment [30, 31].
- ✓ The approach assumes there is no threshold concentration below which effects aren't seen (see **Section B-6B-6. Shape of the curve and regime of concentrations.** Here, details regarding the relationship between the shape of the concentration – harm curves. for further discussion)
- ✓ The shape of the curve is a function of the exponent (the curve that is generated by the expression is a sigmoid curve, which has a steep linear initial slope that then flattens out as values increase). When the equation is evaluated at the low concentrations normally expected in dwellings [32, 33], a linear concentration-response association is often assumed appropriate

[4] mass can be substituted with other quantitative units, such as Bq (becquerels) for radon or CFU (colony-forming units) for mold.

[27, 28, 34] (see **Section B-6B-6. Shape of the curve and regime of concentrations.** Here, details regarding the relationship between the shape of the concentration – harm curves. for further discussion), and expressed as

$$1 - e^{-\beta_{k,i} \cdot C_i} \approx \beta_{k,i} \cdot C_i \tag{7}$$

The expression of harm can therefore be described as,

$$\mathrm{Harm}_{k,i} = \gamma_{0_k} \cdot \beta_{k,i} \cdot C_i \cdot DF_k \tag{8}$$

Dividing for the concentration will derive the harm intensity (HI, DALY/µg/m^3/person/year) as,

$$HI_{k,i} = \gamma_{0_k} \cdot \beta_{k,i} \cdot DF_k \tag{9}$$

In epidemiological studies, the subscript k representing all major diseases (considering both morbidity and mortality) is often represented by all-cause mortality risk estimates that encompass all causes of death from long-term exposure-related chronic diseases and deaths hastened by recent exposure to air pollution [35]. When this information is not available, different diseases can be summed to approximate the all-cause effect [27-29].

The diseases (subscript k) that are representative of the all-cause effect were identified reviewing and following the most current epidemiological evidence and advice for each contaminant [16, 17, 35-42]. Finally, the sum over specific causes derives the all-cause effect as,

1. When the risk estimate is given for specific causes:

$$HI_i = (N) \cdot \sum_k y_{0_k} \cdot \beta_{k,i} \cdot DF_k \tag{10}$$

2. When the risk estimate is given for all-cause mortality:

$$HI_i = (N) \cdot \beta_i \sum_k y_{0_k} \cdot DF_k \tag{11}$$

With N describing the population, e.g. per 100,000 person.

B-1.2.2 Sources of data

Relevant life cycle impact assessment (LCIA), health risk assessment, and comparative risk assessment literature was reviewed to identify commonly used data sources [4, 5, 16, 43, 44]. Search terms and criteria from these studies were applied to retrieve current information. Data sources were selected based on their appropriateness for each required parameter. Key references that guided data selection included:

- We conducted a literature review of studies published between 2010-2020 and other literature that compiled or reviewed risk estimates to obtain the beta parameter ($\beta_{k,i}$) for risk derivation. We base our approach on individual pollutant risk estimates, driven by their availability, while acknowledging the emerging potential of multipollutant regressions in epidemiological exposure assessment studies [35, 40, 45].
- The GBD Collaborative Network provided us with estimates for the disease-specific baseline incidence rates (γ_{0_k}) and damage factors (DF_k) for the year 2019 for the target health effects (k) identified through the risk estimates ($\beta_{k,i}$) for the global population of all ages and both sexes [16, 17].

Data was sourced and reviewed through a comprehensive process based on relevant literature and specific selection criteria. This aimed to establish a strong foundation for the health impact methodology. The data used in the analysis was identified from authoritative sources, meeting standards for quality and relevance.

B-1.2.3 Intermediate steps

To enhance median HI calculations, we include intermediary steps for a comprehensive understanding of how values are derived, using a prominent contaminant as an example.

Matrix S3. Intermediary Steps for Epi-HI

$PM_{2.5}$	Parameters’ central tendency and uncertainty descriptors from sources of data		
	$\beta_{\text{all-cause mortality}}$ (95%CI)	$DF_{\text{all-cause mortality}}$ (95%CI)	$\gamma_{0\,\text{all-cause mortality}}$ (95%CI)
	0.008 (0.006 – 0.009)	12 (10 - 14)	0.00675 (0.00639 – 0.00715)
Units	Change/μg/m^3	DALY/case	case/person/year

Monte Carlo approach gives:

Matrix S4. Example applying intermediary steps for Epi-HI

$PM_{2.5}$	Parameters’ Median (output of MC approach)		
	$\beta_{\text{all-cause mortality}}$ (95%CI)	$DF_{\text{all-cause mortality}}$ (95%CI)	$\gamma_{0\,\text{all-cause mortality}}$ (95%CI)
	0.008	11.6	0.00675

Given the properties of the parameter, using the inputs’ medians gives the output median:

$$HI_{PM_{2.5}} = 0.008 \cdot 11.6 \cdot 0.00675 \cdot 10^5 = 60$$

The Monte Carlo approach yields a GSD for the harm intensity. Comprehensive details and discussions about this methodology exceed the scope of this study. We anticipate that a dedicated paper elucidating the epi-harm approach will offer a more thorough exploration.

B-1.2.4 Epidemiology-based outcomes

We related harm to exposure via harm intensities derived from epidemiology research for ten common indoor airborne contaminants. Median harm intensities per 10^5 population, uncertainty estimates expressed through the Geometric Standard Deviation (GSD) and the best estimate for all-cause effect are shown in Supplemental Table 2.

Supplemental Table 2. Epidemiology-based all-cause Harm Intensities (HI_i).

**Contaminant**[5]	**Median (10^5)**	**GSD**	**Best estimate of all-cause**
Acrolein	1.2	5.5	Asthma morbidity
Benzene	0.062	1.4	Leukaemia mortality
Formaldehyde	4.0	2.2	All-cause (LC, Lk mortality; asthma morbidity)
Mold[6]	0.027	2.2	Asthma morbidity
Nitrogen Dioxide	5.6	1.7	All-cause mortality (COPD, LRI, URI, LC)
Ozone	1.3	1.9	All-cause mortality (COPD, LC)
PM_{10}	30	1.3	All-cause mortality (IHD, DM, Str, COPD, LRI, LC)
$PM_{2.5}$	60	1.2	All-cause mortality (IHD, DM, Str, COPD, LRI, LC)
Radon[7]	0.45	1.7	Lung Cancer mortality
Sulphur Dioxide	1.9	4.5	All-cause mortality (COPD)

Abbreviations. LC: Lung Cancer; Lk: Leukaemia; COPD: Chronic Obstructive Pulmonary Disease; LRI: Lower Respiratory Infections; URI: Upper Respiratory Infections; IHD: Ischaemic Heart Disease; DM: Diabetes Mellitus; Str: Stroke.

[5] DALY/μg/m^3/person/year

[6] DALY/CFU/m^3/person/year. CFU, colony-forming units. As mold spores of the genus Cladosporium

[7] DALY/Bq/m^3/person/year. Bq, Becquerels

B-2. Further model details. *This section provides further information about Section 3.4 Parameter Distributions, of the main paper.*

The harm intensities (HI_i) and concentrations (C_i) can have more than one available estimate of their central tendency estimate, and so they need to be combined, or pooled [46] to produce a single value. We performed meta-analyses [47] using a random-effects model that follows a maximum likelihood approach. This assumes that all studies represent a random sample of all possible results that account for heterogeneity between studies. The meta-analyses were performed using the "metan" commands of the statistical software STATA 17.0, which applies the DerSimonian and Laird random effects estimators for pooling estimates [48-50].

The meta-analysis technique was chosen over other available approaches to pooled results of contaminant concentrations [32, 51-53] because the inputs and outputs of a meta-analysis account for the uncertainty in the measurements via the 95% CI of the distribution of the sampled concentrations, where as other approaches are based on the numbers of samples collected or sampling sites.

Concentrations are assumed to be right-skewed and lognormally distributed, as commonly accepted in the field [54-57]. The harm intensities were also assumed to be lognormally distributed. A lognormal probability density function (PDF) fit is plausible because the harm intensity parameter is a positive definite distributed random variables, it is physically impossible for it to have negative values, furthermore the central limit theorem states that the product of many independent random variables will be lognormally distributed [3, 58, 59].

A Monte Carlo approach was used to model distributions for the input (HI_i and C_i) and output ($Harm_i$) parameters [60]. The process is repeated for at least 100,000 simulations or until the means of the output parameter are normally distributed, which represents the convergence criterion. Descriptive statistics of the inputs and outputs were obtained (mean, median, standard deviation, Geometric Standard Deviation -GSD, Squared Geometric Standard Deviation -GSD^2). A MATLAB code was designed to such end.

Medians are chosen as the estimate of central tendency to interpret the results for all parameters. The median is generally considered a more representative central tendency measure than the mean for non-normal distributions. The uncertainty is described trough the GSD.

When the shape of the PDF is lognormal and the 95% CI are known, an uncertainty factor to describe uncertainty in the parameters can be approximated to the GSD^2 [59], as

$$\mathrm{GSD}^2 = \sqrt{\frac{97.5\ \text{percentile}}{2.5\ \text{percentile}}} \quad (12)$$

The equation (12) was applied to extract an uncertainty estimate from the descriptive statistics given in revised references whenever necessary.

Medians are chosen as the estimate of central tendency to interpret the results for all parameters. The median is generally considered a more representative central tendency measure than the mean for non-normal distributions.

Uncertainty is defined here as the geometric standard deviation (GSD), fitting the assumed lognormal distribution for parameters. The GSD directly measures distribution spread linearly. Squaring the GSD approximates the 95% confidence interval for a lognormal distribution, allowing direct probability distribution construction and confidence interval calculation. For the intended uses, GSD suitably represents uncertainty because: Real-world data often does not perfectly fit assumed distributions, especially in the tails where true bounds are uncertain; The analyses do not rely on the extreme 2.5th or 97.5th percentiles; GSD provides a straightforward lognormal spread measure without claiming specific bounds; GSD prevents potential scaling effects when combining variance-based measures.

B-3. A systematic review to quantify representative concentrations in dwellings. *This section provides further information about Sections 2.3, 3.2 and 4.1 of the main paper.*

B-3.1 Methodology

A search strategy was defined to find measurement data in dwellings for the 44 airborne contaminants with a harm intensity estimate. Publications in any language were searched if at least an English-language abstract was available in the databases. A requirement for mentions of measurement (and synonyms) was included to decrease results only reporting modeling, policy perspectives, or commentary on the topic. The search criteria are listed in Supplemental Table 3 without consideration for the specific syntax required of the databases.

Supplemental Table 3. Keywords and Boolean operators when performing systematic reviews on input parameters.

**Input parameter**	Representative Indoor Air Contaminant Concentration (C_i)
**Research question**	What are the values of indoor air pollutants in households?
**Keywords and Boolean operators**[+]	(house* OR domestic OR dwelling*) AND "indoor air" AND ("air pollution" OR "air quality" OR "particulate matter" OR "Nitrogen Dioxide" OR NO2 OR Ozone OR O3 OR "Sulphur Dioxide" OR SO2 OR ("Carbon Monoxide" NOT poison*)) AND (review OR "observational stud*") AND (exposure OR monitor* OR sampl* OR measure*) AND (concentrat*) AND (mean OR median) AND (sample*)
**Databases**	Scopus, Pubmed, and Web of Science.

[+] All 44 contaminants followed the same logic, with each one as a keyword.

The search was limited to residential environments (including community residences, dormitories, apartments/flats and houses in general) and to studies published between 2010-2021, after the

publication of Logue's work [32]. We expanded the search limits and included studies published prior to 2010 if there were no studies available for the contaminant within the time frame initially chosen. The search was complemented by other reviews on indoor exposures to the contaminants of interest in dwellings [33]. We also considered technical reports from recognised international organizations mentioned in these studies. We included concentrations measured by fixed or portable samplers or monitors (both optical or gravimetric samplers). All countries and regions were included. The review considered the contaminants regardless of source therefore, results will include contaminants emitted purely from an indoor source, others that enter predominantly from outdoors, and some having both. The results of this process of literature search and publication review are shown in Supplemental Figure 1.

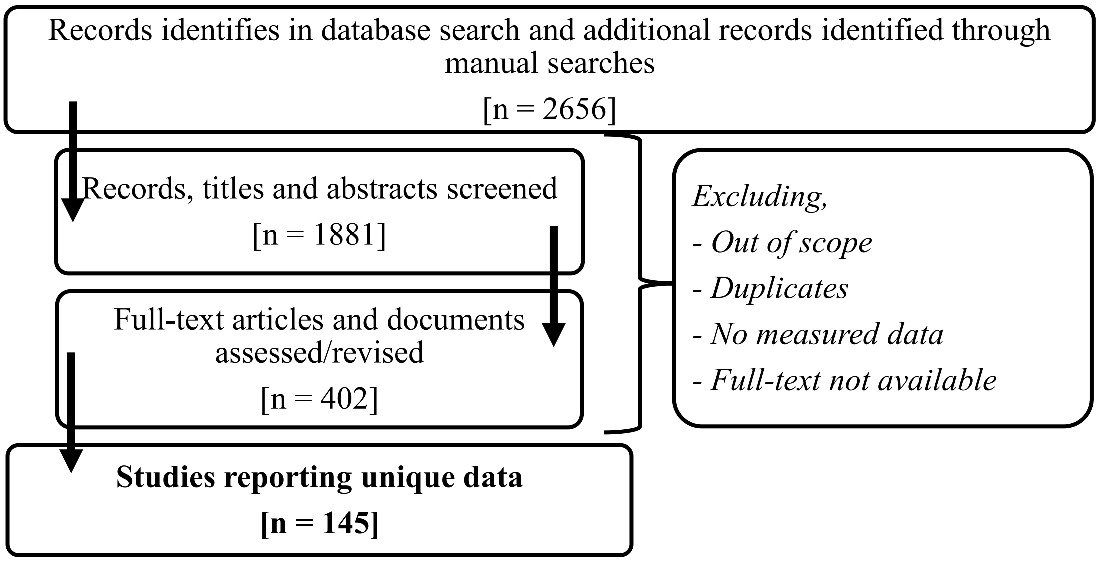

Supplemental Figure 1. Review process diagram.

The search strategy specifically targeted dwellings under typical, real-world usage conditions . Studies focused solely on punctual indoor activities like cooking or cleaning, and atypical dwellings like passive houses, were excluded as potentially skewing indoor contaminant concentration representation. For example, passive house works were not considered [61], and papers addressing specific activities, like O'Leary et al. [62] on $PM_{2.5}$ from cooking, underwent individual assessment for inclusion. The aim was encompassing everyday living exposures in mainstream housing to achieve generalizable concentration summaries. Highly controlled or constrained scenarios were avoided to prevent biasing away from real-world central tendencies.

B-3.2 Results

A relatively high number of studies from the USA, China, Canada, and UK were found. Very sparse studies were reported for Africa, Latin America, and the Middle East. The United States was the most intensely studied country, with 37 publications and 166 of the datasets; followed by China, with 20 publications and 104 of the datasets; and Canada, with 12 publications and 95 of the datasets. Although we followed a world-level perspective, several regions have not been accounted for in this review, which does not reflect that these regions of the world have been sampled sparsely, rather it could be an effect of the search strategies by not including studies in all languages. It reflects representative indoor concentration data of Global North countries (these are regions with reasonable data homogeneity and representation). We included four references from before 2010 due to their relevance in measuring selected contaminants such as cadmium, chromium, and the Cladosporium genus for mold, for which little data was found within the initial time frame of 2010-2020 [63] [64-66]. Finally, we considered one relevant report by Health Canada regarding acrolein [67]. **Error! Reference source not found.** details a full list of the extracted information with summary statistics of the studies included, and the results of the meta-analyses performed for the datasets of each contaminant (the latter were the inputs for the Monte Carlo approach).

Supplemental Table 4. Results from the systematic review and meta-analysis on indoor air contaminant concentration measurements in dwellings.

B-3.3 Discussion

Review articles have been conducted on airborne contaminants present in indoor residential environments. However, these studies vary in their objectives, geographical scope, types of contaminants, underlying methodology decisions, and time periods considered. The central tendency and statistics reported in each study varies, which insert difficulty when making a direct comparison. Additionally, certain review articles employ a narrative approach, without pooling data in a quantitative manner. Supplemental Table 5 summarizes details on existing literature reviews of indoor contaminants in dwellings.

Supplemental Table 5. Summary of existing literature and reviews

Work	Overall contams. Surveyed	Statistics extracted	Spatial scope	Method of pooling	Time frame	# of Refs	Indoor
This work	44 (see main text for list)<br>PM<br>Criteria pollutants<br>[S,V]VOCs<br>Biological<br>Radiological	Mean, median, SD, 95%C.I.	Global	Meta-analysis	2010-2020	145	Any residential
Logue et al. (2011) [32]	267 chemical pollutants:<br>PM<br>Criteria pollutants<br>[S,V]VOCs	Median, 25thpctile, 95thpctile.	USA and similar countries	Weighted by number of measurements within each study	1995–2010	77	Residences
Vardoulakis et al. (2020) [33]	24 selected pollutants:<br>PM<br>Criteria pollutants<br>[S,V]VOCs	Minimum, maximum	No limitations	None	2000-2017	141	Households
Fazli et al. (2018) [68]	10 pollutants:<br>PM<br>Criteria pollutants<br>VOCs	Median	USA	None	--	72	Houses
Morawska et al. (2013) [69]	PM fractions only	Median, minumum, maximum	Developed countries	None	January 1989 and October 2012	44	Residences, personal and schools
Morawska et al. (2017) [51]		Weighted mean, minimum, maximum	No limitations	Weighing the number of individual locations in each study	1990–2017	12	Homes, schools and day cares, offices, and aged care facilities
Ilacqua et al. (2022) [52]		Median, minumum, maximum	Worldwide	Mean weighted by study size.	1990-2019	538	Residential
Nishihama et al. (2021) [70]		Median, minumum, maximum	Japan	None	2011-2016	--	Households
Ye et al. (2017) [71]	23 pollutants<br>PM<br>Criteria pollutants<br>[S,V]VOCs<br>Biological<br>Radiological	Minimum, maximum	China (separated by rural and urban)	None	last 10 years (assume 2006-2016)	11	Residential
Halios et al. (2022) [53]	65 individual VOCs	WAGM, minimum, maximum	Europe	Weighted Average Geometric Mean (WAGM)	2000-2020	39	Residences

Adopting a non-spatially-restricted perspective has both advantages and limitations. To provide insights on data variability, PM_{10} and $PM_{2.5}$ are examined for leading study regions: the USA, China, Canada, and the UK. For $PM_{2.5}$, these countries provided: USA 24%, China 22%, Canada 6%, UK 5% of studies. Meta-analysis by country gives concentrations (95%CI) of: USA 14 (11-18) µg/m^3; China

73 (52-103) μg/m^3; Canada 3 (2-5) μg/m^3; UK 25 (14-45) μg/m^3. The studies included for China reflect the use of solid fuels (wood and coal) in homes, influencing a relatively higher central estimate. The USA estimate aligns well with reported values for that country. The USA and China hold 25% and 23% weight in the $PM_{2.5}$ pooled concentration. Supplemental Table 4 shows the $PM_{2.5}$ pooled concentrations. For PM_{10}: USA 3%, China 17%, UK 9% of studies. By country: USA 20 (5-79) μg/m^3; China 171 (112-261) μg/m^3; UK 42 (25-71) μg/m^3. China has 21% PM_{10} pooled concentration weight. Supplemental Table 4 shows the PM_{10} pooled concentrations. $PM_{10\text{-}2.5}$ fractions ranged 0.43-0.70, reflecting literature variability in coarse versus fine fraction dominance (see main manuscript for results on this topic). Supplemental Figure C and Supplemental Figure C. Forest plots for meta-analysis of

PM$_{2.5}$, sub-group by country or region.

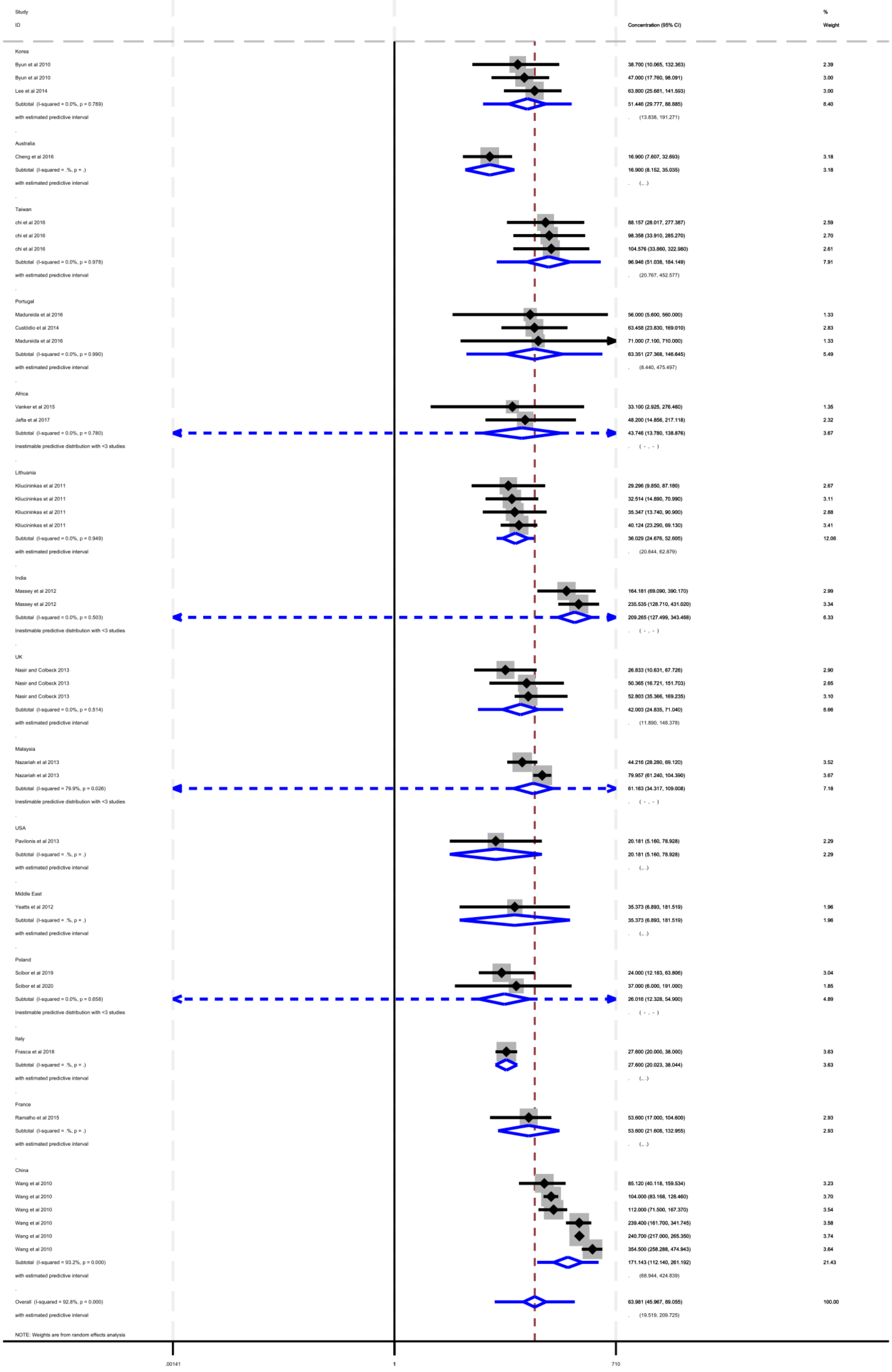

Representative indoor air concentration: PM10 -Subgroup Countryofstudy

Supplemental Figure D show the forest plots for the meta-analyses of these contaminants.

Source attributions, including appliances, fuel types, and personal activities, were reported by the revised papers during the sampling periods. Recent works provide additional insights on source attribution and their influence on indoor concentrations [33] [51] [53]. To investigate our findings, $PM_{2.5}$ influencing sources were extracted from included studies. Seven prominent sources were identified and the frequency each one was linked to $PM_{2.5}$: Combustion [candles, incense, wood-or-coal for cooking, wood-or-coal for heating, smoking] - 52%; Cleaning - 9%; Occupant activities - 9%; Outdoor - 9%; Personal care products - 8%; Pets - 3%; Interior modifications - 11%. As expected, everyday combustion activities within homes were the primary $PM_{2.5}$ source.

All sampler types were included, excluding only modelled concentrations. Gravimetric equivalency was not addressed (for more see [72-74]). This represents an opportunity for further discussion, as previous PM reviews do not address this issue. For $PM_{2.5}$ studies, 3/5 used gravimetric and 2/5 used optical samplers. Pooled values by sampler were: -Optical: 22 $\mu g/m^3$ (95% CI 14 - 34); -Gravimetric: 29 $\mu g/m^3$ (95% CI 19 - 45). Although differences exist, statistical significance was not explored, as examining sampler influence on harm differences was outside the current scope.

Overall, the concentration distribution for the contaminants reflect exposures under typical usage conditions, encompassing cooking, candle use, smoking, use of solid fuels (wood and/or coal), incense burning, and alike activities expected to occur in homes. However, it's important to note that in dwellings with unusually high sources of any contaminant, one should anticipate greater harm (see Section 2, equation 1 in main manuscript).

While indoor contaminant concentrations certainly vary across diverse factors like country, source, and season, the depth of concentration analysis provided sufficiently achieves this paper's aim of a harm-based analysis and its applicability to select contaminants of concern and a harm budget for dwellings. The pooled meta-analyses incorporating available global data are appropriate given this focus. We prioritized conveying the fundamental principles of the concentration systematic review to ensure a clear understanding of the approach. Further dedicated analyses – such as country/contaminant breakdowns, source attributions, seasonal sensitivities – would provide useful insights but are beyond the current scope. As the presented information indicates, indoor concentration research is vast and complex, warranting focused studies for in-depth assessment. A complete dedicated paper, akin current works in Supplemental Table 5. Summary of existing literature and reviews, will provide the remaining needs.

B-4. Estimating harm from the coarse fraction of particulate matter.

The coarse fraction of PM_{10} (referred to as thoracic coarse particles or $PM_{10-2.5}$) are, in regulatory terms, particles with an upper 50% cut-point of 10 µm aerodynamic diameter and a lower 50% cut-point of 2.5 µm aerodynamic diameter.

The all-cause harm attributable to the coarse fraction of particulate matter ($PM_{10-2.5}$) was estimated by calculating the difference between the harm due to PM_{10} and $PM_{2.5}$ as

$$\mathrm{Harm}_{\mathrm{PM}_{10-2.5}} = \mathrm{Harm}_{\mathrm{PM}_{10}} - \mathrm{Harm}_{\mathrm{PM}_{2.5}} \tag{13}$$

The harm intensity for the coarse fraction was estimated once the harm from each fraction was quantified and once the concentrations of the other PM fractions were known, as

$$\mathrm{HI}_{\mathrm{PM}_{\mathrm{coarse}}} = \frac{\mathrm{Harm}_{\mathrm{PM}_{10-2.5}}}{\mathrm{C}_{\mathrm{PM}_{10-2.5}}} \tag{14}$$

Median harm intensities per 10^5 population, uncertainty estimates expressed through the Geometric Standard Deviation (GSD) and the best estimate for all-cause effect are shown in Supplemental Table 6.

Supplemental Table 6. Epidemiology-based pseudo all-cause Harm Intensity for the coarse fraction (HI_i).

**Contaminant**[8]	**Median (10^5)**	**GSD**	**Best estimate of all-cause**
$PM_{10-2.5}$	3.8	4.3	Pseudo All-cause mortality (IHD, DM, Str, COPD, LRI, LC)

Abbreviations. LC: Lung Cancer; COPD: Chronic Obstructive Pulmonary Disease; LRI: Lower Respiratory Infections; IHD: Ischaemic Heart Disease; DM: Diabetes Mellitus; Str: Stroke.

In our analysis, the harm from PM_{10} that is not covered by $PM_{2.5}$ is explained by the coarse fraction. It suggests that there is an effect on all-cause mortality from chronic exposure to this fraction. While there are still limitations and uncertainties in the health evidence base for long-term $PM_{10-2.5}$ exposure and all-cause or cause specific mortality, epidemiologic studies reporting positive associations suggest there is a relationship between long-term exposure to $PM_{10-2.5}$ and all-cause mortality (specifically cardiovascular and respiratory morbidity and metabolic disease) [36].

Guidelines are still proposed based on PM_{10} exposures to continue to provide protection against effects associated with chronic exposure to thoracic coarse particles ($PM_{10-2.5}$). Considering the harm attributable to the coarse fraction helps to highlight that $PM_{2.5}$ is the main driver of the health burden from particulate matter [35, 40].

A lack of specific test methods or epidemiology for $PM_{2.5-10}$ exists. PM_{10} comprises $PM_{2.5}$ + $PM_{2.5-10}$. We calculated $PM_{2.5}$ and PM_{10} harm intensities (HIs) based on specific evidence per PM size. Assuming additive harm extends across sizes, $PM_{2.5-10}$ HI gets determined. This relies on additive harm across

[8] DALY/µg/m^3/person/year

fractions and no nonlinear interactions, reasonable but requiring verification. A caveat is, while no direct $PM_{2.5-10}$ epidemiology exists currently, estimation is possible by subtracting $PM_{2.5}$ contributions to total PM_{10} harm, providing initial insights pending targeted $PM_{2.5-10}$ research.

B-5. Expanding on the chronic harm of air contaminants in dwellings.

B-5.1 A preliminary assessment using DALYs to express chronic harm in dwellings.

In response to the need for an updated and refined methodology to assess the impact of airborne contaminants on human health, our work has evolved from the preliminary study [75] to the current paper. This evolution was driven by the need to address discrepancies in data interpretation between Logue et al. [1] and LCIA or comparative risk assessment, specifically related to the baseline incidence of disease and the damage factors that were derived from disparate sources of data introducing uncertainties and a lack of homogeneity in the data sources. Additionally, we address the issues regarding the dependency of one system of equations used in the previous toxicology approach on the epidemiology approach; consequently, modifications were made to our analysis.

Our current paper represents a significant improvement over the preliminary work. We have incorporated updated data sources, such as the global burden of disease database, which better align with our analysis requirements concerning the baseline incidence of disease and the damage factors. Additionally, we have transitioned from a log-linear to a linear framework, introducing the concept of the harm intensity metric, which consolidates epidemiology and toxicology data as a constant through the regime of concentrations expected in dwellings. This shift has allowed us to move away from the dependence on effect factors (DALY per kg) as the main metric for relating exposure to harm.

The consistency between the conclusions of our current study and the previous work conducted using a different method validates the linearity assumption and strengthens the reliability of our results. Furthermore, through the evolution of our work, we have developed the concept of a harm budget, and finalised the identification of contaminants of concern.

B-5.2 Further discussion addressing chronic harm in dwellings.

We reviewed the literature to assess the estimation of harm caused by indoor air contaminants (IAC) in dwellings, specifically focusing on Disability-Adjusted Life-Years (DALYs). This topic is relatively understudied, with only five studies published after 2010, in addition to the Global Burden of Disease (GBD) estimates. Our analysis, complemented with details in **Error! Reference source not found.**, revealed that Logue et al.'s seminal work [1] was used as a basis for two other studies conducted in the

USA. Three studies took a global perspective and used a Comparative Risk Assessment (CRA) method with a population attributable fraction, which is widely employed in GBD studies.

Overall, our analysis revealed similar central tendency estimates for most contaminants, with overlapping variability across the results: comparing to the studies in **Error! Reference source not found.**, our median estimates of harm are higher for 19 contaminants (PM_{10}, $PM_{2.5}$, formaldehyde, nitrogen dioxide, radon, ozone, sulphur dioxide, acrylonitrile, naphthalene, benzene, limonene (d-...), 1,3-butadiene, carbon disulfide, vinyl chloride, methyl tert-butyl ether, hexachlorobutadiene, 1,1,2-trichloroethane, 2-butoxyethanol, and 2-ethylhexanol). Our estimate median estimate of harm is lower for 12 contaminants (acrolein, 1,1-dichloroethene, chromium Cr(VI), xylenes, toluene, methylene chloride, methyl methacrylate, hexane, chloromethane, 2-methoxyethanol, and mold). Our median estimate of harm is similar for 11 contaminants (crotonaldehyde(trans), acetaldehyde, carbon tetrachloride, styrene, 1,2-dibromoethane, ethanol, cadmium Cd(II), 1,2-dichloroethane, tetrachloroethene, benzyl chloride, and 1,1,2,2-tetrachloroethane) and 1,4-dichlorobenzene is bound by existing values.

Although the findings of these studies are comparable due to the use of the DALY, the underling differences followed by each author will lead to uncertainty when comparing the results. Various factors contribute to the overall variability observed across the different references, including the choice of concentration-response function, utilization of different health outcomes, reporting various central tendency metrics, spatial and population resolution variations, geographic scope covered, differences in concentration estimates, and variations in the methodological frameworks followed.

Some contaminants appear to have the biggest differences (little-to-none overlapping in GSD range covered): for nitrogen dioxide and sulphur dioxide we found a higher estimate of harm because of the all-cause effect associated with them in our work is two to three orders of magnitude above previously hospital admission risk and damage estimates used, and acrolein falls lower in the harm ranking because the representative concentration found in our review is lower than the one used in prior works.

To analyze disparities in prior $PM_{2.5}$ harm assessments, we investigated relative influences of individual parameters. Using U.S. residential indoor $PM_{2.5}$ concentrations from Logue et al. (with a 70% time-weighting factor) led to a 59% reduction in estimated harm. Incorporating concentration data from Fazli et al. further decreased harm by 71%, highlighting the impact of lower $PM_{2.5}$ exposure levels in earlier works. In contrast, using previous risk estimates yielded a 30% reduction in harm, reflecting a smaller yet significant effect. The heightened concentration influence stems from lower U.S. median values and time-weighting consideration.

The variability (i.e the GSD) in our estimates is narrower than in Logue et al.'s [1]. Logue et al. reported large uncertainties in harm estimates due to assumptions made in quantifying uncertainty. For

epidemiology-based factors, damage factor ranges for $PM_{2.5}$ and ozone mortality were set to broadly span literature values. For toxicology, Logue et al. relied on uncertainties from Huijbregts et al. [3], where interspecies conversion, effect conversion, and non-cancer damage factors dominated. Our work aimed to reduce uncertainties by leveraging improved health data. Damage factors were drawn directly from the 2019 Global Burden of Disease study rather than arbitrarily wide literature ranges [16, 17]. For toxicology, dedicated studies were consulted that report reduced uncertainty factors reflecting increasing certainty in animal-to-human extrapolation, effect conversion, and non-cancer damage quantification [76, 77]. Furthermore, health effects data, as exemplified by $PM_{2.5}$, has improved in both robustness and precision over time. Regarding robustness, EPA Integrated Science Assessments between 2009-2019 increasingly classified $PM_{2.5}$ exposure as having a "causal relationship" with mortality/morbidity, reflecting growing strength of evidence. For precision, the $PM_{2.5}$ mortality risk estimate used by Logue et al. from earlier epidemiology had an uncertainty factor of 1.027, while the WHO estimate used here has a reduced uncertainty factor of 1.014, demonstrating increased precision (uncertainty as equation (12)).

The findings of all studies considered are in Supplemental Figure A. Our analysis found that the total harm caused by all the contaminants considered was not implausibly high, despite the possibility of 100% of the population being affected. Other studies have attempted to address this issue by adjusting the beta parameter or adding a lower percentage of exposure based on the time people spend indoors or at home. However, there is no consensus in the literature on this matter [1, 78, 79]. Despite our GSD being narrow, it is not implausible, as current GBD results indicate an even narrower range.

When addressing chronic harm in dwellings, we highlight that the most harmful contaminants are $PM_{2.5}$, $PM_{10–2.5}$, nitrogen dioxide, formaldehyde, radon, and ozone, accounting for 99.5% of the total median harm, and should be considered Contaminants of Concern. Our focus has been targeted toward influencing ventilation and acceptable IAQ standards, hence, much of our analysis considers ventilation's contribution to IAQ. While dilution ventilation is universally helpful, the contaminants of concern arise from diverse indoor and outdoor sources, requiring tailored removal strategies [2, 14, 80]:

- $PM_{2.5}$ - Formed from combustion (cooking, heating, smoking), outdoor air, reactions with gases. Ventilation filtration efficacious for removal.
- $PM_{10-2.5}$ - Resuspended dusts, indoor activities like cleaning. Enhanced filtration and dust control help reduce levels.
- NO_2 - Primarily outdoor origin, some gas appliances. Dilution ventilation effective, gas stove replacements beneficial.

- Formaldehyde - Off-gassing from materials/furnishings. Source control via low-emission materials impacts levels.
- Radon - Soil/rock source. Building shell mitigation and ventilation dilution are primary controls.
- Ozone - Outdoor origin. Dilution via ventilation lowers indoor levels. Air cleaning could assist.

B-5.2.1 Discussing harm from mold exposure.

In the main manuscript, we focused our discussion on the contaminants of concern, which collectively contribute to 99.5% of the total median harm among the full list of contaminants. However, in this section, we specifically address the issue of mold as it is an important contaminant deserving further attention. Mold emerges as the next contaminant, adding 0.2% to the overall harm, and its significance is underscored by its widespread presence in thousands of dwellings.

Exposure to mold contaminants has been shown to have a substantial impact on health, as highlighted by the WHO Europe Asthma burden study, which measured DALYs and deaths in 45 European countries for children (age 0-14) affected by indoor mold and dampness-related asthma [41]. Remarkably, the estimated harm from mold is comparable to our estimates for Radon and Ozone. However, it is essential to address the differences between our harm estimate and the WHO estimate, which reveals a one-order-of-magnitude discrepancy.

The primary divergence lies in the methodology used to define exposure to mold in dwellings. While the WHO relies on non-destructive (primarily visual) observation of mold as an indicator of microbial growth based on visible mold and/or mold odor, we measure actual concentrations of mold using colony-forming units (CFU) specifically for mold spores of the genus Cladosporium. Our approach may lead to an underestimation of mold harm due to the consideration of a single genome of mold in the environment. Furthermore, damp, and mouldy housing conditions likely involve multiple exposures that may contribute to the development of asthma, potentially resulting in an overestimation of asthma-related health effects.

Further research is necessary to better understand the relationship between qualitative and quantitative indicators of mold exposure and the corresponding quantification of harm. Exploring these aspects will provide insights into the accurate assessment of mold-related health risks and help bridge the gap between qualitative observations and quantitative measurements in mold exposure assessment.

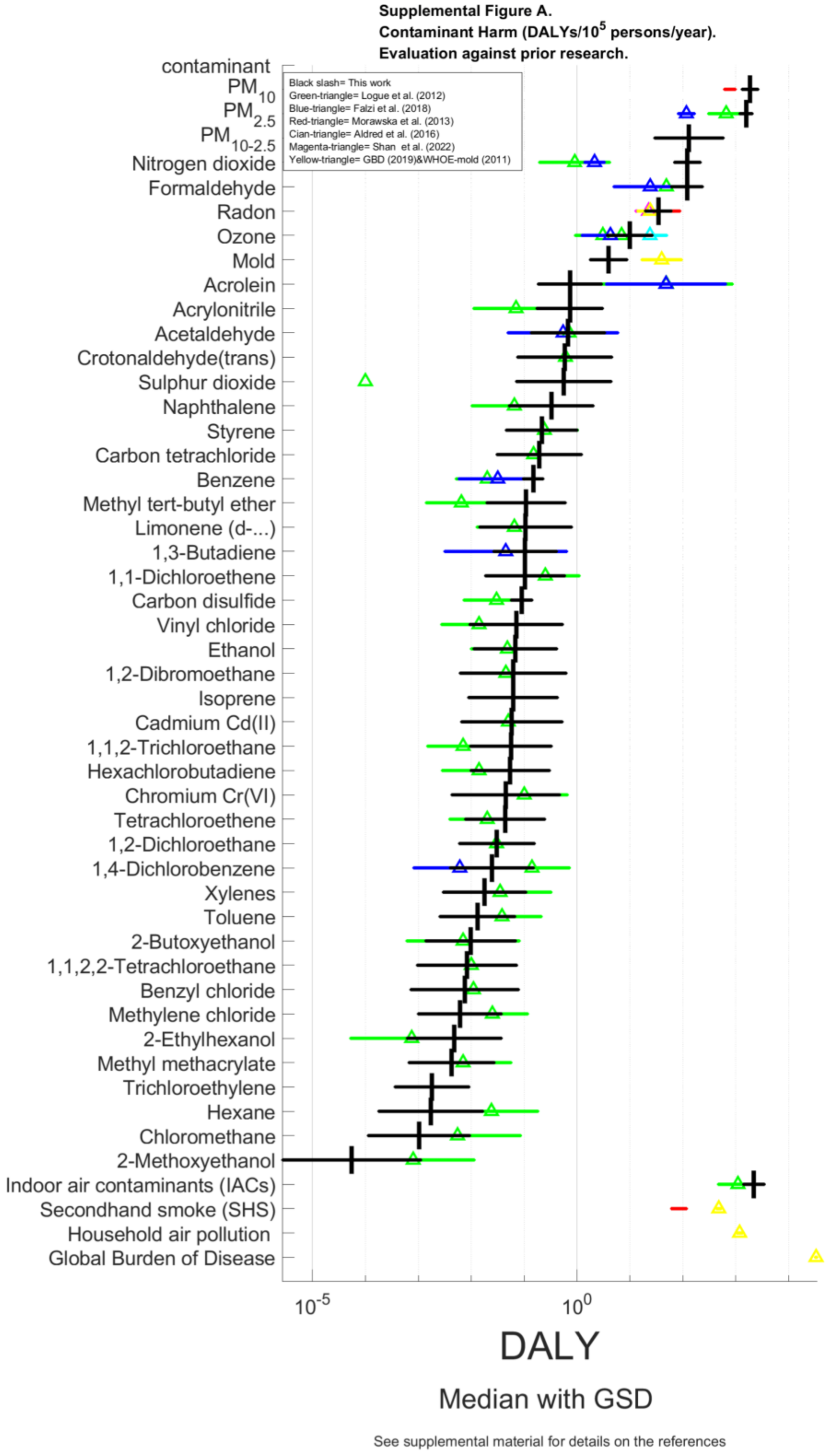

Supplemental Figure A. Harm. Evaluation against prior research.

Supplemental Table 7. Further details for studies included in Supplemental Figure A

B-6. Shape of the curve and regime of concentrations. Here, details regarding the relationship between the shape of the concentration – harm curves.

The shape of the Concentration-Response (C-R) function is the most important and uncertain parameter in the epidemiology-based approach [29]. Efforts have been made to explain the relative risk function for estimating mortality attributable to air contaminants, where $PM_{2.5}$ has received the most attention. Overall, there are three relative risk modelling approaches proposed: the log-linear model, the Global Burden of Disease integrated exposure–response model (GBD's IER) and the Global Exposure Mortality Model GEMM [30]. Choosing any of the functions will result in different results, hence, uncertainty remains about the true size of the $PM_{2.5}$ mortality relative risks. No such analysis has been performed in such depth for other air contaminants.

We applied a linear, no-lower-threshold, C-R relationship to model the harm ($Harm_i$) for all contaminants with epidemiology data: acrolein (C_3H_4O), benzene (C_6H_6), mold, formaldehyde (HCHO), nitrogen dioxide (NO_2), ozone (O_3), respirable particulate matter (PM_{10}), fine particulate matter ($PM_{2.5}$), Radon (Rn), and sulphur dioxide (SO_2). We recognize that a non-linear (usually log-linear) C-R relationship is applicable for the full range of ambient exposures, nonetheless those levels are outside the scope of study. A linearized curve is adequate for regimes of low concentrations. An assumption of linearity of effects at low exposures is often assumed appropriate for purposes of LCIA of airborne contaminants [27, 28, 34], and is further supported in chronic exposure risk assessments of child asthma diagnosis and HCHO [81], all-cause mortality and NO_2 [82, 83], mortality and O_3 (with less certainty, [84]), all-cause mortality and $PM_{2.5}$ [85], lung cancer mortality and Rn [86], and respiratory effects and SO_2 [87].

The C-R function of $PM_{2.5}$ is the most extensively studied. C-R curves vary for the same contaminant depending on the disease considered [88-90]. The shape of the total attributable mortality, which represents the sum of cause-specific mortalities, is influenced by each contaminant's individual C-R curve [90]. A universally accepted relationship has not yet been agreed upon, but the log-linear model, IER functions, and GEMM are still used, and their use is debated [30]. There are uncertainties in individual effects and contaminants, but the prevailing consensus in the literature is that linearity is appropriate at low concentrations. Therefore, the total harm follows a linear Dose-Response/Concentration-Response Curve.

In the existing research of airborne contaminants LCIA, the distance between the exposure concentration (known as the current state) and the point of zero impact (known as the state of zero impact) is modelled using three approaches: marginal, average, and linear effect models [5, 91]. In our

methodology, the state of zero impact (in the literature as: baseline background concentration or, theoretical minimal risk exposure level TMREL or, counterfactual level of exposure) was set to zero for all contaminants (no-lower-threshold) hence, our parameters follow the linear effect model. Above zero TMRELs for chronic exposure have been proposed for all-cause mortality and NO_2 [83], all-cause mortality and ozone [92], and mortality and $PM_{2.5}$ [30]. The TMRELs of these studies are based on statistical reasons (generally based the minimum and fifth percentile values of concentrations in the risk assessment) and any possible biological mechanisms explaining the reason of a level of pollution below which no effect would be seen, were not proposed.

For comparison, median and 95%CI of $\mathrm{Harm}_{\mathrm{I}}$ derived from both the log-linear (Equation (6)) and linear (Equation (8)) C-R relationships are shown in

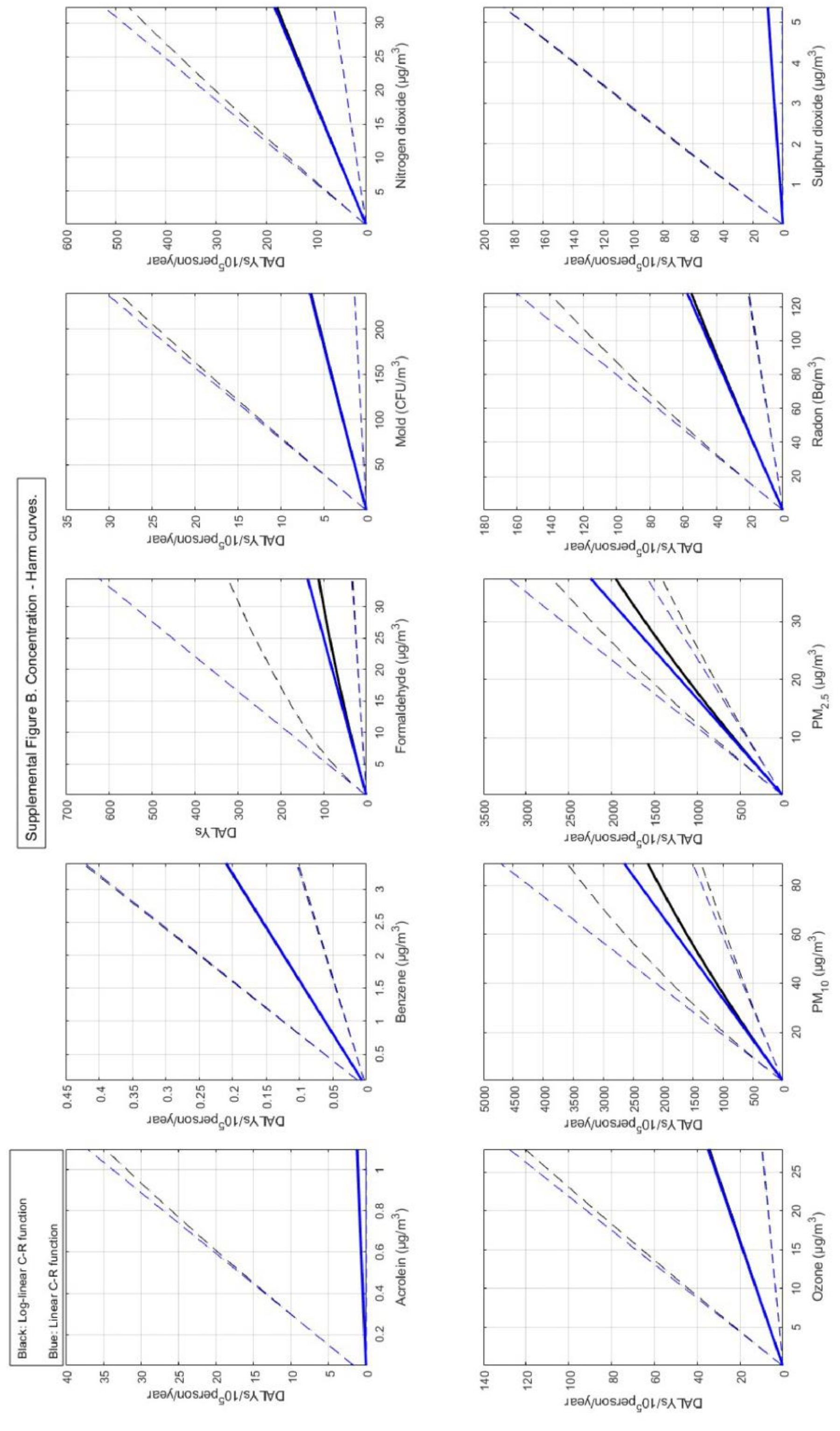
Supplemental Figure B. Concentration - Harm curves.
Black: Log-linear C-R function
Blue: Linear C-R function
DALYs/10^5 person/year
DALYs
Acrolein (μg/m^3)
Benzene (μg/m^3)
Formaldehyde (μg/m^3)
Mold (CFU/m^3)
Nitrogen dioxide (μg/m^3)
Ozone (μg/m^3)
PM10 (μg/m^3)
PM2.5 (μg/m^3)
Radon (Bq/m^3)
Sulphur dioxide (μg/m^3)

Supplemental Figure B. The wide of the x-axis represent the low concentrations regime for the contaminants. The upper limit of each x-axis was set as the 97.5th percentile of the distribution of the concentration.

The *I* from the linear C-R relationship appears as a straight line in the plots. The log-linear function is a sigmoid curve, which has a steep initial linear slope that then flattens out. Increasing exposure concentrations to extreme high values yield lower magnitudes of harm than the linear model, reflecting the influence of the saturation effect in a nonlinear exposure–response model.

Air pollution risk research has not precisely established quantitative definitions fo“ "l”w" an“ "hi”h" concentrations of contaminants. We can draw insights from the integrated exposure-response model for $PM_{2.5}$. A 30-50 μg/m^3 bracket potentially constitutes high exposure based on attributable disease burdens. Although, the risk C-R plots also show flattening of the curve at 100 or 300 μg/m^3 [93]. Moreover, a clear linear curve was employed for $PM_{2.5}$ mortality up to 30 μg/m^3 [94]. Further research and clearer definitions would aid risk analysis.

One potential approach to define low and high concentration thresholds is examining the divergence between linear and non-linear exposure-response models. Strong agreement between linear and non-linear fits may indicate a low exposure regime. Concentrations where models diverge above a chosen error level (e.g. 10% difference) could mark the transition to high exposures where non-linearities emerge. The level of disagreement between linear and non-linear shapes could provide a data-driven basis for delineating low versus high concentration thresholds, pending further analysis.

The log-linear function disagrees the most with a linear function for PM_{10}, $PM_{2.5}$, and HCHO. Evaluating the functions from zero to the upper percentile of the concentration, the mean absolute errors are >>>1, and the mean absolute percentage errors are close to 10% (considering the log-linear function as the reference). For the rest of contaminants, the log-linear models are linear for concentrations applied here, as expected (see

Supplemental Table 8). The concentration ranges align well, and these are acceptable concentrations regimes for applying a linear function versus the log-linear function.

Our constants should be applied cautiously: while the harm intensity may be suitable for low concentrations, a sensitivity analysis of the overestimation is warranted if they are applied for harm assessments at high exposure concentrations where the curve is attenuated by the saturation effect. Users of our values can decide on an acceptable level of disagreement or apply the log-linear function.

Supplemental Table 8. Disagreement between the linear and log-linear functions.

**Contaminant**$_{(i)}$	**Concentration Regime (**μg/m^3**)**	**Mean Absolute Error** (DALYs/10^{5}person/year)	**Mean Absolute Percentage Error** (%)
Acrolein	(– - 1.1)	0.040	4.5 (95%C.I. 1.8-10)
Benzene	(– - 3.4)	0.0010	0.45 (95%C.I. 0.024-1.1)
Formaldehyde	(– - 34)	11	10 (95%C.I. 9.6-12)
Mold	(– - 240)	0.015	1.0 (95%C.I. 0.27-2.9)
Nitrogen dioxide	(– - 32)	2.1	1.7 (95%C.I. 0.73-2.8)
Ozone	(– - 28)	0.050	0.94 (95%C.I. 0.28-2.6)
PM_{10}	(– - 89)	230	9.1 (95%C.I. 8.2-9.6)
$PM_{2.5}$	(– - 37)	100	7.4 (95%C.I. 7.1-7.9)
Radon	(– - 130)	1.1	2.6 (95%C.I. 1.7-3.5)
Sulphur dioxide	(– - 5.4)	0.31	2.0 (95%C.I. 0.41-5.7)

Radon in Bq/m^3. Mold in CFU/m^3.

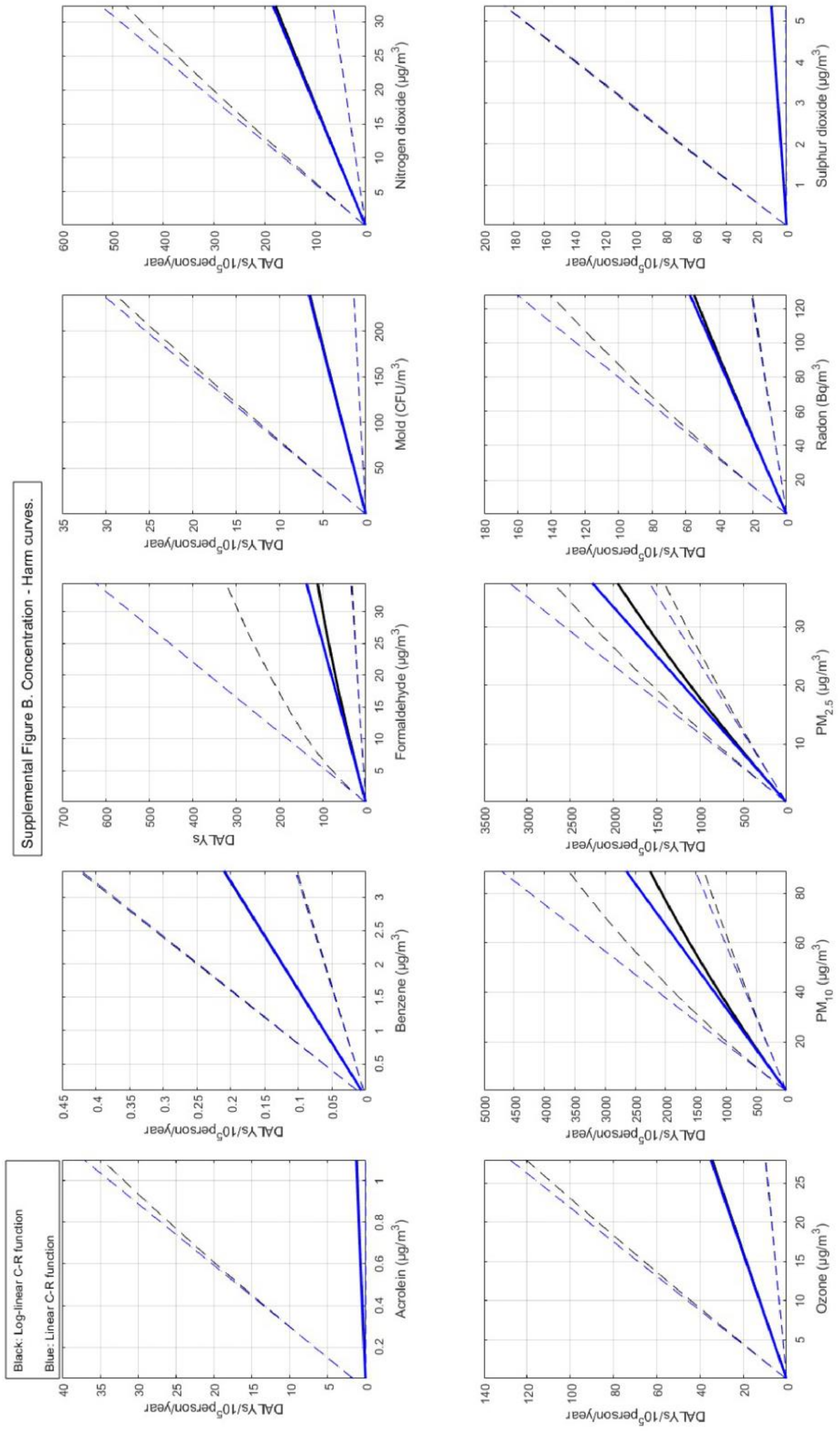

Supplemental Figure B. Concentration - Harm curves. .

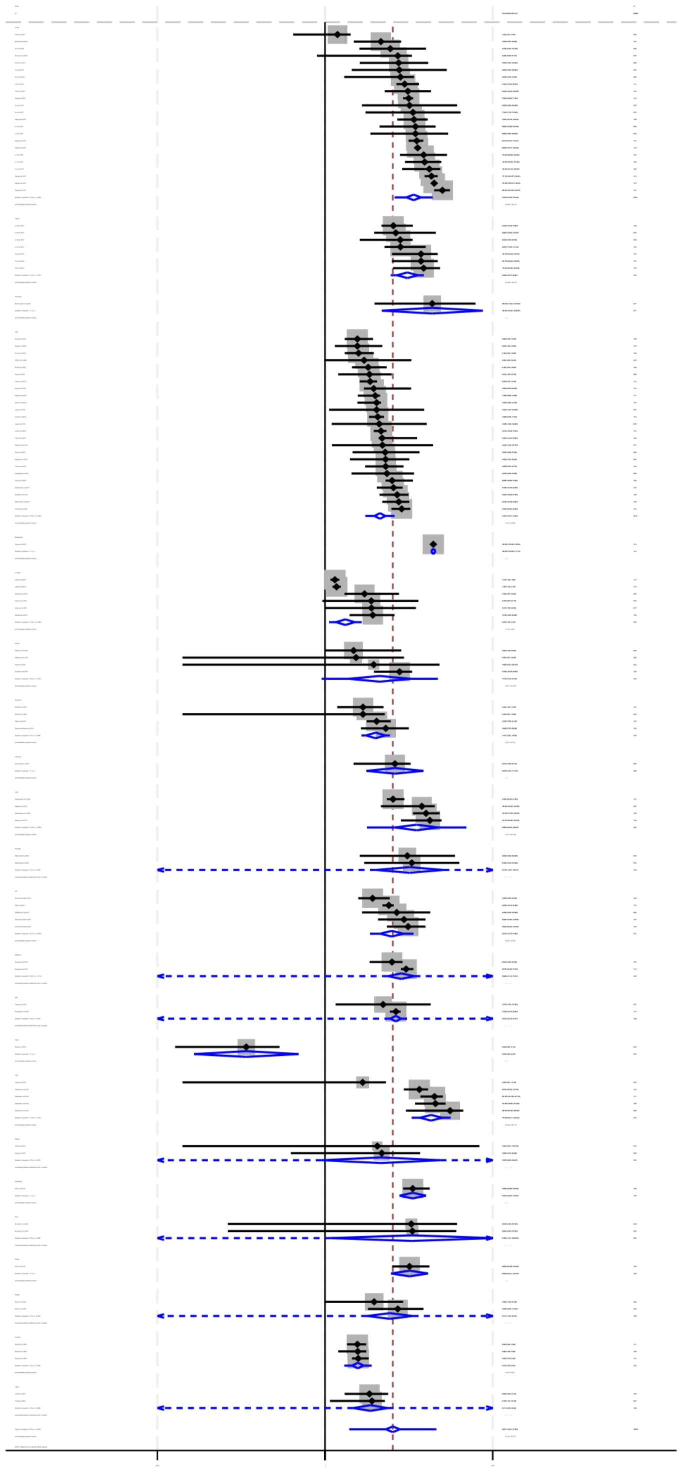

Representative indoor air concentration: PM2.5 -Subgroup Countryofstudy

Supplemental Figure C. Forest plots for meta-analysis of $PM_{2.5}$, sub-group by country or region.

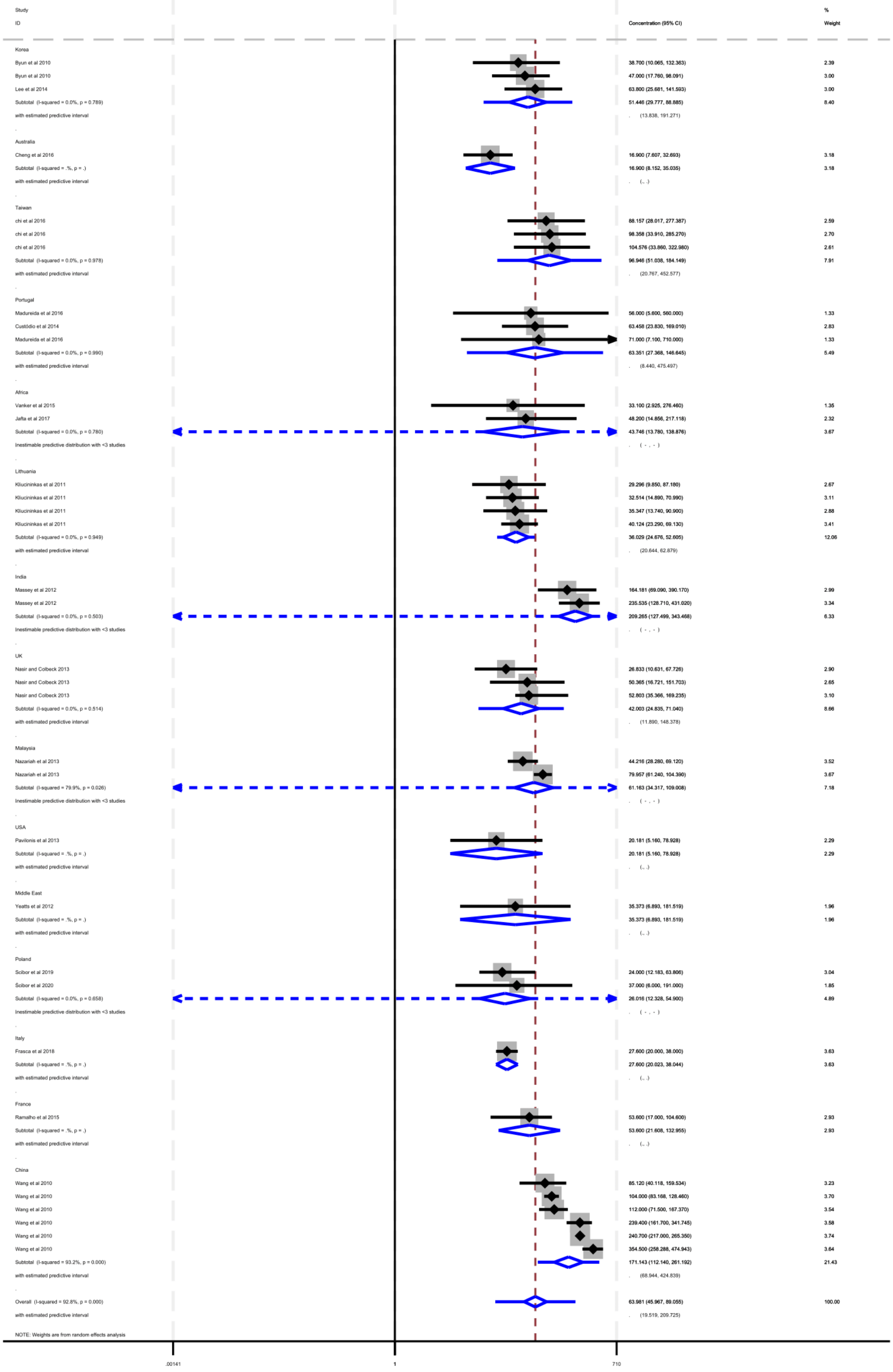

Representative indoor air concentration: PM10 -Subgroup Countryofstudy

577

578 Supplemental Figure D. Forest plots for meta-analysis of PM_{10}, sub-group by country or region.

Supplemental Table 9. Results from the systematic review and meta-analysis on indoor air contaminant concentration measurements in dwellings[9].

		Descriptive statistics from the systematic review								Meta-analysis results		
CAS	Contaminant	Mean	Standard deviation	Minimum	Maximum	Geometric mean	Geometric Standard deviation	Median	DataSets	Central tendency estimate	2.5ptile	97.5ptile
75-07-0	Acetaldehyde	22.496	29.392	6.800	143.000	15.127	2.533	16.765	36	14.882	7.308	30.307
107-02-8	Acrolein	2.477	3.485	0.100	14.750	1.018	4.724	1.150	20	0.646	0.370	1.101
107-13-1	Acrylonitrile	0.388	0.204	0.270	0.740	0.347	1.656	0.270	4	0.719	0.584	0.886
71-43-2	Benzene	3.265	2.680	0.850	11.910	2.205	2.323	2.090	65	2.301	1.561	3.391
100-44-7	Benzyl chloride	0.500	0.000	0.500	0.500	0.500	1.000	0.500	2	0.500	0.098	2.547
106-99-0	1,3-butadiene	0.879	1.046	0.050	3.140	0.446	1.420	0.460	11	0.461	0.269	0.792
111-76-2	2-Butoxyethanol	2.895	0.823	2.060	4.500	2.520	1.339	2.810	8	2.954	1.698	5.139
22537-48-0	Cadmium Cd(II)	0.024	0.024	0.003	0.070	0.014	3.435	0.018	5	0.015	0.005	0.042
75-15-0	Carbon disulfide	0.340	0.000	0.340	0.340	0.340	1.000	0.340	2	0.340	0.185	0.625
56-23-5	Carbon tetrachloride	0.548	0.532	0.077	2.200	0.350	2.741	0.410	18	0.515	0.368	0.721
78-87-3	Chloromethane	1.700	0.100	1.600	1.800	1.697	1.087	1.700	2	1.603	1.459	1.760
18540-29-9	Chromium Cr(VI)	0.006	0.004	0.002	0.011	0.005	3.020	0.006	2	0.006	0.002	0.027
123-73-9	Crotonaldehyde(trans)	1.857	1.918	0.090	5.600	0.853	4.319	1.060	13	0.799	0.337	1.899
106-93-4	1,2-Dibromoethane	0.375	0.431	0.006	0.980	0.094	13.085	0.140	3	0.096	0.008	1.160
106-46-7	1,4-Dichlorobenzene	15.413	26.008	0.050	120.000	5.304	10.026	2.800	30	2.201	1.055	4.591
107-06-2	1,2-Dichloroethane	0.728	0.888	0.003	2.700	0.291	5.035	0.250	21	0.530	0.380	0.728
75-35-4	1,1-Dichloroethene	0.339	0.241	0.018	0.600	0.163	6.809	0.400	3	0.512	0.304	0.862
64-17-5	Ethanol	348.043	287.889	55.600	860.000	230.889	2.812	288.000	7	129.163	66.726	250.026
104-76-7	2-Ethylhexanol	2.060	0.896	1.000	3.700	1.882	1.590	1.730	6	1.960	0.974	3.946
50-00-0	Formaldehyde	32.045	21.959	6.600	110.000	26.297	1.870	24.000	67	28.010	22.761	34.470

[9] Units in μg/m^3. Radon in Bq/m^3. Mold in CFU/m^3.

87-68-3	Hexachlorobutadiene	1.700	0.000	1.700	1.700	1.700	1.000	1.700	2	1.700	0.621	4.657
110-54-3	Hexane	2.971	2.726	0.100	10.700	1.787	3.436	1.800	19	1.657	0.829	3.312
78-79-5	Isoprene	5.330	4.521	1.600	15.800	3.977	2.198	3.640	8	6.483	3.880	10.833
5989-27-5	Limonene (d-...)	34.195	86.669	0.270	414.100	14.446	3.318	18.130	39	14.611	6.468	33.005
109-86-4	2-Methoxyethanol	43.015	60.659	0.120	128.800	1.245	55.552	0.125	4	1.270	0.009	174.001
80-62-6	Methyl methacrylate	0.270	0.000	0.270	0.270	0.270	1.000	0.270	2	0.270	0.034	2.125
1634-04-4	Methyl tert-butyl ether	7.170	4.999	0.100	15.700	4.126	4.953	4.600	8	4.367	1.653	11.536
75-09-2	Methylene chloride	2.171	2.828	0.100	8.200	0.824	5.127	0.977	6	0.908	0.324	2.549
NA	Mold	195.307	135.436	44.400	512.860	155.585	2.069	183.000	9	161.657	108.921	239.926
91-20-3	Naphthalene	32.711	117.734	0.179	503.000	1.633	7.382	0.850	19	1.541	0.536	4.429
10102-44-0	Nitrogen dioxide	37.517	51.990	2.800	242.520	23.280	4.626	22.936	48	22.443	15.568	32.357
10028-15-6	Ozone	14.730	15.630	1.340	58.560	9.456	5526.474	9.000	10	10.000	4.000	28.000
NA	PM_{10}	81.540	75.541	16.900	354.500	60.071	2.114	52.803	35	63.981	45.967	89.055
NA	[10]$PM_{10-2.5}$	29.489	NA							37.310	23.252	59.868
NA	$PM_{2.5}$	52.050	66.916	0.022	428.600	25.144	4.007	27.500	107	26.671	19.021	37.399
10043-92-2	Radon	126.455	105.024	32.000	358.000	91.720	2.292	60.300	10	82.546	53.172	128.149
100-42-5	Styrene	2.210	2.523	0.110	13.000	1.391	2.656	1.350	34	1.625	1.097	2.405
7446-09-5	Sulphur dioxide	5.886	8.977	0.090	26.200	1.368	18.934	1.400	8	0.969	0.176	5.366
79-34-5	1,1,2,2-Tetrachloroethane	0.239	0.185	0.005	0.420	0.099	8.085	0.266	4	0.088	0.017	0.450
127-18-4	Tetrachloroethene	0.924	0.801	0.000	2.900	0.646	2.760	0.583	21	0.835	0.695	1.003
100-88-33	Toluene	15.415	14.627	0.690	95.300	11.280	2.228	11.620	67	13.217	11.249	15.528
79-00-5	1,1,2-Trichloroethane	0.344	0.183	0.100	0.600	0.283	2.047	0.450	9	0.304	0.184	0.503
79-01-6	Trichloroethylene	0.436	0.536	0.015	1.800	0.229	3.344	0.240	20	0.456	0.387	0.538

[10]-We subtracted $PM_{2.5}$ concentrations from PM_{10} concentrations to calculate a (central tendency) concentration for $PM_{10-2.5}$. Uncertainty factor was calculated using equation (12) for PM fractions. We note that this is contributing to uncertainty in the interpretation of the coarse fraction, however, it is common practice [36. Sacks, J., et al., *Supplement to the 2019 integrated science assessment for particulate matter.* 2022.].
-The fraction of coarse PM within PM_{10} of 0.36 reported in the main manuscript is given by the mean column in this table.

75-01-4	Vinyl chloride	0.158	0.003	0.155	0.160	0.157	1.023	0.158	2	0.157	0.031	0.802
1330-20-7	Xylenes	7.663	2.868	1.400	13.300	6.895	1.739	7.700	13	7.014	4.832	10.182

Supplemental Table 10. Further details for studies included in Supplemental Figure A

Work	Statistics extracted	Spatial scope	Method	Contaminants	Concentrations from	Indoor
Logue et al. [1]	Median and GSD	USA	$\mathrm{Harm}_{k,i} = \gamma_{0_{k,i}} \cdot \left(1 - e^{-\beta_{k,i} \cdot C_i}\right) \cdot \mathrm{DF}_{k,i}$	Same 44 contaminants as our work	Systematic review	Dwellings
Fazli et al. [68]			$\mathrm{Harm}_{k,i} = \mathrm{mass}_{i} \cdot \left(\left(\mathrm{EF}_{cancer,i} \cdot \mathrm{ADAF}\right) + \mathrm{EF}_{non-cancer,i}\right)$	$PM_{2.5}$, nitrogen dioxide, formaldehyde, ozone, acrolein, acetaldehyde, benzene, 1,3-butadiene, 1,4-dichlorobenzene	Modelling	Residences
Aldred et al. [95]			$\mathrm{Harm}_{k,i} = \gamma_{0_{k}} \cdot \left(1 - e^{-\beta_{k,i} \cdot C_i}\right) \cdot \mathrm{DF}_{k}$	Ozone		Homes
Shan et al. [96]	Age-standardized disability-adjusted life rate and GSD	Global/ Europe	$\mathrm{Harm}_{k,i} = \mathrm{PAF}_{k,i} \cdot \mathrm{BoD}_{k}$	Radon	GBD database	Residential
Morawska et al. [69]	Minimum and maximum			Total particles, second-hand smoke, radon	No info	Indoor
WHOE-mold [41]	Central estimate and GSD			Mold	Do not apply	Home
GBD 2019 [17]				$PM_{2.5}$-household air pollution (from solid fuels), radon, second-hand smoke	Modelling	Household

BoD, Burden of Disease (the sum of DALYs across a population). health outcomes (k); contaminant (i).

Bibliography

1. Logue, J.M., et al., *A method to estimate the chronic health impact of air pollutants in U.S. residences.* Environmental Health Perspectives, 2012. **120**(2): p. 216-222.
2. Gonzalez-Martin, J., et al., *A state–of–the-art review on indoor air pollution and strategies for indoor air pollution control.* Chemosphere, 2021. **262**: p. 128376.
3. Huijbregts, M.A., et al., *Human-toxicological effect and damage factors of carcinogenic and noncarcinogenic chemicals for life cycle impact assessment.* Integrated Environmental Assessment and Management: An International Journal, 2005. **1**(3): p. 181-244.
4. Hauschild, M., R. Rosenbaum, and S. Olsen, *Life Cycle Assessment: Theory and Practice. 2018*. Springer, Cham.
5. Hauschild, M.Z. and M.A. Huijbregts, *Introducing life cycle impact assessment*, in *Life cycle impact assessment*. 2015, Springer. p. 1-16.
6. Fantke, P., et al., *Exposure and toxicity characterization of chemical emissions and chemicals in products: global recommendations and implementation in USEtox.* International Journal of Life Cycle Assessment, 2021. **26**(5): p. 899-915.
7. Crettaz, P., et al., *Assessing human health response in life cycle assessment using ED10s and DALYs: Part 1—Cancer effects.* Risk Analysis: AN INTERNATIONAL JOURNAL, 2002. **22**(5): p. 931-946.
8. Pennington, D., et al., *Assessing human health response in life cycle assessment using ED10s and DALYs: part 2—noncancer effects.* Risk Analysis: AN INTERNATIONAL JOURNAL, 2002. **22**(5): p. 947-963.
9. Fantke, P., et al., *USEtox® 2.0 Documentation (Version 1.00).* 2017.
10. Rosenbaum, R.K., et al., *USEtox—the UNEP-SETAC toxicity model: recommended characterisation factors for human toxicity and freshwater ecotoxicity in life cycle impact assessment.* The International Journal of Life Cycle Assessment, 2008. **13**(7): p. 532-546.
11. Jolliet, O., et al., *Establishing a framework for life cycle toxicity assessment. Findings of the Lausanne review workshop (4 pp).* The International Journal of Life Cycle Assessment, 2006. **11**(ARTICLE): p. 209-212.
12. Pennington, D., et al., *Risk and regulatory hazard-based toxicological effect indicators in life-cycle assessment (LCA).* Human and Ecological Risk Assessment, 2006. **12**(3): p. 450-475.
13. McKone, T.E., et al., *Dose-Response Modeling for Life Cycle Impact Assessment-Findings of the Portland Review Workshop.* 2006.
14. Tran, V.V., D. Park, and Y.-C. Lee, *Indoor air pollution, related human diseases, and recent trends in the control and improvement of indoor air quality.* International journal of environmental research and public health, 2020. **17**(8): p. 2927.
15. Turiel, I., *Indoor air quality & human health*. 2012: Routledge.
16. Murray, C.J., et al., *Global burden of 87 risk factors in 204 countries and territories, 1990–2019: a systematic analysis for the Global Burden of Disease Study 2019.* The lancet, 2020. **396**(10258): p. 1223-1249.
17. website, G.H.D.E., *Global Burden of Disease Study 2019 (GBD 2019) results*. 2019, Institute for Health Metrics and Evaluation (IHME), University of Washington ….
18. Reinoehl-Kompa, S. and M. Grunst, *Radon dose coefficients. Recommendation by the German Commission on Radiological Protection.* 2018.
19. Lecomte, J.-F., et al., *ICRP publication 126: radiological protection against radon exposure.* Annals of the ICRP, 2014. **43**(3): p. 5-73.
20. Phillips, L. and J. Moya, *The evolution of EPA's Exposure Factors Handbook and its future as an exposure assessment resource.* Journal of Exposure Science & Environmental Epidemiology, 2013. **23**(1): p. 13-21.
21. EPA, U., *Exposure Factors Handbook 2011 Edition (Final). Washington, DC.* 2011.

22. ., U.E.P.A. *Supplemental guidance for assessing susceptibility from early-life exposure to carcinogens (EPA/630/R-03/003F)*. 2005. US Environmental Protection Agency, Risk Assessment Forum Washington, DC.
23. OEHHA, C., *Technical Support Document for Cancer Potency Factors: Methodologies for derivation, listing of available values, and adjustments to allow for early life stage exposures*. 2009, California Environmental Protection Agency Sacramento, CA, USA.
24. Murray, C.J. and A.D. Lopez, *Global mortality, disability, and the contribution of risk factors: Global Burden of Disease Study.* The lancet, 1997. **349**(9063): p. 1436-1442.
25. Hassan Bhat, T., G. Jiawen, and H. Farzaneh, *Air pollution health risk assessment (AP-HRA), principles and applications.* International journal of environmental research and public health, 2021. **18**(4): p. 1935.
26. van Zelm, R., et al., *European characterization factors for human health damage of PM10 and ozone in life cycle impact assessment.* ATMOSPHERIC ENVIRONMENT, 2008. **42**(3): p. 441-453.
27. van Zelm, R., et al., *Regionalized life cycle impact assessment of air pollution on the global scale: Damage to human health and vegetation.* Atmospheric Environment, 2016. **134**: p. 129-137.
28. Gronlund, C.J., et al., *Characterizing the burden of disease of particulate matter for life cycle impact assessment.* Air Quality, Atmosphere and Health, 2015. **8**(1): p. 29-46.
29. Fantke, P., et al., *Global Effect Factors for Exposure to Fine Particulate Matter.* Environmental Science and Technology, 2019. **53**(12): p. 6855-6868.
30. Burnett, R. and A. Cohen, *Relative risk functions for estimating excess mortality attributable to outdoor PM2.5 air pollution: Evolution and state-of-the-art.* Atmosphere, 2020. **11**(6).
31. Nasari, M.M., et al., *A class of non-linear exposure-response models suitable for health impact assessment applicable to large cohort studies of ambient air pollution.* Air Quality, Atmosphere & Health, 2016. **9**(8): p. 961-972.
32. Logue, J., et al., *Hazard assessment of chemical air contaminants measured in residences.* Indoor air, 2011. **21**(2): p. 92-109.
33. Vardoulakis, S., et al., *Indoor exposure to selected air pollutants in the home environment: A systematic review.* International Journal of Environmental Research and Public Health, 2020. **17**(23): p. 1-24.
34. Huijbregts, M.A.J., et al., *ReCiPe2016: a harmonised life cycle impact assessment method at midpoint and endpoint level.* International Journal of Life Cycle Assessment, 2017. **22**(2): p. 138-147.
35. Organization, W.H., *WHO global air quality guidelines: particulate matter (PM2. 5 and PM10), ozone, nitrogen dioxide, sulfur dioxide and carbon monoxide*. 2021: World Health Organization.
36. Sacks, J., et al., *Supplement to the 2019 integrated science assessment for particulate matter.* 2022.
37. EPA, U., *Integrated science assessment for oxides of nitrogen–health criteria.* US Environmental Protection Agency, Washington, DC [Google Scholar], 2016.
38. EPA, U., *Integrated Science Assessment (ISA) of Ozone and Related Photochemical Oxidants. Research Triangle Park, NC: Office of Research and Development; 2020. EPA/600/R-20/012.* US Environmental Protection Agency, Washington, DC, 2020.
39. EPA, U., *Integrated Science Assessment (ISA) for Sulfur Oxides–Health Criteria (Final Report)*. 2017, Washington, DC: US Environmental Protection Agency, EPA/600/R-17/451.
40. Cohen, A.J., et al., *Estimates and 25-year trends of the global burden of disease attributable to ambient air pollution: an analysis of data from the Global Burden of Diseases Study 2015.* The lancet, 2017. **389**(10082): p. 1907-1918.
41. Braubach, M. and W.H. Organization, *Environmental burden of disease associated with inadequate housing: A method guide to the quantification of health effects of selected housing risks in the WHO European Region.* 2011.

42. Rojas-Rueda, D., et al., *Environmental burden of childhood disease in Europe.* International Journal of Environmental Research and Public Health, 2019. **16**(6): p. 1084.
43. Organization, W.H., *WHO global air quality guidelines: particulate matter (PM2. 5 and PM10), ozone, nitrogen dioxide, sulfur dioxide and carbon monoxide: executive summary.* 2021.
44. Richmond-Bryant, J., *In defense of the weight-of-evidence approach to literature review in the integrated science assessment.* Epidemiology (Cambridge, Mass.), 2020. **31**(6): p. 755.
45. Sacks, J.D., et al., *The Environmental Benefits Mapping and Analysis Program–Community Edition (BenMAP–CE): A tool to estimate the health and economic benefits of reducing air pollution.* Environmental Modelling & Software, 2018. **104**: p. 118-129.
46. Schmid, C.H., T. Stijnen, and I. White, *Handbook of meta-analysis*. 2020: CRC Press.
47. DerSimonian, R. and N. Laird, *Meta-analysis in clinical trials.* Controlled clinical trials, 1986. **7**(3): p. 177-188.
48. Daly, C. and C. Soobiah, *Software to Conduct a Meta-Analysis and Network Meta-Analysis.* Meta-Research, 2022: p. 223-244.
49. Fisher, D.J., *Two-stage individual participant data meta-analysis and generalized forest plots.* The Stata Journal, 2015. **15**(2): p. 369-396.
50. Harris, R.J., et al., *Metan: fixed-and random-effects meta-analysis.* The Stata Journal, 2008. **8**(1): p. 3-28.
51. Morawska, L., et al., *Airborne particles in indoor environment of homes, schools, offices and aged care facilities: The main routes of exposure.* Environment international, 2017. **108**: p. 75-83.
52. Ilacqua, V., et al., *Survey of residential indoor particulate matter measurements 1990–2019.* Indoor air, 2022. **32**(7): p. e13057.
53. Halios, C.H., et al., *Chemicals in European residences–Part I: A review of emissions, concentrations and health effects of volatile organic compounds (VOCs).* Science of The Total Environment, 2022: p. 156201.
54. Ott, W.R., *A physical explanation of the lognormality of pollutant concentrations.* Journal of the Air & Waste Management Association, 1990. **40**(10): p. 1378-1383.
55. Crow, E.L. and K. Shimizu, *Lognormal distributions*. 1987: Marcel Dekker New York.
56. Blackwood, L.G., *The lognormal distribution, environmental data, and radiological monitoring.* Environmental monitoring and assessment, 1992. **21**: p. 193-210.
57. Jia, C., J. D'Souza, and S. Batterman, *Distributions of personal VOC exposures: a population-based analysis.* Environment international, 2008. **34**(7): p. 922-931.
58. Shaked, S., et al., *Environmental life cycle assessment*. 2015: CRC Press.
59. Slob, W., *Uncertainty analysis in multiplicative models.* Risk analysis, 1994. **14**(4): p. 571-576.
60. Metropolis, N. and S. Ulam, *The monte carlo method.* Journal of the American statistical association, 1949. **44**(247): p. 335-341.
61. Moreno-Rangel, A., et al., *Indoor air quality in Passivhaus dwellings: A literature review.* International journal of environmental research and public health, 2020. **17**(13): p. 4749.
62. O'Leary, C., et al., *Investigating measurements of fine particle (PM 2.5) emissions from the cooking of meals and mitigating exposure using a cooker hood.* Indoor Air, 2019. **29**(3): p. 423-438.
63. Finley, B., et al., *Assessment of airborne hexavalent chromium in the home following use of contaminated tapwater.* Journal of Exposure Analysis and Environmental Epidemiology, 1996. **6**(2): p. 229-245.
64. Stark, P.C., et al., *Fungal levels in the home and allergic rhinitis by 5 years of age.* Environmental health perspectives, 2005. **113**(10): p. 1405-1409.
65. Matheson, M.C., et al., *Changes in indoor allergen and fungal levels predict changes in asthma activity among young adults.* Clinical & Experimental Allergy, 2005. **35**(7): p. 907-913.

66. Council, N.R., *Toxicologic assessment of the army's zinc cadmium sulfide dispersion tests.* 1997.
67. Canada, H., *Residential Indoor Air Quality Guidelines ACROLEIN For Public Consultation*. 2020: Water and Air Quality Bureau Health Canada.
68. Fazli, T. and B. Stephens, *Development of a nationally representative set of combined building energy and indoor air quality models for US residences.* Building and Environment, 2018. **136**: p. 198-212.
69. Morawska, L., et al., *Indoor aerosols: from personal exposure to risk assessment.* Indoor air, 2013. **23**(6): p. 462-487.
70. Nishihama, Y., et al., *Indoor air quality of 5,000 households and its determinants. Part A: particulate matter (PM2. 5 and PM10–2.5) concentrations in the Japan Environment and Children's Study.* Environmental Research, 2021. **198**: p. 111196.
71. Ye, W., et al., *Indoor air pollutants, ventilation rate determinants and potential control strategies in Chinese dwellings: a literature review.* Science of the Total Environment, 2017. **586**: p. 696-729.
72. Okello, G., G. Devereux, and S. Semple, *Women and girls in resource poor countries experience much greater exposure to household air pollutants than men: Results from Uganda and Ethiopia.* Environment international, 2018. **119**: p. 429-437.
73. Soneja, S., et al., *Humidity and gravimetric equivalency adjustments for nephelometer-based particulate matter measurements of emissions from solid biomass fuel use in cookstoves.* International journal of environmental research and public health, 2014. **11**(6): p. 6400-6416.
74. Zhang, T., et al., *Development of an approach to correcting MicroPEM baseline drift.* Environmental research, 2018. **164**: p. 39-44.
75. Morantes, G., et al., *A preliminary assessment of the health impacts of indoor air contaminants determined using the DALY metric.* International Journal of Ventilation, 2023: p. 1-10.
76. Martin, O.V., S. Martin, and A. Kortenkamp, *Dispelling urban myths about default uncertainty factors in chemical risk assessment–sufficient protection against mixture effects?* Environmental health, 2013. **12**(1): p. 1-22.
77. Xu, J., et al., *Exposure to Source-Specific Particulate Matter and Health Effects: a Review of Epidemiological Studies.* Current Pollution Reports, 2022. **8**(4): p. 569-593.
78. Underhill, L.J., et al., *Simulation of indoor and outdoor air quality and health impacts following installation of energy-efficient retrofits in a multifamily housing unit.* Building and Environment, 2020. **170**: p. 106507.
79. Milner, J., et al., *An exposure-mortality relationship for residential indoor PM2. 5 exposure from outdoor sources.* Climate, 2017. **5**(3): p. 66.
80. Mata, T.M., et al., *Indoor air quality: a review of cleaning technologies.* Environments, 2022. **9**(9): p. 118.
81. Lam, J., et al., *Exposure to formaldehyde and asthma outcomes: A systematic review, meta-analysis, and economic assessment.* PloS one, 2021. **16**(3): p. e0248258.
82. Huangfu, P. and R. Atkinson, *Long-term exposure to NO2 and O3 and all-cause and respiratory mortality: A systematic review and meta-analysis.* Environment international, 2020. **144**: p. 105998.
83. Organization, W.H., *Health risks of air pollution in Europe—HRAPIE project.* World Health Organization Regional Office for Europe Copenhagen, 2013. **20**.
84. Brown, J. and C. Bowman, *Integrated science assessment for ozone and related photochemical oxidants.* US Environmental Protection Agency: Washington, DC, USA, 2013.
85. Ross, M.A., *Integrated science assessment for particulate matter.* US Environmental Protection Agency: Washington DC, USA, 2009: p. 61-161.
86. Gaskin, J., et al., *Global estimate of lung cancer mortality attributable to residential radon.* Environmental health perspectives, 2018. **126**(5): p. 057009.

87. Johns, D.O. and W.S. Linn, *A review of controlled human SO2 exposure studies contributing to the US EPA integrated science assessment for sulfur oxides.* Inhalation toxicology, 2011. **23**(1): p. 33-43.
88. Burnett, R., et al., *Global estimates of mortality associated with long-term exposure to outdoor fine particulate matter.* Proceedings of the National Academy of Sciences, 2018. **115**(38): p. 9592-9597.
89. Apte, J.S., et al., *Addressing global mortality from ambient PM2. 5.* Environmental science & technology, 2015. **49**(13): p. 8057-8066.
90. Patel, V., et al., *Mental, neurological, and substance use disorders: disease control priorities, (volume 4).* 2016.
91. Heijungs, R., *The average versus marginal debate in LCIA: paradigm regained.* The International Journal of Life Cycle Assessment, 2021. **26**(1): p. 22-25.
92. Turner, M.C., et al., *Long-term ozone exposure and mortality in a large prospective study.* American journal of respiratory and critical care medicine, 2016. **193**(10): p. 1134-1142.
93. Burnett, R.T., et al., *An integrated risk function for estimating the global burden of disease attributable to ambient fine particulate matter exposure.* Environmental health perspectives, 2014. **122**(4): p. 397-403.
94. Pope III, C.A., et al., *Health benefits of air pollution abatement policy: Role of the shape of the concentration–response function.* Journal of the Air & Waste Management Association, 2015. **65**(5): p. 516-522.
95. Aldred, J.R., et al., *Benefit-cost analysis of commercially available activated carbon filters for indoor ozone removal in single-family homes.* Indoor Air, 2016. **26**(3): p. 501-512.
96. Shan, X., et al., *A global burden assessment of lung cancer attributed to residential radon exposure during 1990–2019.* Indoor air, 2022. **32**(10): p. e13120.
